# Structure-Based Rational Design of TcAgo from *Thermogladius calderae*

**DOI:** 10.3390/biom16050715

**Published:** 2026-05-13

**Authors:** Xiaochen Xie, Wanping Chen, Shi Chen, Tianxin Cai, Chendi Zhang, Jie Chen, Zhenni Xu, Zhuang Li, Longyu Wang, Lixin Ma

**Affiliations:** State Key Laboratory of Biocatalysis and Enzyme Engineering, Hubei Key Laboratory of Industrial Biotechnology, School of Life Sciences, Hubei University, Wuhan 430062, China; xiexc@stu.hubu.edu.cn (X.X.); wanpingchen@hubu.edu.cn (W.C.); 202421107011552@stu.hubu.edu.cn (S.C.); 202421107011660@stu.hubu.edu.cn (T.C.); chendizhang@stu.hubu.edu.cn (C.Z.); 202421107011927@stu.hubu.edu.cn (J.C.); 202221107011792@stu.hubu.edu.cn (Z.X.); zhuangli@hubu.edu.cn (Z.L.)

**Keywords:** thermophilic argonaute nucleases, TcAgo, cold adaptation, rational design, DNA-guided cleavage

## Abstract

Thermophilic prokaryotic Argonaute proteins (pAgos) have emerged as powerful tools for nucleic acid manipulation, with applications in nucleic acid detection, and DNA assembly. However, their strong dependence on high-temperature catalytic activity limits their utility under moderate conditions. TcAgo, a thermophilic Argonaute nuclease from *Thermogladius calderae*, exhibits efficient DNA-guided target DNA cleavage above 80 °C, yet its structural basis and catalytic mechanism remain unclear. In this study, we attempted to analyze the structure of the TcAgo ternary complex and performed rational engineering based on its structure and characteristics of cold-adapted enzymes. A mutant, mTcAgo (K574G, D577G), was obtained with enhanced activity at moderate temperatures. Compared with the wild type, mTcAgo exhibited significantly improved cleavage activity toward both DNA and RNA targets at 37 °C. It utilized multiple guide types, including 5′OH- and 5′P-modified DNA and RNA guides, with a preference for 5′P-gDNA. mTcAgo displayed optimal activity at pH 7–8, broad salt tolerance, and an extended catalytic temperature range from 37 °C to 95 °C. Notably, it retained high activity after incubation at 90 °C, with a melting temperature of ~88 °C, and efficiently cleaved GC-rich targets under low Mg^2+^ conditions. These results demonstrate that rational cold-adaptation engineering can expand the functional temperature range of thermophilic pAgos, providing a promising strategy for developing versatile nucleic acid tools.

## 1. Introduction

Argonaute (Ago) proteins constitute a diverse family of programmable nucleases widely distributed across all domains of life, where they play essential roles in gene regulation through binding small guide nucleic acids [1,2]. In contrast to their eukaryotic counterparts (eAgos), which primarily function in RNA interference pathways, prokaryotic Argonaute proteins (pAgos) exhibit substantial diversity in biochemical properties, including substrate specificity, guide preference, and catalytic mechanisms [3,4,5]. This functional versatility has positioned pAgos as promising tools for applications in synthetic biology and biotechnology. Unlike CRISPR-Cas systems, which require specific protospacer adjacent motifs (PAMs) [6] or protospacer flanking sequences (PFSs) [7] and are typically limited to RNA guides, many pAgos can mediate precise endonucleolytic cleavage without strict sequence constraints and are capable of utilizing both DNA and RNA as guides [8,9]. Recent research has shown that short pAgos possess the same trans-cleavage activity as the CRISPR system and can be used for nucleic acid detection [10]. This intrinsic flexibility provides significant advantages for programmable nucleic acid manipulation.

Despite these advantages, the broader application of pAgos is hindered by their biochemical limitations. Notably, many well-characterized pAgos, particularly those derived from thermophilic organisms such as *Thermus thermophilus* (TtAgo) [11,12] and *Pyrococcus furiosus* (PfAgo) [13], exhibit optimal catalytic activity only at elevated temperatures, typically above 75–90 °C. While such thermostability is beneficial for certain in vitro applications, it substantially restricts their use under physiological or moderate-temperature conditions required for in vivo applications and routine biochemical assays [14,15,16]. Consequently, developing pAgos with robust activity at lower temperatures has become a major research focus [17]. Recent approaches have included the identification of mesophilic pAgos and the application of protein engineering strategies; however, these efforts often result in reduced catalytic efficiency, limited substrate scope, or compromised stability. Encouragingly, recent studies suggest that integrating high-resolution ternary complex structures with rational cold-adaptation design strategies can effectively enhance pAgo performance. For example, engineered variants of PfAgo and TtdAgo (from *Thermococcus thioreducens*) have demonstrated significantly improved catalytic activity at 37 °C along with expanded substrate compatibility [18,19].

Structurally, most pAgos share a conserved bilobal architecture consisting of an N-terminal lobe (N and PAZ domains) and a C-terminal lobe (MID and PIWI domains) [18,20,21]. The MID and PAZ domains anchor the 5′ and 3′ ends of the guide nucleic acid, respectively, while the catalytic tetrad within the PIWI domain mediates endonuclease activity [21,22]. From an enzymological perspective, homologous enzymes from organisms adapted to different thermal environments often maintain comparable catalytic efficiencies at their respective physiological temperatures through adaptive mutations. These temperature-adaptive features are frequently located on the protein surface, distal to the active site, thereby preserving the integrity of the catalytic core [23,24,25,26,27]. In particular, cold-adapted enzymes tend to exhibit increased structural flexibility, often associated with a higher proportion of surface-exposed glycine residues, which can facilitate catalysis at lower temperatures through enhanced conformational dynamics [24,28,29,30].

TcAgo, a thermophilic Argonaute nuclease derived from the hyperthermophilic archaeon *Thermogladius calderae*, can mediate efficient DNA-guided target DNA cleavage and weak DNA-guided target RNA cleavage at high temperatures (Figure S2). However, its structural characteristics, catalytic mechanism, and potential for engineering remain largely unexplored. In this study, we aimed to elucidate the structural basis of TcAgo function and to expand its applicability through rational engineering. By combining structural analysis with a cold-adaptation design strategy, we generated a variant, mTcAgo, with markedly enhanced catalytic performance. Whereas wild-type TcAgo exhibits no detectable cleavage activity on DNA or RNA substrates at 37 °C, mTcAgo efficiently cleaves both single-stranded DNA and RNA at this temperature using multiple guide types. We further characterized its biochemical properties, including optimal reaction conditions, guide sequence and length, and thermostability. Notably, mTcAgo retains high thermal stability, maintaining efficient DNA cleavage activity after incubation at 90 °C for 20 min, while simultaneously acquiring robust activity at moderate temperatures. In addition, mTcAgo preferentially cleaves RNA targets when guided by 5′-phosphorylated DNA and can accurately process GC-rich regions under low Mg^2+^ concentrations. Collectively, these results demonstrate that targeted surface mutations can effectively broaden the functional temperature range of thermophilic TcAgo while preserving high catalytic efficiency and substrate versatility. This work provides new insights into the structure–function relationship of pAgos and establishes a rational framework for engineering programmable nucleases for diverse biochemical and biotechnological applications.

## 2. Materials and Methods

### 2.1. Molecular Cloning

The TcAgo gene was derived from the plasmid pET28a-TcAgo constructed in our laboratory. To generate TcAgo mutants, primers were designed based on the mutation sites. The gene fragments of the mutants were obtained by PCR [31] and inserted into the pET28a expression vector, yielding the pET28a-TcAgo mutant expression vectors with an N-terminal His tag. These recombinant plasmids were constructed using a DNA cloning method based on T5 exonuclease I and low-temperature operation [32]. The primers (Table 1) were synthesized by Shanghai Sangon Biological Engineering Technology & Services Co., Ltd. (Shanghai, China).

### 2.2. Protein Expression and Purification

*Escherichia coli* strain BL21(DE3) was used as the host for protein expression. A single colony was selected and inoculated into 5 mL of LK medium (LB medium containing 50 μg mL^−1^ kanamycin) and cultured overnight at 37 °C with shaking. The overnight culture was then transferred into 1 L of TB medium containing 50 μg mL^−1^ kanamycin at a 1:100 dilution. When the OD600 reached 0.6–0.8, isopropyl β-D-thiogalactopyranoside (IPTG) was added to a final concentration of 0.5 mM to induce protein expression, and the culture was incubated at 18 °C for 16–20 h. Cells were harvested by centrifugation at 6000× *g* for 10 min, and the supernatant was discarded. The cell pellets were resuspended in purification buffer (20 mM Tris-HCl pH 7.5, 400 mM NaCl, 5% glycerol), and phenylmethylsulfonyl fluoride (PMSF) was added to a final concentration of 1 mM. Cells were lysed using a high-pressure homogenizer, and the lysate was centrifuged at 14,000× *g* for 30 min. The supernatant was filtered through a 0.22 μm filter membrane. An appropriate amount of Ni-NTA resin was added to a gravity flow column, and the resin was washed with purification buffer and activated with purification buffer containing 10 mM imidazole. The filtered supernatant was loaded onto the column and allowed to bind for 50 min at 4 °C. The column was first washed with purification buffer containing 20 mM and 50 mM imidazole to remove impurity proteins, and then the target protein was eluted with purification buffer containing stepwise imidazole concentrations of 75, 100, 200, 300, 400, and 500 mM. The elution fractions were analyzed by SDS-PAGE, and fractions with high purity were pooled and concentrated by ultrafiltration using Amicon 50K filter units (Millipore, Burlington, MA, USA). The concentrated protein was further purified on a 5 mL Heparin HiTrap column (Cytiva, Marlborough, MA, USA). Protein concentration was determined using the Bradford Protein Assay Kit (Beyotime, Shanghai, China). The purified protein was flash-frozen in liquid nitrogen and stored at −80 °C.

### 2.3. Complex Assembly

Guides and target oligonucleotides (Table 2) were dissolved in TcAgo purification buffer. TcAgo protein was incubated with a 16 nt 5′-phosphorylated guide DNA in purification buffer supplemented with 1 mM Mg^2+^. Subsequently, a 17 nt target DNA was added to form the ternary complex. The molar ratio of TcAgo protein, guide, and target was 1:1.1:1.1. The reaction mixture was incubated at 80 °C with a final volume of 500 μL containing 2 mg of TcAgo protein. The assembled TcAgo ternary complexes were subjected to size-exclusion chromatography using a Superdex 200 Increase 10/300 GL column (Cytiva, Marlborough, MA, USA) equilibrated with purification buffer (20 mM Tris-HCl, pH 7.5, 250 mM NaCl). Elution fractions were collected and analyzed by SDS-PAGE, and nucleic acid incorporation was assessed by measuring the A_260_/A_280_ ratio. Fractions corresponding to the main chromatographic peak were pooled and used for subsequent structural analysis.

### 2.4. Electron Microscopy

Aliquots of 4.5 μL of the TcAgo-guide DNA-target DNA complex at a concentration of 0.7 mg mL^−1^ were applied to glow-discharged Quantifoil holey carbon grids (Au, R1.2/1.3, 200 mesh). Grids were blotted with a force of 2 for 10 s and plunged into liquid ethane using a Vitrobot (Thermo Fisher Scientific, Hillsboro, OR, USA). Cryo-EM data were collected on a Titan Krios microscope (FEI) operated at 300 kV. Images were recorded with EPU56 at a nominal magnification of 105,000× (corresponding to a calibrated physical pixel size of 0.85 Å/pixel) and a defocus range of −1.2 µm to −2.2 µm. A K3 Summit direct electron detector was used in super-resolution mode, coupled with a GIF-Quantum energy filter operated with a slit width of 20 eV. Images were acquired at a dose rate of 15 electrons/pixel/s over an exposure time of 2.5 s, yielding 40 movie frames and a total dose of approximately 54 e^−^/Å^2^.

### 2.5. Image Processing

The movie frames were imported into RELION-3.1, aligned using MotionCor2-1.6.4 with a binning factor of 2, and subjected to on-the-fly contrast transfer function (CTF) estimation using Gctf-1.06. For the TcAgo-guide DNA-target DNA dataset, 3.83 million particles were blob-picked and extracted from the dose-weighted micrographs. Following 2D classification, 1.87 million particles were selected. Particles from different views were used to generate initial models in cryoSPARC. Heterogeneous refinement (3D classification) was performed to distinguish different conformational states. A final set of 487,000 particles was used for 3D refinement, which converged to a resolution of 2.7 Å.

### 2.6. Model Building, Refinement, and Visualization

For high-resolution model building, the AlphaFold2-predicted structure of TcAgo was manually adjusted against the corresponding cryo-EM map using Coot-1.1.10. The guide DNA and target DNA were built de novo in Coot. Model refinement was performed using the phenix.real_space_refine tool in Phenix.

### 2.7. Structure-Based Cold-Adaptive Rational Design of TcAgo

The ternary complex structure of TcAgo was loaded into PyMOL-2.6. The six domains and bound DNA molecules were colored to facilitate identification. Amino acid residues located within 20 Å of the guide/target DNA and the DEDH catalytic tetrad were excluded from further consideration. The remaining surface-exposed residues in the PIWI domain were individually selected for substitution with glycine.

### 2.8. Cleavage Assay of Single-Stranded DNA and RNA

The guide and target nucleic acids were synthesized by Sangon Biotech and GenScript, respectively (Table S1). Unless otherwise specified, purified proteins, guide nucleic acids, and target nucleic acids were mixed at a molar ratio of 12.5:2:1 (12.5 pmol Ago, 2 pmol guide, and 1 pmol target) in a reaction buffer consisting of 10 mM HEPES-NaOH pH 8.0, 100 mM NaCl, 5 mM MnCl_2_, and 5% (*v*/*v*) glycerol. The protein was first pre-incubated with the guide DNA or guide RNA at 37 °C for 10 min to facilitate guide loading. Subsequently, the target DNA or RNA was added, and the mixture was incubated at 37 °C for 45 min to allow cleavage.

To investigate the effects of divalent metal ions on cleavage activity, MnCl_2_ in the reaction buffer was replaced individually with FeCl_2_, CoCl_2_, NiCl_2_, CuCl_2_, ZnCl_2_, CaCl_2_, MgCl_2_, or EDTA. To investigate the effects of pH or NaCl concentration on cleavage activity, reaction buffers with varying pH values (5–10) or NaCl concentrations (0–200 mM) were used in the cleavage reactions. Cleavage kinetics were determined by performing time-course reactions at 37 °C over a period of 0–60 min. The thermal stability of mTcAgo was evaluated by pre-incubating the protein with either 5′-OH-gDNA or 5′-P-gDNA at temperatures ranging from 25 °C to 95 °C for 20 min, followed by addition of the target nucleic acid to initiate cleavage. The temperature dependence of the cleavage reaction was analyzed by incubating samples at specified temperatures using a PCR thermal cycler (Bio-Rad, Hercules, CA, USA). All reactions were terminated by adding an equal volume of RNA loading dye (containing 95% formamide, 18 mM EDTA, 0.025% SDS, and 0.025% bromophenol blue) and heating at 95 °C for 5 min. Cleavage products were separated by 20% denaturing polyacrylamide gel electrophoresis and visualized using a Gel Doc XR+ imaging system (Bio-Rad).

ImageJ-1.53c, Microsoft Excel (2019), and Prism 8 (GraphPad, San Diego, CA, USA) were used for statistical analysis and graph generation. All experiments were performed in at least three independent replicates, and data are presented as the mean ± standard deviation (SD).

### 2.9. Guide-Directed Cleavage of Plasmid DNA by mTcAgo

For cleavage reactions using a single guide, 10 pmol of mTcAgo and 10 pmol of either the forward or reverse guide were used. For reactions with a pair of guides, two half-reactions were prepared, each containing 5 pmol of mTcAgo and 5 pmol of either the forward or reverse 5′-phosphorylated gDNA in a buffer composed of 10 mM HEPES (pH 7.5), 100 mM NaCl, 5% (*v*/*v*) glycerol, and 1 mM MnCl_2_. Each half-reaction was incubated at 70 °C for 10 min. Subsequently, the two half-reactions were mixed, and 200 ng of target plasmid was added. The mixture was then incubated at 75 °C.

The optimal Mg^2+^ concentration, reaction temperature, and reaction time for plasmid DNA cleavage by mTcAgo were determined. Supercoiled plasmids were tested with guides of varying GC content, using either single or multiple guide pairs. The guide sequences used are provided in Table 3. The cleavage products were mixed with 6× purple DNA loading dye and subjected to 0.8% agarose gel electrophoresis followed by EB staining. Results were visualized using a Gel Doc™ XR+ system.

### 2.10. DSF

For thermal stability analysis, 2 µM mTcAgo was prepared in triplicate in a buffer containing 5 mM Mg^2+^, 15 mM Tris-HCl (pH 8.0), and 250 mM NaCl, and aliquoted into PCR tubes. SYPRO Orange dye (5000× stock, Sigma-Aldrich, St. Louis, MO, USA) was added immediately before measurement to a final concentration of 5×. Thermal denaturation of mTcAgo was monitored by exciting the SYPRO Orange dye at 470 nm and measuring fluorescence emission at 570 nm using a real-time fluorescence quantitative PCR instrument (Bio-Rad).

## 3. Results

### 3.1. Overall Structure of TcAgo-Guide DNA-Target DNA Complex

Our previous structural data showed that, in the presence of Mg^2+^, PfAgo assembled with a 16 nt 5′-phosphorylated guide DNA (5′-phos-TGAGGTAGTAGGTTGT) and a 17 nt target DNA (ACAACCTACTACCTCAT) to form a ternary complex. This complex existed predominantly as a dimer, and dimerization was entirely mediated by PfAgo itself. Given the 20.04% sequence identity between TcAgo and PfAgo, we attempted a similar assembly strategy using TcAgo with a 16 nt guide DNA and a 17 nt target DNA, and successfully determined its structure at 2.7 Å resolution (Figure S1).

**Figure 1:**
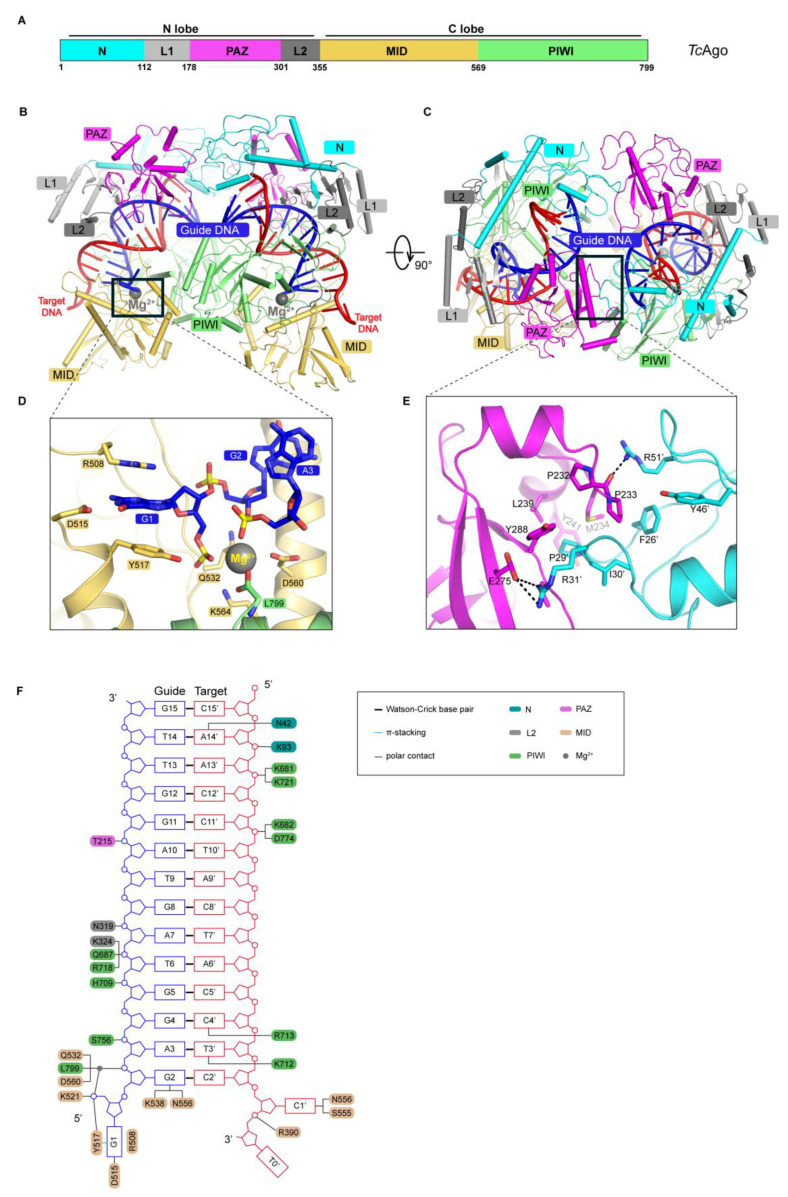
Structure of TcAgo-guide DNA-target DNA ternary complex. (**A**): Domain architecture of TcAgo. (**B**): Cartoon representation of TcAgo-guide DNA-target DNA atomic model. (**C**): The other view of (**B**). (**D**): Interaction details on PIWI-MID interfaces. (**E**): Interaction details on PAZ-N interfaces. (**F**): Schematic of all the possible interactions between TcAgo and guide DNA-target DNA duplex.

At this resolution, we were able to build ab-initio models for all protein residues, the magnesium ions near the catalytic site and the 5′ end of the guide DNA, and unambiguously assign all nucleotides. TcAgo adopts a bilobed architecture and can be divided into the N domain (residues 1–112), interdomain linker L1 (112–178), PAZ domain (178–301), interdomain linker L2 (301–355), MID domain (355–569), and PIWI domain (569–799) (Figure 1A).

Structural analysis revealed that upon guide and target binding, the MID domain forms extensive interactions around the 5′ end of the guide DNA. G1 makes polar contacts with R508 and D515, while also engaging in π-π stacking with Y517. Together, these residues form a core recognition region for the guide 5′ end and play a key role in guide loading (Figure 1D). In addition, K538 and N556 form polar contacts with G2, further stabilizing the proximal region of the guide 5′ end.

Surrounding the 5′ phosphate group of the guide DNA, we observed a polar interaction network involving Y517, K521, Q532, D559, and L799, with one Mg^2+^ directly coordinating the 5′-phosphate in a typical metal-dependent stabilization mode (Figure 1D,F). These interactions collectively constitute the 5′-anchor system for the guide DNA. Rotation of the complex revealed the dimeric interface of TcAgo (Figure 1B,C), the PAZ domain of one monomer directly contacts the N domain of the other monomer, forming a stable interfacial network. A closer look at the interface shows that E275 forms polar interactions with P29′ and R31′ from the opposing monomer (′was used to denote the amino acid from the other TcAgo), while P232 and P233 make additional polar contacts with R51′ from the opposing monomer (Figure 1C,E). These intermolecular interactions suggest that dimerization is not a simple spatial aggregation but rather a specific assembly mediated by a network of amino acid interactions, which may be functionally important for maintaining the catalytic conformation.

A schematic overview of the TcAgo-guide-target complex shows extensive and continuous intermolecular contacts along the guide-target duplex from positions 2 to 15, including hydrogen bonds, electrostatic interactions, and stacking interactions involving both backbone and side-chain groups (Figure 1F). This multipoint interaction network distributed along the duplex not only stabilizes the overall complex but also provides the structural basis for proper positioning of the catalytic site and subsequent substrate cleavage.

### 3.2. Structure-Based Rational Design of Cold Adaptation of TcAgo

**Figure 2:**
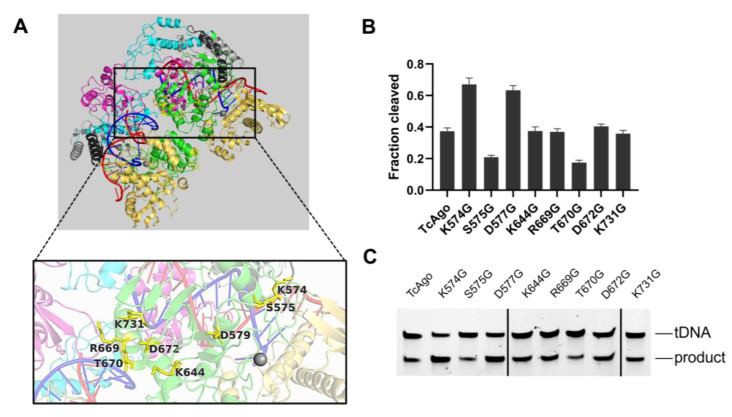
Rational engineering of TcAgo generates mutants with high cleavage activity at 70 °C. (**A**): Structure of TcAgo ternary complex with the mutation sites marked. (**B**): Quantitative comparison of ssDNA cleavage activity among wild-type TcAgo and its mutants. (**C**): Gel electrophoresis was used to detect the cleavage of ssDNA by wild-type TcAgo and its mutants. The original electrophoretic gels images can be found in Supplementary Materials.

We performed a structure-guided rational design of TcAgo based on four principles [19]: (a) the residue side chain is highly solvent-exposed and does not interact with the protein interior; (b) the residue is located distal (>20 Å) from both the catalytic site and the nucleic acid substrates (guide and target strands); (c) the mutated residue is situated within the PIWI domain; and (d) the residue is substituted with glycine (Figure 2A, Table 1).

Here, the substrates refer to the guide and target nucleic acids, whereas the catalytic site corresponds to the DEDH tetrad. Structural analysis of the TcAgo ternary complex using PyMOL identified eight candidate residues, each of which was individually mutated to glycine (Figure 2A). The catalytic activities of these variants were evaluated at 70 °C using 5′-phosphorylated guide DNA (5′P-gDNA). Among the eight mutants, three exhibited enhanced activity relative to the wild-type protein, with TcAgo_K574G and TcAgo_D577G showing the most pronounced increase in cleavage efficiency (Figure 2B, C).

**Figure 3:**
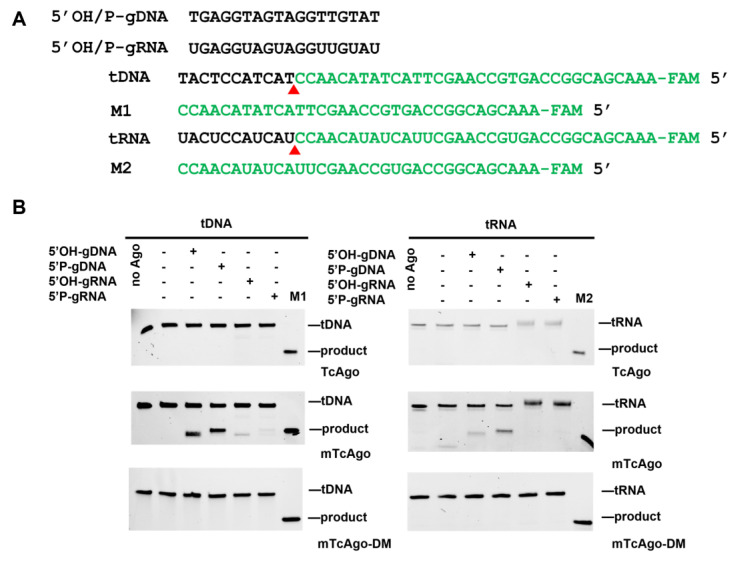
Cleavage activity assay of TcAgo mutants at 37 °C. (**A**): The oligonucleotides sequence of guides and targets. (**B**): The gel diagram showing TcAgo mutants using 5′FAM-labeled DNA and RNA as targets, respectively. M1 indicates synthesized 5′FAM-labeled, 34 nt DNA product. M2 indicates synthesized 5′FAM-labeled, 34 nt RNA product, no Ago represents a negative control without Ago proteins. The original electrophoretic gels images can be found in Supplementary Materials.

To further improve catalytic performance, we combined these two mutations to generate a double mutant, called mTcAgo (K574G/D577G). The guide preferences and substrate scope of mTcAgo were then assessed at 37 °C using four types of guides (5′OH-gDNA, 5′P-gDNA, 5′OH-gRNA, and 5′P-gRNA) against complementary DNA or RNA targets (Figure 3A).

Wild-type TcAgo cannot cleave either tDNA or tRNA at 37 °C, mTcAgo exhibited robust cleavage activity toward both DNA and RNA targets when guided by 5′P-gDNA. Cleavage of DNA targets was also observed with 5′OH-gDNA, whereas both 5′OH-gRNA and 5′P-gRNA were capable of directing DNA cleavage. In addition, mTcAgo could cleave RNA targets when guided by 5′OH-gDNA, albeit with lower efficiency. The catalytic residues D585, E624, D653, and D774 constitute the active site of mTcAgo. A catalytically inactive variant (mTcAgo-DM, D585A/D653A) completely lost cleavage activity, confirming the essential role of the DEDX catalytic tetrad within the PIWI domain (Figure 3B). Collectively, these findings demonstrate that the engineered mTcAgo achieves robust catalytic activity under moderate-temperature conditions while possessing broader substrate versatility.

### 3.3. Effect of Buffer Systems on the Cleavage Activity of mTcAgo

To systematically evaluate the DNA-guided cleavage activity of mTcAgo at 37 °C, we measured its cleavage activity against target DNA and RNA under various conditions. In general, the optimal reaction conditions for an enzyme are related to the growth environment of its native host.

**Figure 4:**
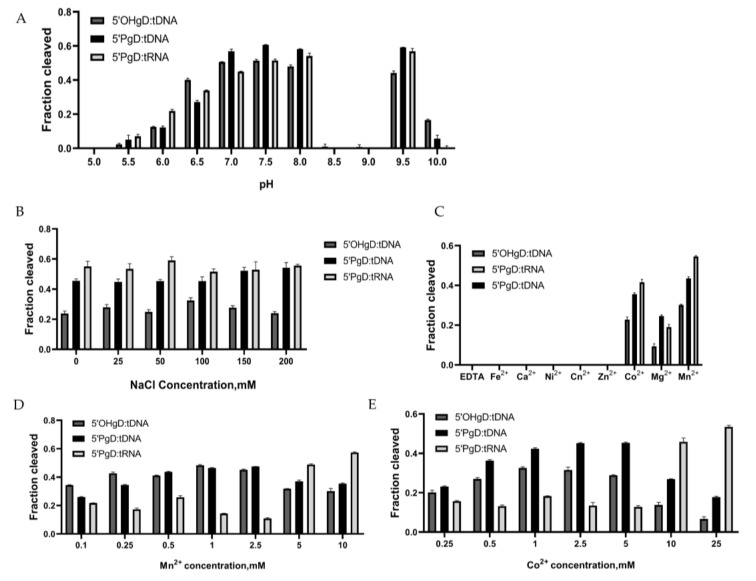
Effect of Buffer Systems on the Cleavage Activity of mTcAgo at 37 °C. The effects of pH (**A**), NaCl concentration (**B**), divalent metal ion (**C**), Mn^2+^ concentration (**D**), and Co^2+^ concentration (**E**) on the DNA and RNA cleavage activity of mTcAgo with gDNA were examined at 37 °C (*n* = 3). Data are presented as the mean ± SD.

We first examined the cleavage of target DNA or RNA by mTcAgo guided by 5′P-gDNA across a range of pH values and NaCl concentrations. Whether DNA or RNA target, mTcAgo exhibited efficient cleavage activity at pH 6.5–8.0 and at pH 9.5 (Figure 4A and Figure S3A). An unexpected observation was that mTcAgo exhibited no detectable cleavage activity at pH 8.0–8.5, regained activity at pH 9.5, and lost activity again at pH 10.0. We found that the isoelectric point (pI) of mTcAgo is 8.4, and visible protein precipitation occurred in the reaction mixture at pH 8.5–9.0. Thus, we attribute the activity loss near pH 8.4–9.0 to protein aggregation caused by minimal net charge and low solubility near the pI. Meanwhile, mTcAgo showed comparably high cleavage activity against both DNA and RNA targets at NaCl concentrations between 50 and 100 mM (Figure 4B and Figure S3B). Given the essential role of divalent metal ions in the binding of 5′P-guides to Ago proteins and in their catalytic activity [14], we next assessed the 5′P-DNA-guided cleavage activity of mTcAgo toward DNA or RNA targets in the presence of various divalent metal ions. Cleavage of both DNA and RNA was detected with Co^2+^, Mg^2+^ or Mn^2+^, with more efficient cleavage observed in the presence of Mn^2+^ (Figure 4C and Figure S3C). We then further investigated the effects of different Co^2+^ or Mn^2+^ concentrations on this cleavage activity. Co^2+^ titration experiments showed that mTcAgo cleaved targets across a concentration range of 0.25 to 25 mM, with maximum cleavage efficiency for tDNA at 1 mM and for tRNA at 25 mM (Figure 4E and Figure S3D). Mn^2+^ titration experiments demonstrated that mTcAgo cleaved targets across a concentration range of 0.1 to 10 mM, reaching peak cleavage efficiency for tDNA at 1 mM and for tRNA at 10 mM (Figure 4D and Figure S3D).

### 3.4. Effect of Guide DNA on the Cleavage Activity of mTcAgo

**Figure 5:**
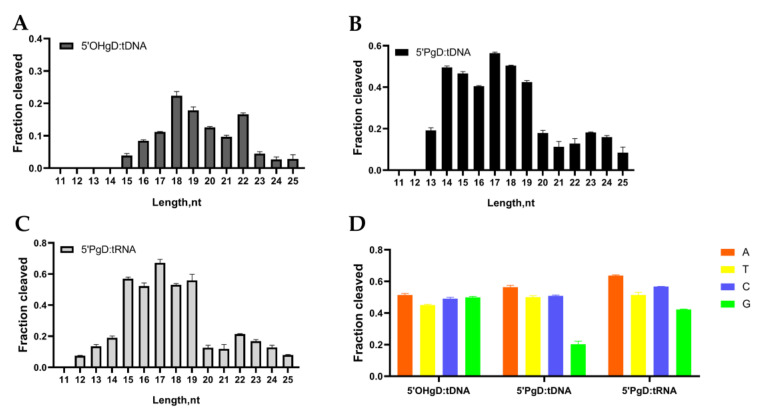
Effect of guide DNA on the Cleavage Activity of mTcAgo at 37 °C. The effects of 5′OH-gDNA length (**A**), 5′P-gDNA length (**B**), 5′P-gDNA length (**C**), and 5′-end nucleotide of gDNA (**D**) on the DNA and RNA cleavage activity of mTcAgo with gDNA were examined at 37 °C (*n* = 3). Data are presented as the mean ± SD.

In addition, we examined the effects of gDNA length on the DNA or RNA cleavage activity of mTcAgo. gDNAs of 11–25 nt in length with identical 5′-terminal sequences were used in the cleavage experiments (Table S1). The results showed that mTcAgo required 5′OH-gDNA of at least 15 nt to cleave tDNA (Figure 5A and Figure S4A). When the 5′OH-gDNA length was 18, 19, or 22 nt, mTcAgo exhibited high cleavage activity, with the highest activity observed with the 18 nt long guide (Figure 5A). mTcAgo also cleaved tDNA using 5′P-gDNA as short as 13 nt, with higher cleavage activity observed with 17 and 18 nt long guides (Figure 5B). For RNA cleavage, mTcAgo cleaved tRNA using 5′P-gDNA as short as 12 nt, with better cleavage activity observed with guides of 15–19 nt in length and the highest activity observed with the 17 nt long 5′P-gDNA (Figure 5C).

Many Ago proteins exhibit a preference for the 5′-end nucleotide of guides in various contexts, such as the binding of endogenous nucleic acids in vivo [1], structure-based guide binding [33,34], and in vitro cleavage activity [35,36]. To investigate the influence of the 5′-end nucleotide of gDNA on the DNA and RNA cleavage activity of mTcAgo, we synthesized a series of gDNAs with different 5′-end nucleotides but otherwise identical sequences (Table S1). The results showed that when mTcAgo used 5′OH-gDNA to cleave complementary tDNA, comparable cleavage efficiency was observed among guides with 5′-A, 5′-T, 5′-C, and 5′-G, and no obvious preference for the 5′-end nucleotide was detected (Figure 5D and Figure S4B). When mTcAgo cleaved tDNA and tRNA guided by 5′P-gDNA, the highest cleavage efficiency was observed with the 5′-A guide, followed by the 5′-T and 5′-C guides, whereas significantly lower activity, with the weakest observed for the 5′-G guide. Together, these results demonstrate that mTcAgo exhibits efficient DNA and RNA cleavage activity at 37 °C with different length and 5′-end nucleotide of guide.

### 3.5. Effect of Guide/Target Single-Base Mismatch on the Cleavage Activity of mTcAgo

Single-base mismatches between guide and target nucleic acids can differentially affect the cleavage activity of Argonaute proteins, highlighting their potential utility in molecular diagnostics. To evaluate the mismatch tolerance and target specificity of mTcAgo, we selected an 18 nt 5′A-gDNA that was previously identified as the optimal guide in our system. Single-nucleotide substitutions were systematically introduced at each position along the guide strand (positions 1–18) by replacing each base with its complementary counterpart (A↔T and G↔C). The resulting guide variants were designated Mismatch 1 (m1) through Mismatch 18 (m18) (Table S1).

**Figure 6:**
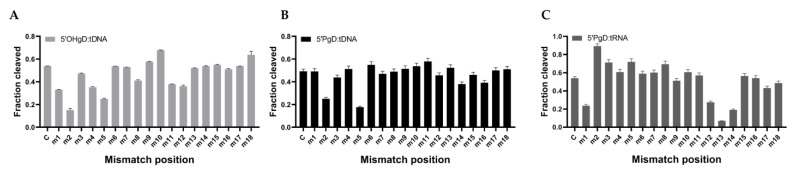
Effect of 5′OH-gDNA/tDNA (**A**), 5′P-gDNA/tDNA (**B**), 5′P-gDNA/tRNA (**C**), single base mismatch on the cleavage activity of mTcAgo (*n* = 3). Data are presented as the mean ± SD.

The effects of single-base mismatches on mTcAgo cleavage activity were position-dependent (Figure S5). When mTcAgo targeted tDNA with 5′OH-gDNA, mismatches at positions 6, 14, and 17 had minimal impact on cleavage activity. In contrast, mismatches at positions 9, 10, 15, and 18 led to enhanced activity, with a mismatch at position 10 resulting in a marked increase in cleavage efficiency. Mismatches at the remaining positions reduced activity, with the most pronounced decrease observed at position 2 (Figure 6A). When mTcAgo targeted tDNA guided by 5′P-gDNA, only mismatches at positions 2, 3, 5, 14, and 16 significantly impaired cleavage activity, among which a mismatch at position 5 caused the most substantial reduction (Figure 6B). For tRNA cleavage guided by 5′P-gDNA, mTcAgo exhibited relatively high tolerance to single-base mismatches. Significant reductions in activity were observed only for mismatches at positions 1, 12, 13, and 14, with a mismatch at position 13 nearly abolishing cleavage activity (Figure 6C).

### 3.6. Effect of Temperature on the Cleavage Activity and Thermal Stability of mTcAgo

**Figure 7:**
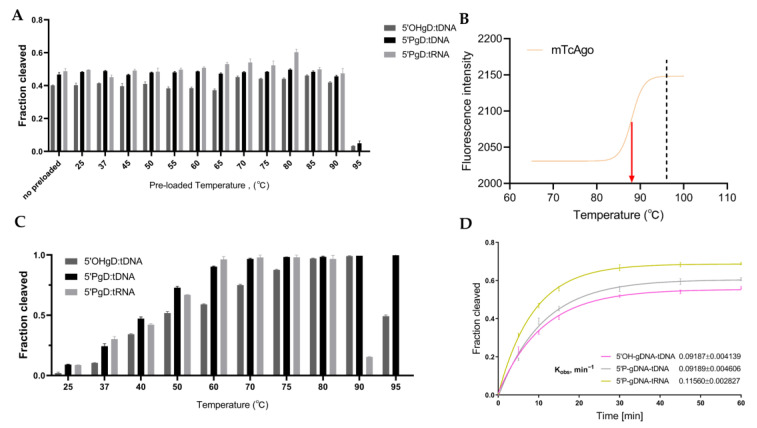
Effect of Temperature on the Cleavage Activity and Thermal Stability of mTcAgo. (**A**): Effect of gDNA loaded temperatures on the cleavage activity of mTcAgo at 37 °C. Data are presented as the mean ± SD. (**B**): Thermal unfolding curves of mTcAgo through DSF measurement. The fluorescence intensity is plotted as a function of temperature and fitted using a Boltzmann sigmoidal model. The red arrow and dashed line represent T_on_ and T_phy_, respectively. (**C**): Quantitative analysis of mTcAgo cleavage activity at different temperatures. (**D**): Kinetics of target cleavage by mTcAgo. Data are fitted to a single exponential equation, and the resulting *k*_obs_ value is shown for each guide-target pair.

To obtain mutant proteins with both thermal stability and low-temperature catalytic activity after cold adaptation, we further investigated the effects of temperature on the cleavage activity and thermal stability of mTcAgo using the optimal reaction system. First, mTcAgo and guide DNA were incubated together at various temperatures (25–95 °C) for 20 min, followed by detection of tDNA and tRNA cleavage at 37 °C (Figure S4A). After incubation at 90 °C, mTcAgo retained efficient cleavage activity toward both targets; however, activity was largely lost after incubation at 95 °C (Figure 7A), with a calculated Tm of 88 °C (Figure 7B). In addition, mTcAgo exhibited RNA cleavage activity across a temperature range of 25–80 °C (Figure 7C). Activity gradually increased with rising temperature, with nearly complete target cleavage observed at 60–80 °C. A trace amount of tRNA cleavage activity was still retained at 90 °C. In addition, mTcAgo possessed 5′OH-DNA-guided DNA cleavage activity between 25 and 95 °C. When targeting tDNA with 5′OH-gDNA at 95 °C, the activity of mTcAgo dropped sharply. Notably, when targeting tDNA with 5′P-gDNA, mTcAgo displayed cleavage activity across the entire temperature range of 25–95 °C, with activity increasing as temperature increased, and comparable activity levels were observed between 70 and 95 °C (Figure S6B).

With these optimized cleavage conditions in hand, the DNA and RNA cleavage kinetics of mTcAgo were measured at 37 °C using 5′OH-gDNA or 5′P-gDNA as guides, respectively. The fastest reaction rate was observed for 5′P-gDNA-mediated tRNA cleavage, with a *k*_obs_ value of 0.116 ± 0.003 (Figure 7D). For tDNA cleavage, both the reaction rate and the final cleavage extent were higher with 5′P-gDNA (*k*_obs_ = 0.092 ± 0.005) than with 5′OH-gDNA (*k*_obs_ = 0.092 ± 0.004). In summary, the 5′P-gDNA-mediated cleavage efficiency toward tDNA was lower than that toward tRNA, indicating that mTcAgo prefers utilizing 5′P-gDNA to cleave complementary tRNA (Figure S6C).

### 3.7. Cleavage of Plasmid DNA by the mTcAgo-gDNA Complex

**Figure 8:**
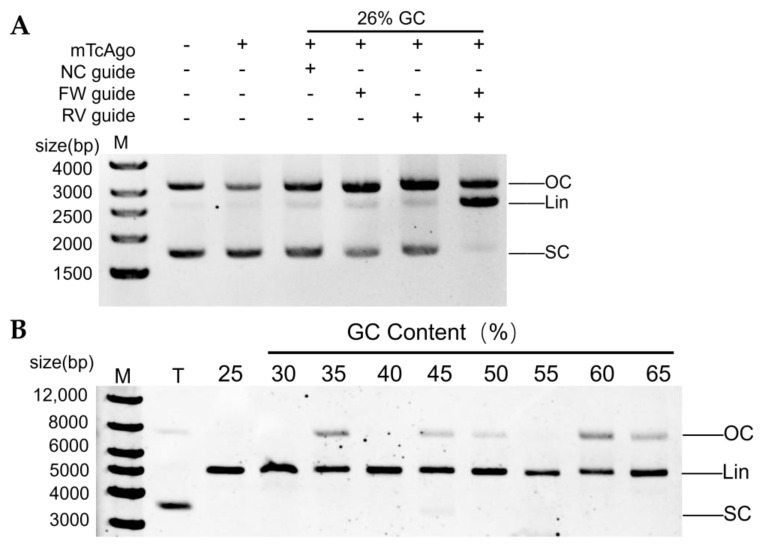
Cleavage of plasmid DNA by the mTcAgo-gDNA complex. (**A**): Cleavage of plasmid DNA by mTcAgo guided by 5′P-gDNA at 70 °C. (**B**): Analysis of the cleavage of plasmid target regions with varying GC content by mTcAgo at 80 °C. M indicates the 1 kb DNA ladder; T indicates plasmid DNA; NC indicates the negative control (non-complementary guide nucleic acid); FW, forward guide DNA; RV, reverse guide DNA; Lin indicates the linearized plasmid; SC indicates the supercoiled plasmid; and OC indicates the open circular plasmid. The original electrophoretic gels images can be found in Supplementary Materials.

Based on the efficient single-stranded DNA cleavage activity of mTcAgo guided by 5′P-gDNA, we further investigated its ability to cleave double-stranded DNA using pUC19 plasmid as a substrate. A pair of fully complementary forward and reverse gDNAs were designed to target a region with 26% GC content on the plasmid. After 10 min of cleavage at 70 °C, mTcAgo linearized the supercoiled pUC19 plasmid in the presence of both 5′P-gDNAs (Figure S7D). In the absence of gDNA or in the presence of an unrelated gDNA, no cleavage products were observed, and the plasmid remained predominantly supercoiled without obvious nicked or linearized bands. When only forward or reverse gDNA was added, a notable increase in the nicked form was observed, with only a small amount of linearized plasmid (Figure 8A).

We then examined the effects of Mg^2+^ and Mn^2+^ concentrations on plasmid DNA cleavage by mTcAgo. Unlike single-stranded target cleavage, mTcAgo required lower ion concentrations to cleave plasmid DNA. We hypothesize that the stable double-stranded helical conformation of plasmid DNA facilitates easier complex formation with gDNA, thereby requiring lower ion concentrations than for single-stranded nucleic acid cleavage. In the Mg^2+^ system, cleavage efficiency increased with rising ion concentration, with the best cleavage observed at 2–5 mM (Figure S7A). In contrast, higher Mn^2+^ concentrations led to lower cleavage efficiency, with the highest activity observed at 0.25 mM (Figure S7B). Furthermore, mTcAgo linearized supercoiled plasmid DNA within a temperature range of 75–92.5 °C. At temperatures below 80 °C, plasmid cleavage was incomplete, with some supercoiled plasmid remaining. At 92.5 °C, the cleavage activity of mTcAgo rapidly decreased (Figure S7C).

The cleavage efficiency of mTcAgo toward regions with different GC contents in plasmid DNA, particularly those with high GC content, directly reflects the universality and application potential of this Ago protein as an efficient cleavage tool. Therefore, under the optimized conditions described above, we assessed the cleavage efficiency of mTcAgo toward regions with varying GC contents in the pET28a vector plasmid at 80 °C. The results showed that mTcAgo could completely cleave supercoiled plasmids within regions of 25–65% GC content into linearized or nicked forms within a short time. Even for a region with 65% GC content, cleavage was nearly complete, with only a small amount of nicked plasmid remaining. Extended reaction time would likely achieve complete cleavage (Figure 8B).

## 4. Discussion

In previous studies, most Ago proteins adopt a conserved double-lobe structure, which plays an important role in guide nucleic acid recognition and target cleavage. Thermophilic pAgos maintain high activity and stability under high-temperature conditions, offering clear advantages in applications such as molecular diagnostics and large DNA fragment assembly. However, our understanding of the conformational changes that occur in thermophilic pAgo-nucleic acid complexes during catalysis remains limited, as does the structural information on how these changes affect catalytic activity. To further elucidate the recognition and cleavage mechanism of thermophilic pAgos from a structural perspective, this study employed cryo-electron microscopy to analyze the structure of TcAgo ternary complexes. Similar to PfAgo, TcAgo undergoes dimerization after binding to tDNA. The gDNA-tDNA duplexes were shorter overall, adopted an A-form conformation, and exhibited essentially identical base-pair pitches. Therefore, this target-induced dimerization mechanism may be conserved among thermophilic pAgos, representing a common feature of this protein family.

Previous studies have shown that cold-adapted enzymes often introduce glycine residues on the protein surface to increase flexibility and low-temperature activity through local or allosteric mechanisms [14]. Based on our cryo-EM structure of TcAgo, we therefore performed a rational cold-adaptation design by introducing glycine mutations at K574 and D577, generating the mTcAgo variant. This variant exhibited greatly enhanced catalytic activity and an expanded substrate spectrum at 37 °C. Our group has successfully engineered PfAgo and TtdAgo using the same glycine-substitution strategy. Unlike naturally mesophilic pAgos, which often have narrow guide preferences or strict cofactor requirements, our cold-adapted thermophilic variants retain high specificity and stability while gaining efficient activity at 37 °C. We hypothesize that these mutations enhance the conformational dynamics of the surface-exposed loop, thereby lowering the energy barrier for the structural adjustments required for TcAgo to function at 37 °C. In the future, we will pursue cryo-EM structure determination of thermophilic prokaryotic Argonaute proteins (including TcAgo, PfAgo, and TtdAgo) and their mutants at different temperatures. We expect to utilize these structural data to analyze the possible structural reasons for the effects of residues located on surface-exposed loops on the activity of thermophilic prokaryotic Argonaute proteins. Based on these findings, we hypothesize that this cold-adaptation strategy could be generalized to other thermophilic pAgos, provided that equivalent surface-exposed loop positions can be identified from high-resolution structures.

The efficient nuclease activity and specific cleavage of target DNA and target RNA by mTcAgo at room temperature in vitro also demonstrate its application potential for in vivo genome editing and the development of RNA-targeting tools. In the future, since targeting RNA does not involve the constraints of supercoiling, we can first explore the potential of mTcAgo in RNA targeting of mammalian cells, systematically investigating its targeting efficiency, targeting specificity, sequence requirements for targeting, effective transfection methods, and so on. Furthermore, for in vivo gene editing in mammalian cells, we can attempt to achieve this by fusing mTcAgo with helicase domains, other nuclease domains, or the like. In summary, mTcAgo provides an alternative and novel nucleic acid manipulation tool.

## 5. Conclusions

Here, we resolved the ternary complex structure of TcAgo, guide DNA, and target DNA, and successfully obtained a better-performing mutant, mTcAgo, by applying cold adaptation engineering based on its cryo-EM structure and characteristics of cold-adapted enzymes. The mutant assumed a wider catalytic temperature range and a broader substrate spectrum, acquiring DNA and RNA cleavage activity at 37 °C relative to its wild type. Apart from that, mTcAgo boosted its catalytic activity at both mesophilic and high temperatures while maintaining high thermostability, vastly expanding its application scope.

In vitro characterization unraveled that mTcAgo cleaved complementary DNA using 5′OH-/5′P-gDNA or 5′OH-/5′P-gRNA, and complementary RNA using 5′OH-/5′P-gDNA at 37 °C. Its reaction system was simple, with minimal salt effects and optimal pH 7–8; notably, mTcAgo denatured near its isoelectric point. For 5′P-gDNA-guided cleavage, 5–10 mM Mn^2+^ and a 17 nt guide were optimal; the lowest efficiency occurred with 5′-G, and mismatches were well tolerated. For 5′OH-gDNA-guided tDNA cleavage, 0.5–5 mM Mn^2+^ and an 18 nt guide were optimal, with no 5′-end preference but severe impairment by seed mismatches. Thermal stability tests showed that after pre-incubation at 90 °C for 20 min, mTcAgo retained DNA and RNA cleavage activity at 37 °C; DSF gave a Tm of 88 °C. Kinetic analysis at 37 °C indicated that the fastest rate was achieved when targeting RNA with 5′P-gDNA. At elevated temperatures, mTcAgo efficiently cleaved plasmid DNA with as low as 0.25 mM Mn^2+^ or 1.5 mM Mg^2+^ (80–90 °C optimal), completely cleaving 200 ng within 10 min, and specifically targeted regions with up to 65% GC content.

## Figures and Tables

**Table 1:** Primers used in this study.

Gene	Primer	Sequence (5′-3′)
K574G	K574G-F	TTGTCCGGGTCCACTGACTACGACCT
	K574G-R	AGTGGACCCGGACAAAGTCAAGTACTTCACACCC
S575G	S575G-F	TCCAAGGGGACTGACTACGACCTGATCATCGG
	S575G-R	GTCAGTCCCCTTGGACAAAGTCAAGTACTTCACACCCAG
D577G	D577G-F	TCCACTGGGTACGACCTGATCATCGGTGTTGATGT
	D577G-R	GTCGTACCCAGTGGACTTGGACAAAGTCAAGTACTTCACAC
K644G	K644G-F	GAGTTGGGGGGTAAGAGAATCCTGGTCTTGC
	K644G-R	CTTACCCCCCAACTCAGCGTAGTTCTCGTACTCG
R669G	R669G-F	ATCTCCGGGACTAAGGACTGTACCATCGAGG
	R669G-R	CTTAGTCCCGGAGATGTCGGCCAACTGAGTAAC
T670G	T670G-F	TCCAGAGGGAAGGACTGTACCATCGAGGTCATCAA
	T670G-R	GTCCTTCCCTCTGGAGATGTCGGCCAACTGAGTAACC
D672G	D672G-F	ACTAAGGGGTGTACCATCGAGGTCATCAACGTCAAG
	D672G-R	GGTACACCCCTTAGTTCTGGAGATGTCGGCCAACT
K731G	K731G-F	TTCGCCGGGGGTACTGTCAGAGAAGAAAGA
	K731G-R	AGTACCCCCGGCGAACACGTACTTCACCTTGT
mTcAgo-DM	Tc-D585A-F	CGGTGTTGCCGTTGGTCACGGTGAGGTTGA
	Tc-D585A-R	CCAACGGCAACACCGATGATCAGGTCGTA
	Tc-D653A-F	CTTGCGTGCCGGTAGACTGACCAAAGAAGAGGTTAC
	Tc-D653A-R	CTACCGGCACGCAAGACCAGGATTCTCT

**Table 2:** Oligonucleotides used in complex assembly.

Oligonucleotide Name	Sequence (5′-3′)
16 nt guide DNA	5′P-TGAGGTAGTAGGTTGT-3′
17 nt target DNA	5′-ACAACCTACTACCTCAT-3′

**Table 3:** Nucleic acid sequences for plasmid cleavage experiments.

Oligonucleotide Name	Sequence (5′-3′)
26%-gDNA-F	CATGAGCGGATACATATT
26%-gDNA-R	CAAATATGTATCCGCTCA
25%-gDNA-F	AAATGAAACTGCAATTTA
25%-gDNA-R	TAAATTGCAGTTTCATTT
30%-gDNA-F	GCAATTTATTCATATCAG
30%-gDNA-R	CTGATATGAATAAATTGC
36%-gDNA-F	GAGTGACGACTGAATCCG
36%-gDNA-R	CGGATTCAGTCGTCACTC
40%-gDNA-F	AAGGTTATCAAGTGAGAA
40%-gDNA-R	TTCTCACTTGATAACCTT
45%-gDNA-F	CTGTTGATGGGTGTCTGG
45%-gDNA-R	CCAGACACCCATCAACAG
50%-gDNA-F	CTGGTATCGGTCTGCGAT
50%-gDNA-R	ATCGCAGACCGATACCAG
55%-gDNA-F	CCTAATGAGTGAGCTAAC
55%-gDNA-R	GTTAGCTCACTCATTAGG
60%-gDNA-F	GGCTCTCAAGGGCATCGG
60%-gDNA-R	CCGATGCCCTTGAGAGCC
65%-gDNA-F	GTCCCCCGGCCACGGGGC
65%-gDNA-R	GCCCCGTGGCCGGGGGAC

All percentage values in this table represent the GC content of the targeted region on the plasmid DNA, not the guide sequences.

## Data Availability

All relevant data of this study are presented. Additional data will be provided upon request. Cryo-EM reconstructions of TcAgo-gDNA-tDNA have been deposited in the Electron Microscopy Data Bank under the accession numbers EMD-38241. Coordinates for atomic models of TcAgo-guide DNA-target DNA have been deposited in the Protein Data Bank under the accession numbers 8XCF.

## Supplementary Materials

The following supporting information can be downloaded at: https://www.mdpi.com/article/10.3390/biom16050715/s1. Figure S1. Cryo-EM of TcAgo-guide DNA-target DNA complex. (A): Raw cryo-EM images of TcAgo. (B): Representative 2D class averages. (C): 3D classification with particle distribution. (D): Final reconstruction. (E): Angular distribution of the reconstruction shown in (D). (F): Local resolution estimation on the reconstruction in (D). (G): Global half-map FSC plot of the reconstruction shown in (D). Figure S2. Cleavage activity assay of TcAgo. (A): The gel diagram showing target DNA and RNA cleavage activity of TcAgo using different guide nucleic acids. (B): The gel diagram showing TcAgo cleavage activity at different temperatures. Figure S3. Cleavage activity assay of mTcAgo with different buffer conditions at 37 °C. (A): The gel diagram showing the activity of mTcAgo at 37 °C under different pH. (B): The gel diagram showing the activity of mTcAgo at 37 °C under different NaCl concentrations. (C): The gel diagram showing the cleavage activity of mTcAgo at 37 °C in the presence of different divalent metal ions. (D): The gel diagram showing mPfAgo activity at 37 °C at different Co^2+^ or Mn^2+^ concentrations. Figure S4. Cleavage activity assay of mTcAgo with varied guide lengths and sequences at 37 °C. (A): The gel diagram showing mTcAgo activity at 37 °C using guides of varied lengths. (B): The gel diagram showing mTcAgo activity at 37 °C using guides with different 5′-end nucleotides. Figure S5. The gel diagram showing mTcAgo activity at 37 °C with different guide-target mismatch sites. Figure S6. Assay of cleavage activity of mTcAgo at different times and temperatures. (A): The gel diagram showing mTcAgo cleavage activity at different gDNA loaded temperatures. (B): The gel diagram showing mTcAgo cleavage activity at different temperatures. (C): The gel diagram showing the time-course of target cleavage at 37 °C by mTcAgo. Figure S7. Cleavage of plasmid DNA by the mTcAgo. Examination of Mg^2+^ (A), and Mn^2+^(B) concentration effects on pUC19 plasmid cleavage. (C): Effects of temperature on pUC19 plasmid cleavage by mTcAgo. (D): Effect of time on pUC19 plasmid cleavage by mTcAgo. Table S1. The nucleic sequences used in ssDNA and RNA cleavage. The original electrophoretic gels images can be found in Supplementary Materials.
