# Supplementary material for: Structure-Based Rational Design of TcAgo from Thermogladius calderae
Source: Biomolecules. 2026 May 13;16(5):715. doi: 10.3390/biom16050715 (PMC13204829; doi:10.3390/biom16050715)
Supplement: Supplementary file 1 [file biomolecules-16-00715-s001.zip › biomolecules-4288869-supplementary.pdf]

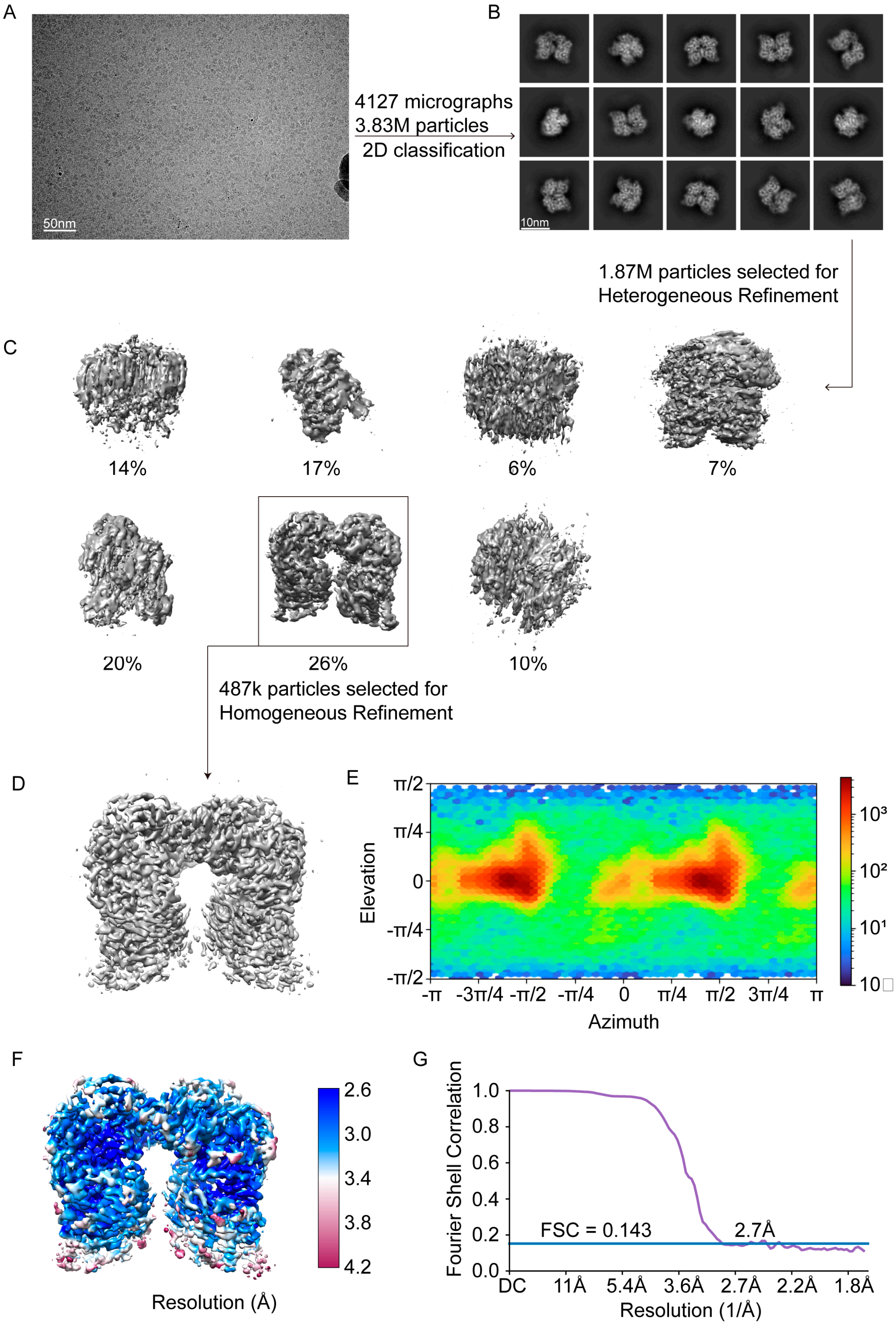

Figure S1. Cryo-EM of TcAgo-guide DNA-target DNA complex. (A): Raw cryo-EM images of TcAgo. (B): Representative 2D class averages. (C): 3D classification with particle distribution. (D): Final reconstruction. (E): Angular distribution of the reconstruction shown in D.(F): Local resolution estimation on the reconstruction in D. (G): Global half-map FSC plot of the reconstruction shown in D.

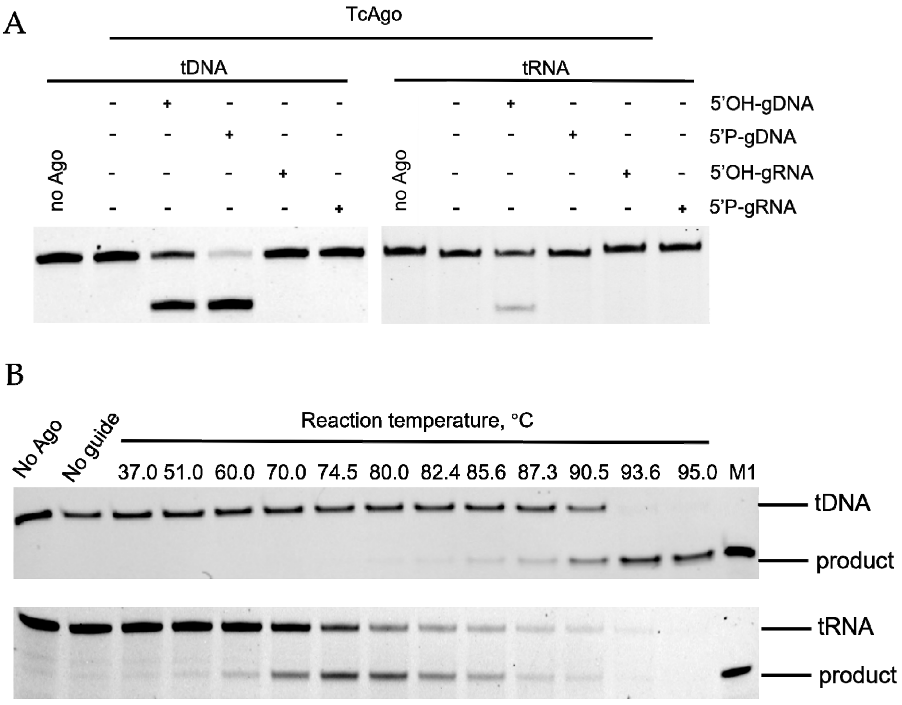

Figure S2. Cleavage activity assay of TcAgo. (A): The gel diagram showing target DNA and RNA cleavage activity of TcAgo using different guide nucleic acids. (B): The gel diagram showing TcAgo cleavage activity at different temperatures.

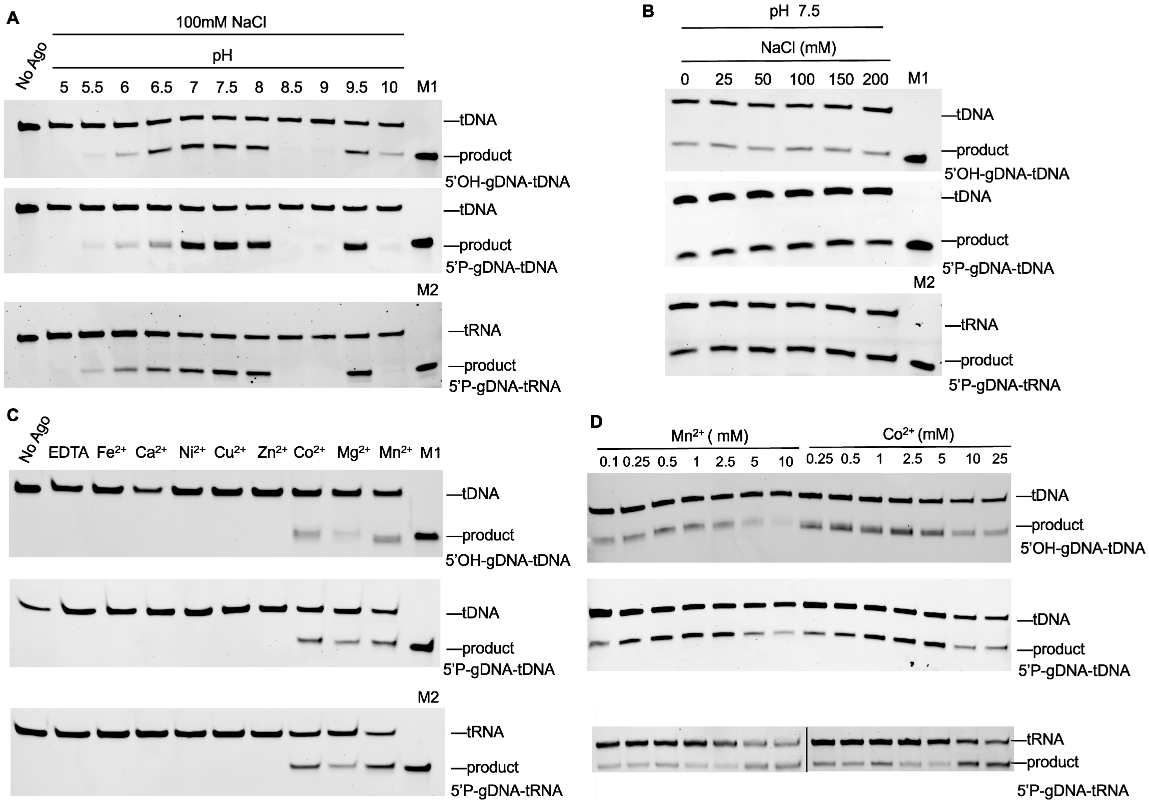

Figure S3. Cleavage activity assay of mTcAgo with different buffer conditions at 37 °C. (A): The gel

diagram showing the activity of mTcAgo at 37 °C under different pH. (B): The gel diagram showing the activity of mTcAgo at 37 °C under different NaCl concentrations. (C): The gel diagram showing the cleavage activity of mTcAgo at 37 °C in the presence of different divalent metal ions. (D): The gel diagram showing mPfAgo activity at 37 °C at different Co^{2+} or Mn^{2+} concentrations.

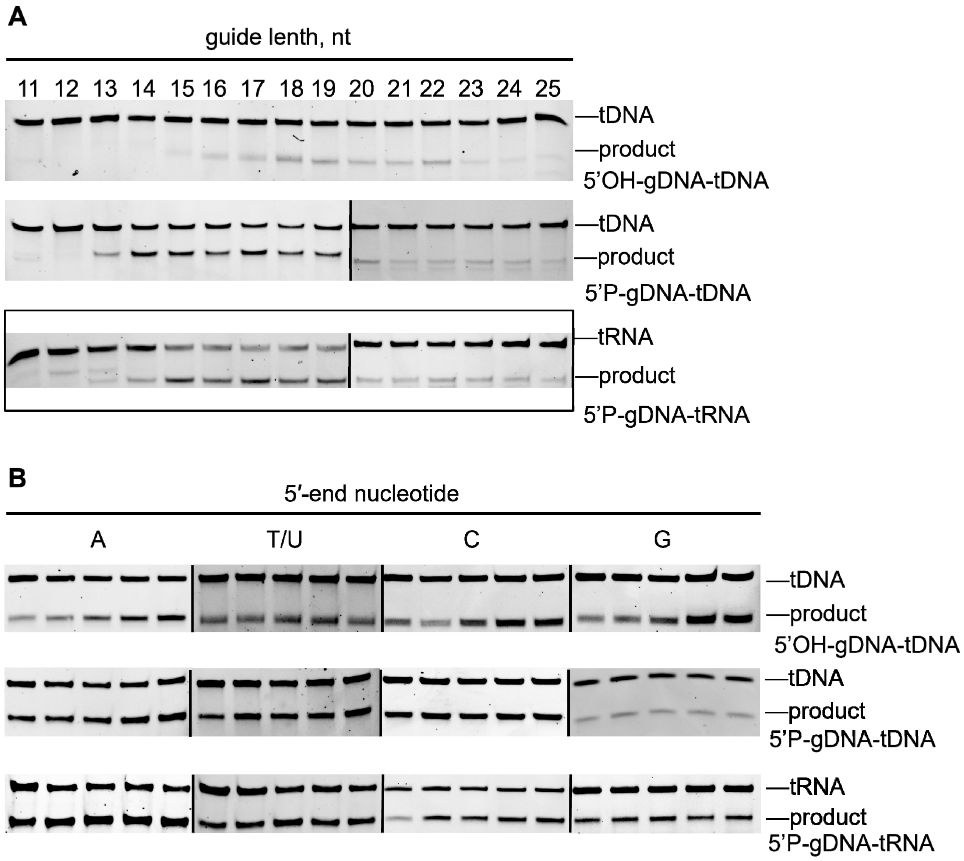

Figure S4. Cleavage activity assay of mTcAgo with varied guide lengths and sequences at 37 °C. (A): The gel diagram showing mTcAgo activity at 37 °C using guides of varied lengths. (B): The gel diagram showing mTcAgo activity at 37 °C using guides with different 5′-end nucleotides.

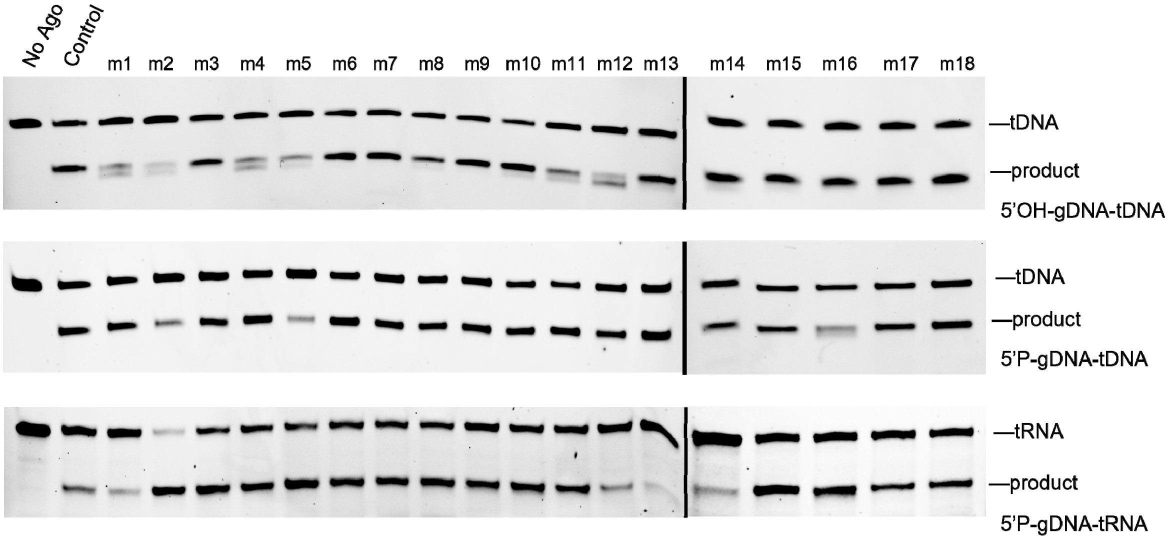

Figure S5. The gel diagram showing mTcAgo activity at 37 °C with different guide-target mismatch sites.

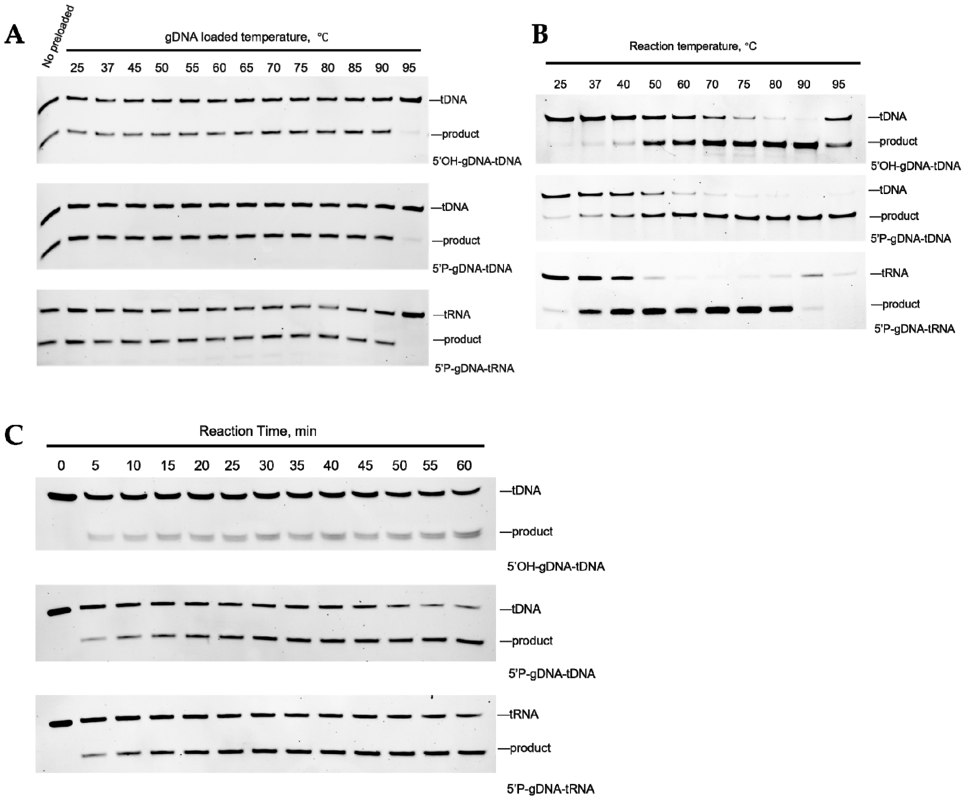

Figure S6. Assay of cleavage activity of mTcAgo at different times and temperatures. (A): The gel diagram showing mTcAgo cleavage activity at different gDNA loaded temperatures. (B): The gel diagram showing mTcAgo cleavage activity at different temperatures. (C): The gel diagram showing the time-course of target cleavage at 37 °C by mTcAgo.

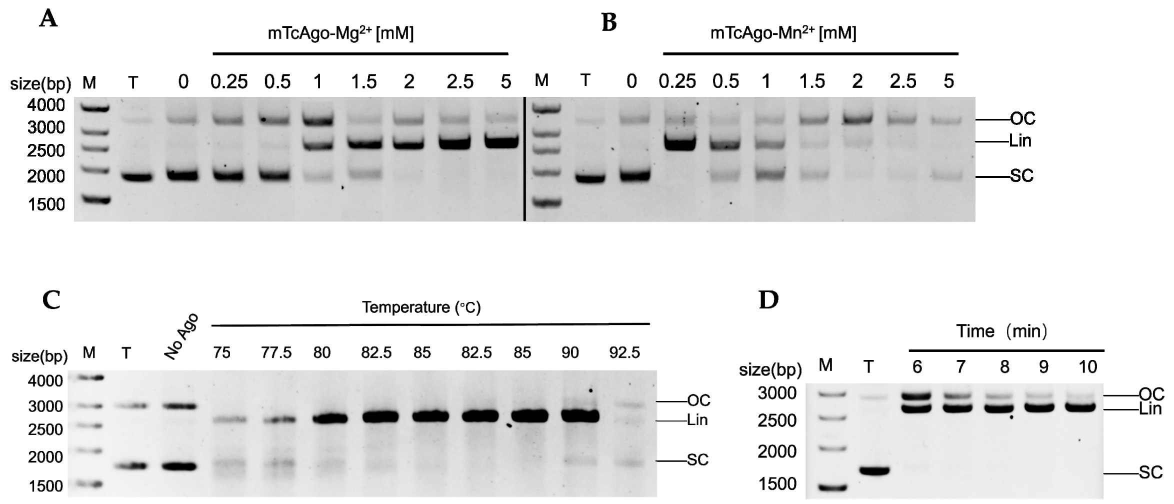

Figure S7. Cleavage of plasmid DNA by the mTcAgo. Examination of Mg^{2+} (A), and Mn^{2+}(B) concentration effects on pUC19 plasmid cleavage. (C): Effects of temperature on pUC19 plasmid cleavage by mTcAgo. (D): Effect of time on pUC19 plasmid cleavage by mTcAgo.

Table S1 The nucleic sequences used in ssDNA and RNA cleavage

Oligonucleotide name	Sequence (5′-3′)	Description
FAM-A-tDNA	FAM-AAACGACGGCCAGTGCCAAG CTTACTATACAACCTACTACCTCTT	5′FAM labeled A-tDNA
FAM-T-tDNA	FAM-AAACGACGGCCAGTGCCAAG CTTACTATACAACCTACTACCTCAT	5′FAM labeled T-tDNA
FAM-G-tDNA	FAM-AAACGACGGCCAGTGCCAAG CTTACTATACAACCTACTACCTCCT	5′FAM labeled G-tDNA
FAM-C-tDNA	FAM-AAACGACGGCCAGTGCCAAG CTTACTATACAACCTACTACCTCGT	5′FAM labeled C-tDNA
FAM-A-tRNA	FAM-AAACGACGGCCAGUGCCAAG CUUACUAUACAACCUACUACCUCUU	5′FAM labeled A-tRNA
FAM-U-tRNA	FAM-AAACGACGGCCAGUGCCAAG CUUACUAUACAACCUACUACCUCAU	5′FAM labeled T-tRNA
FAM-G-tRNA	FAM-AAACGACGGCCAGUGCCAAG CUUACUAUACAACCUACUACCUCCU	5′FAM labeled G-tRNA
FAM-C-tRNA	FAM-AAACGACGGCCAGUGCCAAG CUUACUAUACAACCUACUACCUCGU	5′FAM labeled C-tRNA
FAM-M1	FAM-AAACGACGGCCAGTGCCAAG	5′FAM labeled 34nt DNA

	CTTACTATACAACC	
FAM-M2	FAM-AAACGACGGCCAGUGCCAAG CUUACUAUACAACC	5′FAM labeled 34nt RNA
A-gDNA	AGAGGTAGTAGGTTGTAT	DNA guide forms 5'-A pair with A-tDNA/A-tRNA
T-gDNA	TGAGGTAGTAGGTTGTAT	DNA guide forms 5'-T pair with T-tDNA/U-tRNA
G-gDNA	GGAGGTAGTAGGTTGTAT	DNA guide forms 5'-G pair with G-tDNA/G-tRNA
C-gDNA	CGAGGTAGTAGGTTGTAT	DNA guide forms 5'-C pair with C-tDNA/C-tRNA
5′P-A-gDNA	5′P-AGAGGTAGTAGGTTGTAT	5'phosphorylated A-gDNA
5′P-T-gDNA	5′P-TGAGGTAGTAGGTTGTAT	5'phosphorylated T-gDNA
5′P-G-gDNA	5′P-GGAGGTAGTAGGTTGTAT	5'phosphorylated G-gDNA
5′P-C-gDNA	5′P-CGAGGTAGTAGGTTGTAT	5'phosphorylated C-gDNA
11 nt-gDNA	TGAGGTAGTAG	11 nt DNA guide pair with T-tDNA/U-tRNA
12 nt-gDNA	TGAGGTAGTAGG	12 nt DNA guide pair with T-tDNA/U-tRNA
13 nt-gDNA	TGAGGTAGTAGGT	13 nt DNA guide pair with T-tDNA/U-tRNA
14 nt-gDNA	TGAGGTAGTAGGTT	14 nt DNA guide pair with T-tDNA/U-tRNA
15 nt-gDNA	TGAGGTAGTAGGTTG	15 nt DNA guide pair with T-tDNA/U-tRNA
16 nt-gDNA	TGAGGTAGTAGGTTGT	16 nt DNA guide pair with T-tDNA/U-tRNA
17 nt-gDNA	TGAGGTAGTAGGTTGTA	17 nt DNA guide pair with T-tDNA/U-tRNA
18 nt-gDNA	TGAGGTAGTAGGTTGTAT	18 nt DNA guide pair with T-tDNA/U-tRNA
19 nt-gDNA	TGAGGTAGTAGGTTGTATA	19 nt DNA guide pair with T-tDNA/U-tRNA
20 nt-gDNA	TGAGGTAGTAGGTTGTATAG	20 nt DNA guide pair with T-tDNA/U-tRNA
21 nt-gDNA	TGAGGTAGTAGGTTGTATAGT	21 nt DNA guide pair with

		T-tDNA/U-tRNA
22 nt-gDNA	TGAGGTAGTAGGTTGTATAGTA	22 nt DNA guide pair with T-tDNA/U-tRNA
23 nt-gDNA	TGAGGTAGTAGGTTGTATAGTAA	23 nt DNA guide pair with T-tDNA/U-tRNA
24 nt-gDNA	TGAGGTAGTAGGTTGTATAGTAAG	24 nt DNA guide pair with T-tDNA/U-tRNA
25 nt-gDNA	TGAGGTAGTAGGTTGTATAGTAAGC	25 nt DNA guide pair with T-tDNA/U-tRNA
gDNA-m1	AGAGGTAGTAGGTTGTATAGTAAGC	DNA guide forms mismatched pair in position 1 with tDNA/tRNA
gDNA-m2	TCAGGTAGTAGGTTGTATAGTAAGC	DNA guide forms mismatched pair in position 2 with tDNA/tRNA
gDNA-m3	TCTGGTAGTAGGTTGTATAGTAAGC	DNA guide forms mismatched pair in position 3 with tDNA/tRNA
gDNA-m4	TGACGTAGTAGGTTGTATAGTAAGC	DNA guide forms mismatched pair in position 4 with tDNA/tRNA
gDNA-m5	TGAGCTAGTAGGTTGTATAGTAAGC	DNA guide forms mismatched pair in position 5 with tDNA/tRNA
gDNA-m6	TGAGGAAGTAGGTTGTATAGTAAGC	DNA guide forms mismatched pair in position 6 with tDNA/tRNA
gDNA-m7	TGAGGTTGTAGGTTGTATAGTAAGC	DNA guide forms mismatched pair in position 7 with tDNA/tRNA
gDNA-m8	TGAGGTACTAGGTTGTATAGTAAGC	DNA guide forms mismatched pair in position 8 with tDNA/tRNA
gDNA-m9	TGAGGTAGAAGGTTGTATAGTAAGC	DNA guide forms mismatched pair in position 9 with tDNA/tRNA
gDNA-m10	TGAGGTAGTTGGTTGTATAGTAAGC	DNA guide forms mismatched

		pair in position 10 with tDNA/tRNA
gDNA-m11	TGAGGTAGTACGTTGTATAGTAAGC	DNA guide forms mismatched pair in position 11 with tDNA/tRNA
gDNA-m12	TGAGGTAGTAGCTTGTATAGTAAGC	DNA guide forms mismatched pair in position 12 with tDNA/tRNA
gDNA-m13	TGAGGTAGTAGGATGTATAGTAAGC	DNA guide forms mismatched pair in position 13 with tDNA/tRNA
gDNA-m14	TGAGGTAGTAGGTAGTATAGTAAGC	DNA guide forms mismatched pair in position 14 with tDNA/tRNA
gDNA-m15	TGAGGTAGTAGGTTCTATAGTAAGC	DNA guide forms mismatched pair in position 15 with tDNA/tRNA
gDNA-m16	TGAGGTAGTAGGTTGAATAGTAAGC	DNA guide forms mismatched pair in position 16 with tDNA/tRNA
gDNA-m17	TGAGGTAGTAGGTTGTTTAGTAAGC	DNA guide forms mismatched pair in position 17 with tDNA/tRNA
gDNA-m18	TGAGGTAGTAGGTTGTAAAGTAAGC	DNA guide forms mismatched pair in position 18 with tDNA/tRNA
