# Supplementary figures and images for: Structure-Based Rational Design of TcAgo from Thermogladius calderae
Source: Biomolecules. 2026 May 13;16(5):715. doi: 10.3390/biom16050715 (PMC13204829; doi:10.3390/biom16050715)

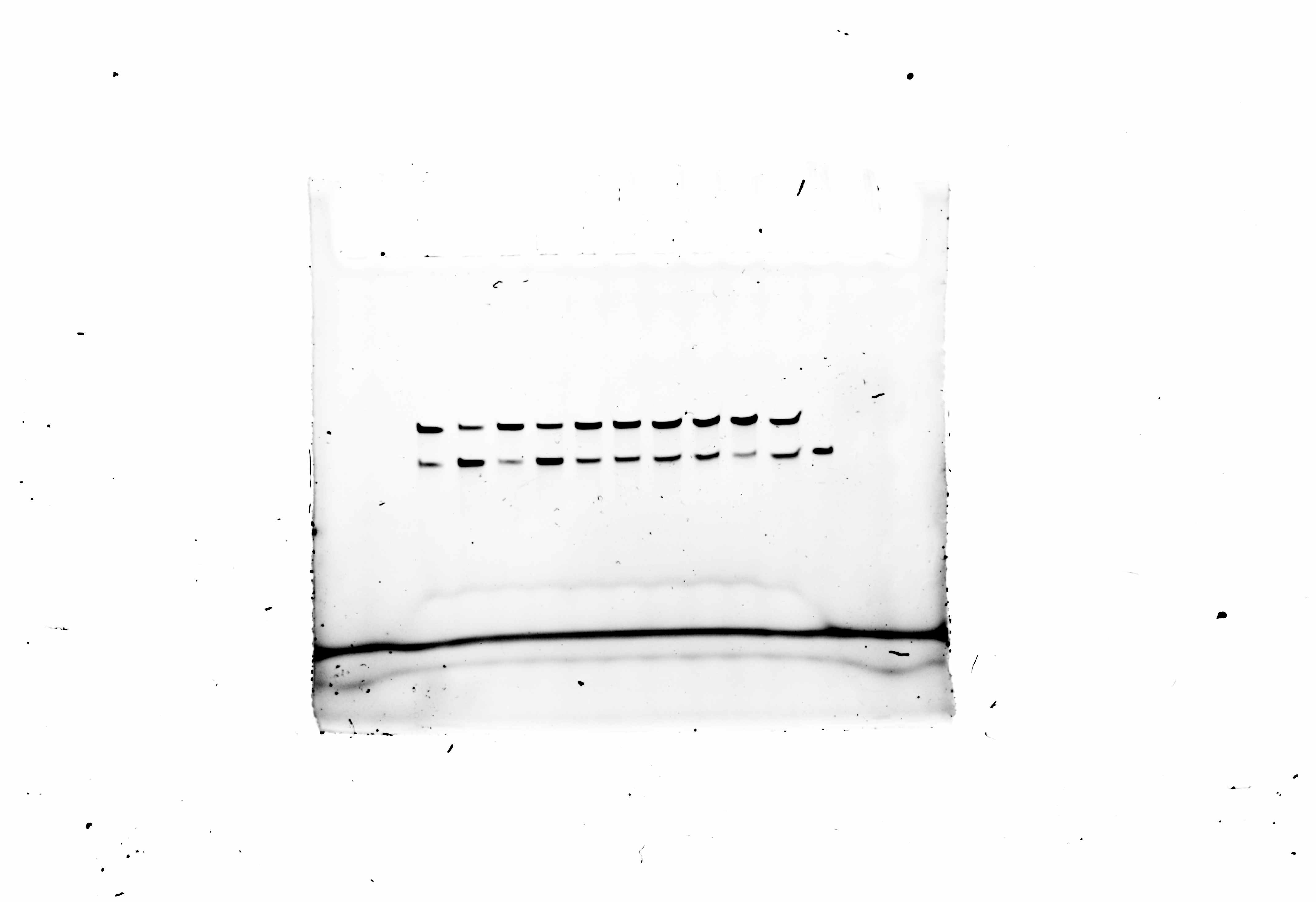

Supplement: Supplementary file 1 [file biomolecules-16-00715-s001.zip › Original-Images/Fig2C.tif]

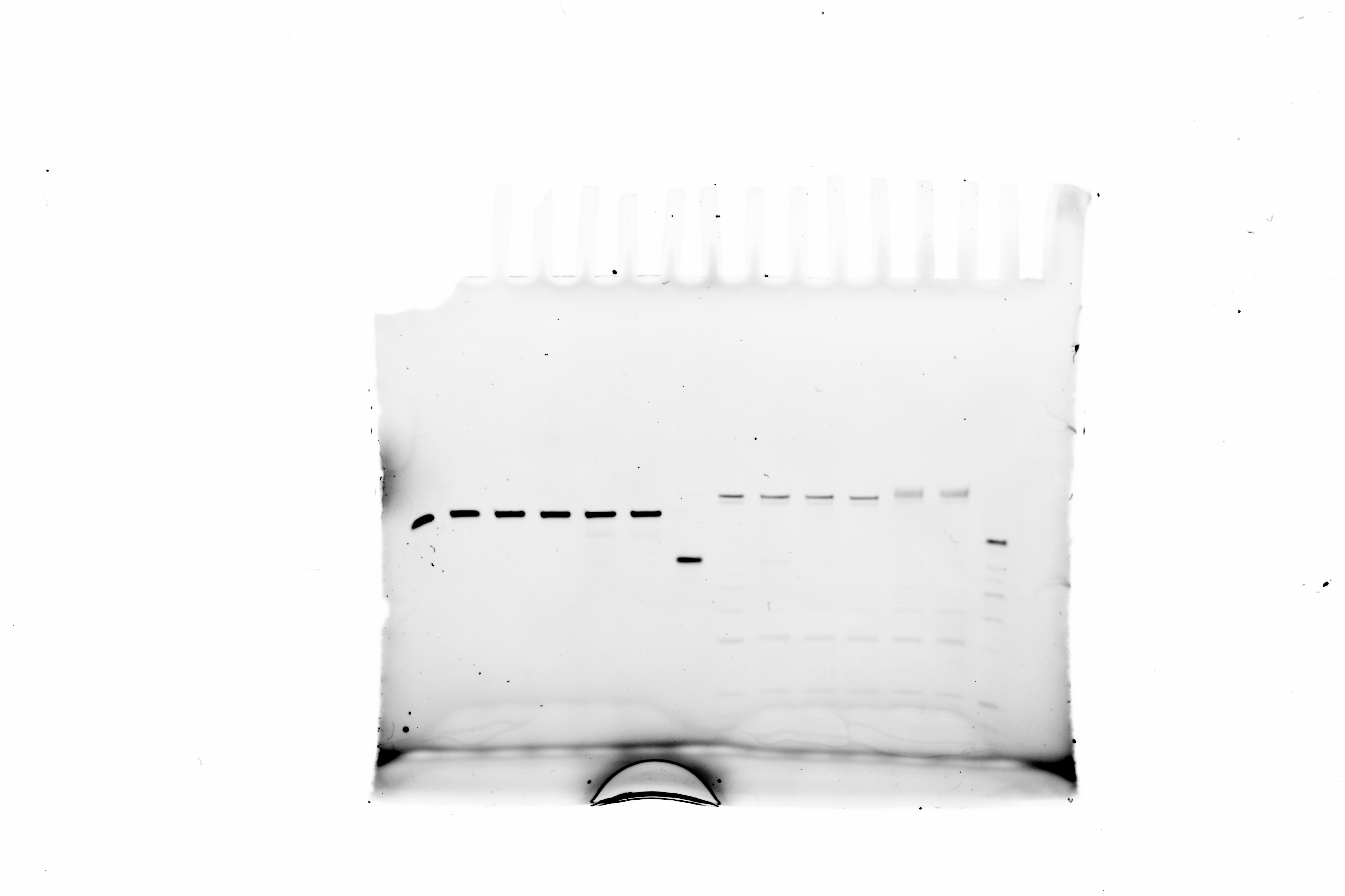

Supplement: Supplementary file 1 [file biomolecules-16-00715-s001.zip › Original-Images/Fig3B-1.tif]

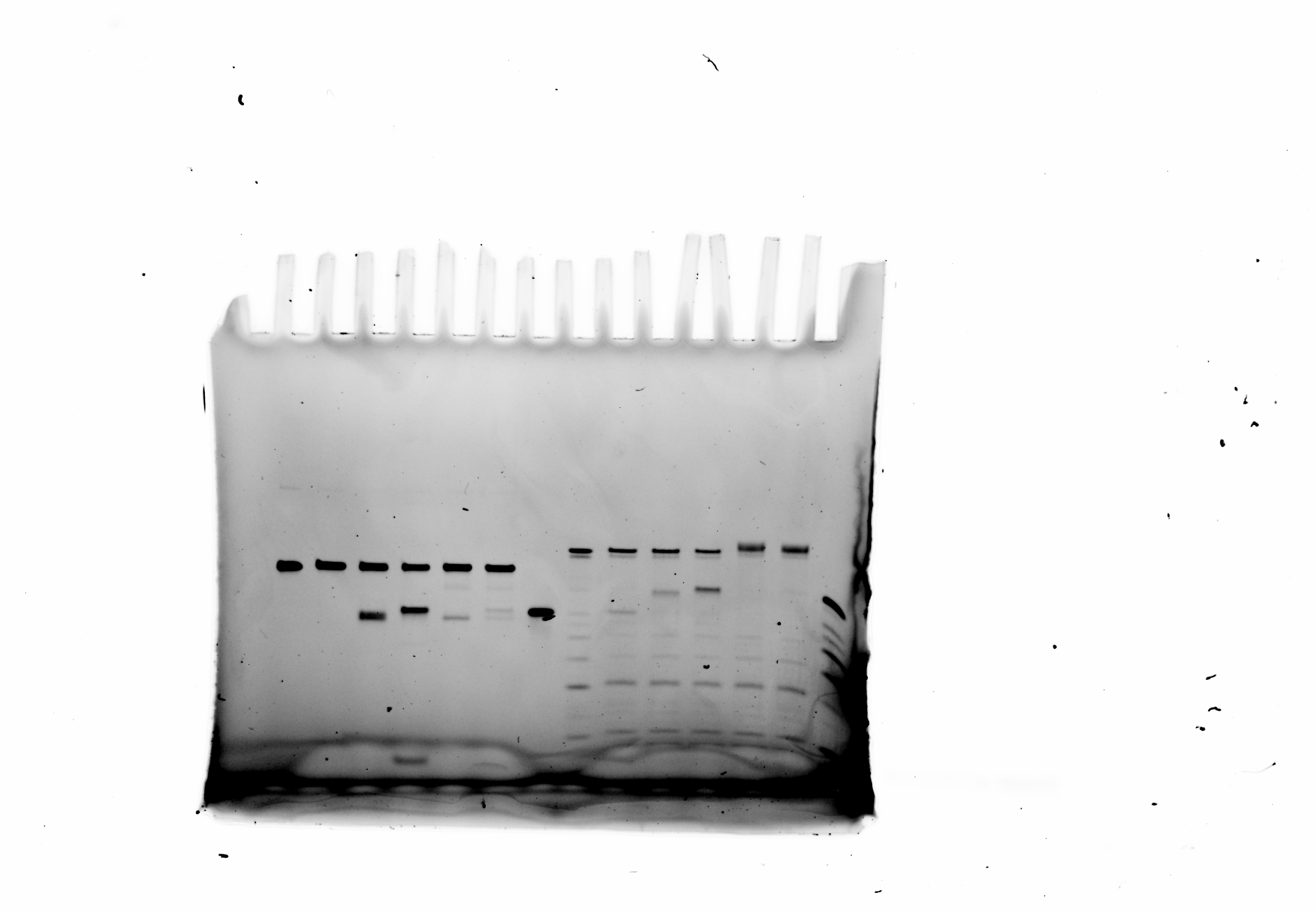

Supplement: Supplementary file 1 [file biomolecules-16-00715-s001.zip › Original-Images/Fig3B-2.tif]

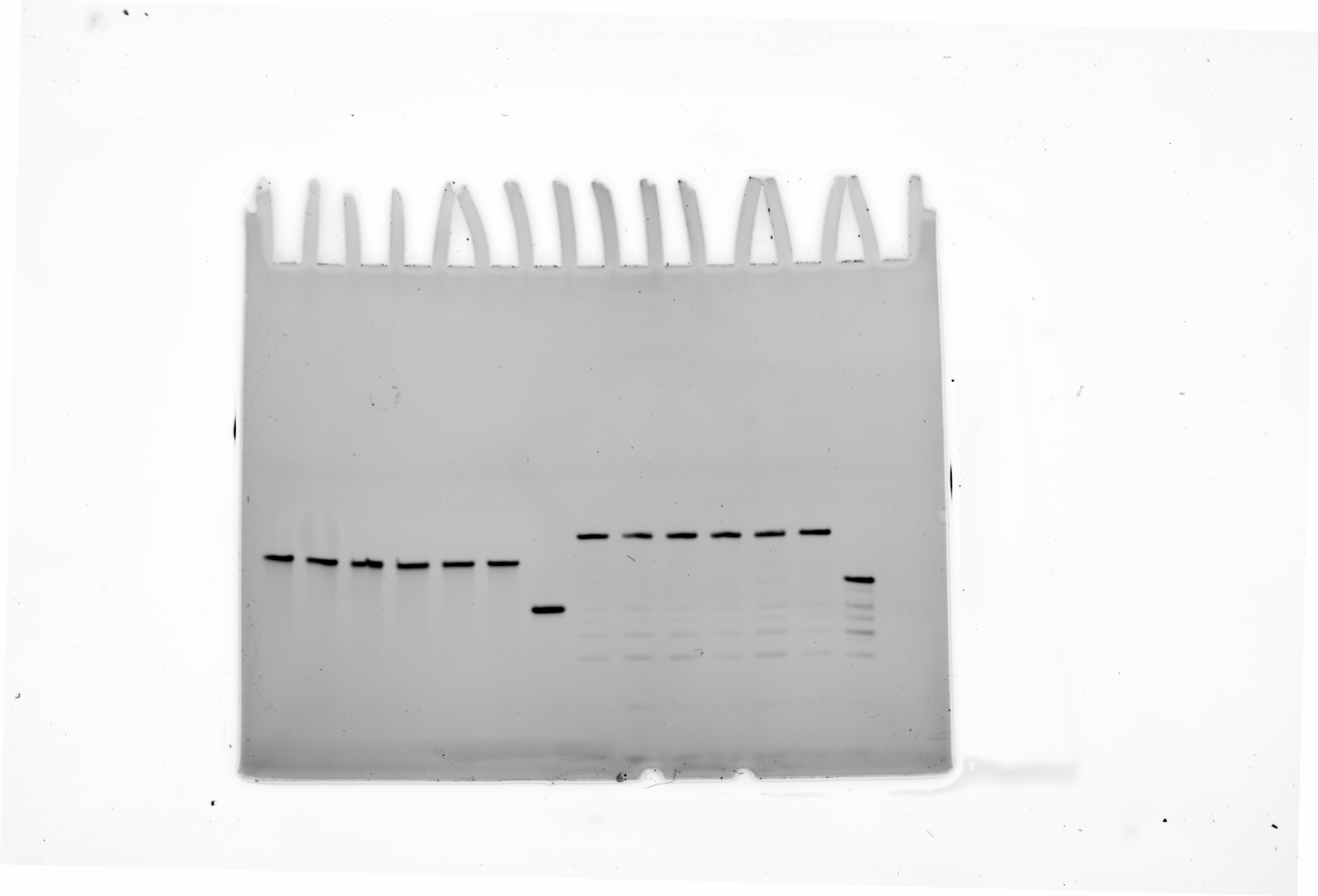

Supplement: Supplementary file 1 [file biomolecules-16-00715-s001.zip › Original-Images/Fig3B-3.tif]

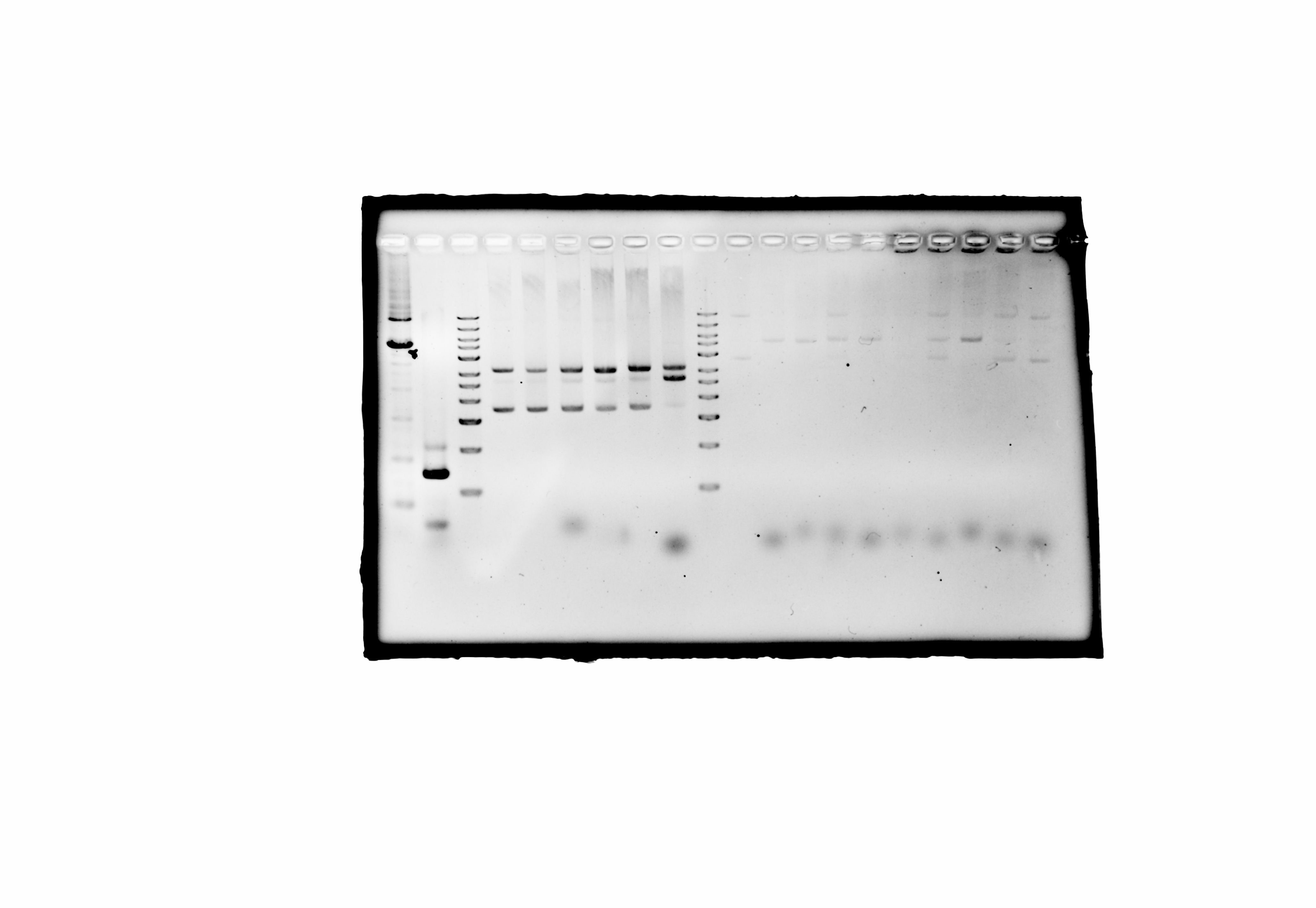

Supplement: Supplementary file 1 [file biomolecules-16-00715-s001.zip › Original-Images/Fig8A.tif]

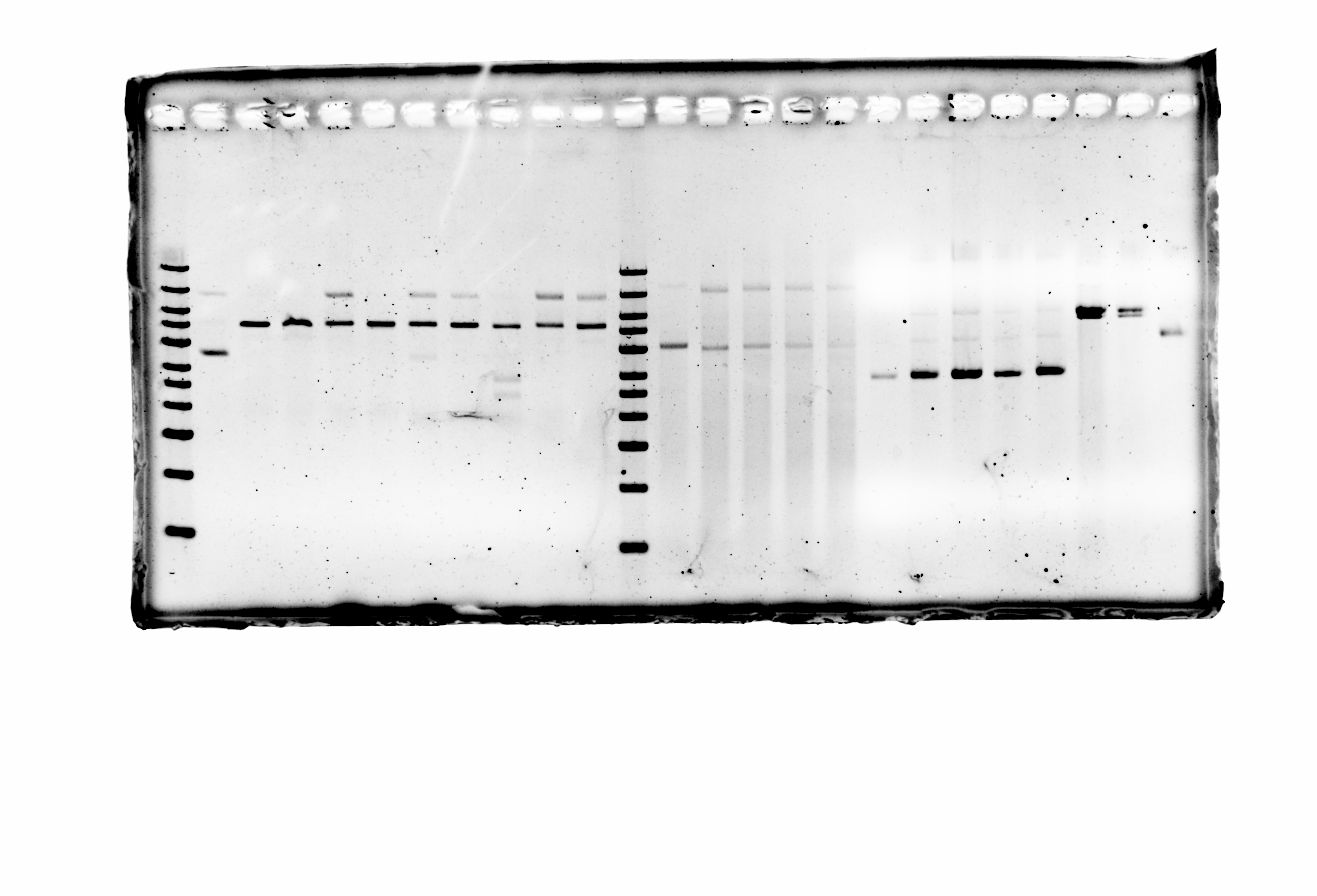

Supplement: Supplementary file 1 [file biomolecules-16-00715-s001.zip › Original-Images/Fig8B.tif]

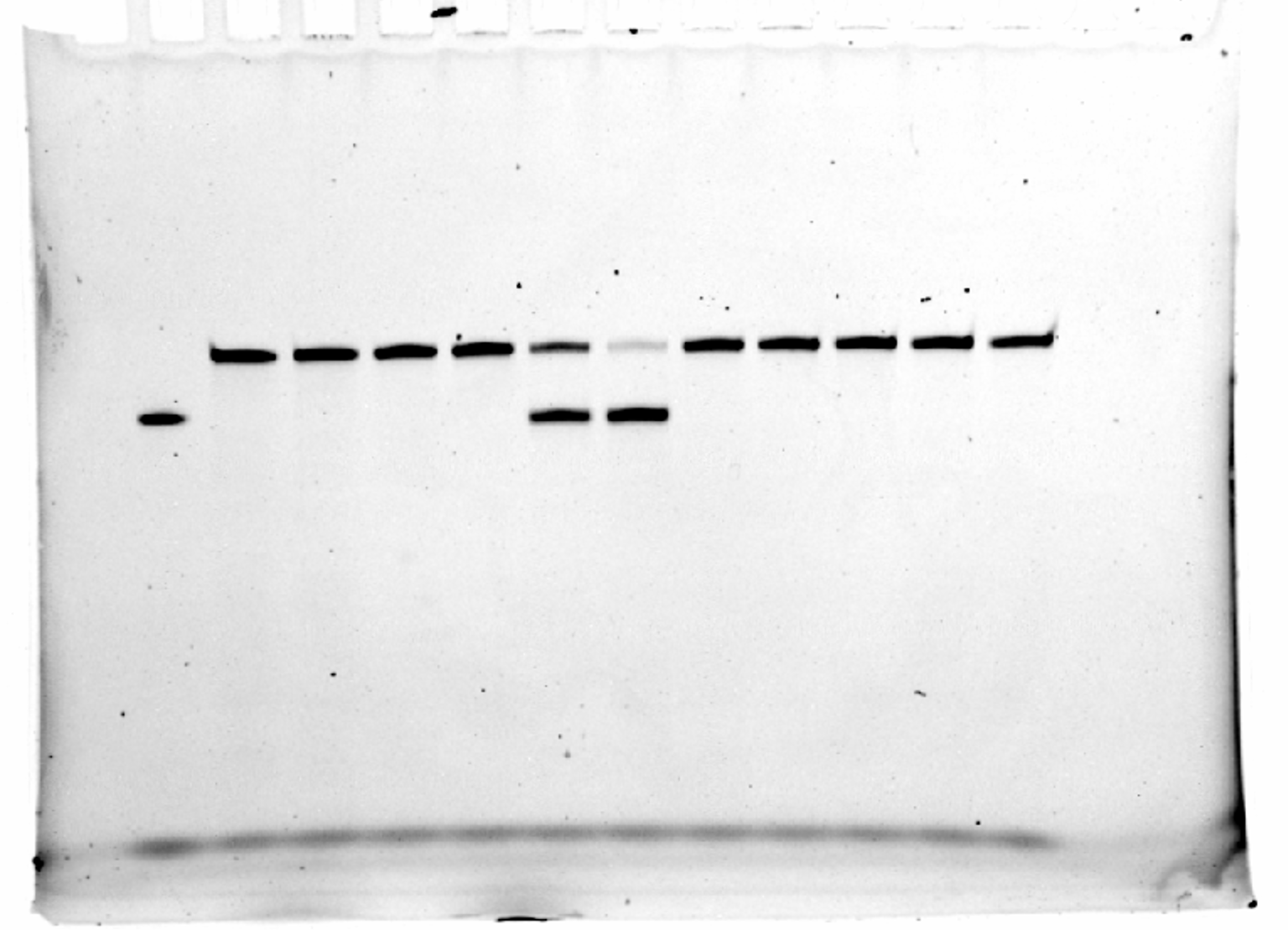

Supplement: Supplementary file 1 [file biomolecules-16-00715-s001.zip › Original-Images/FigS2A-1.tif]

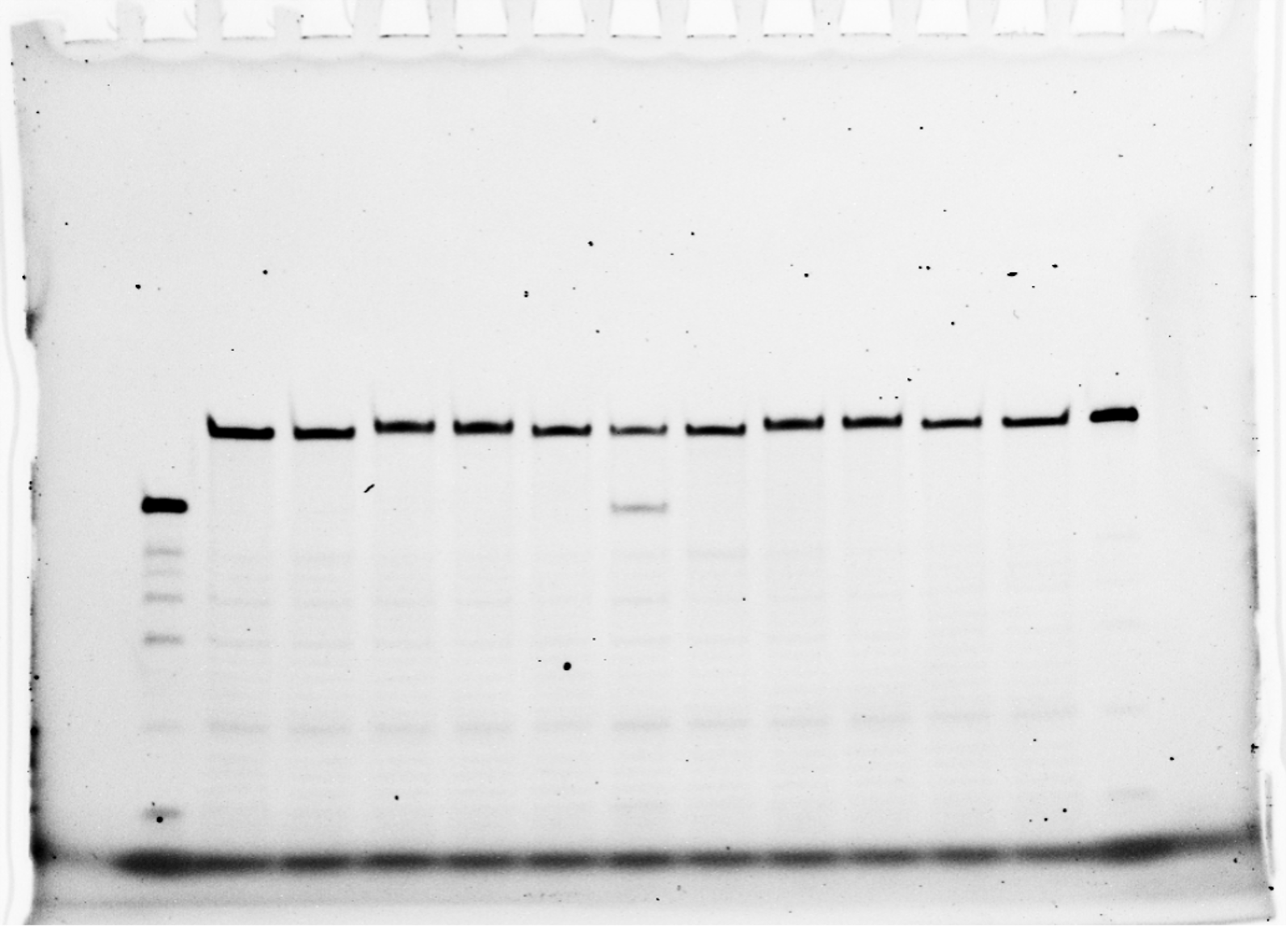

Supplement: Supplementary file 1 [file biomolecules-16-00715-s001.zip › Original-Images/FigS2A-2.tif]

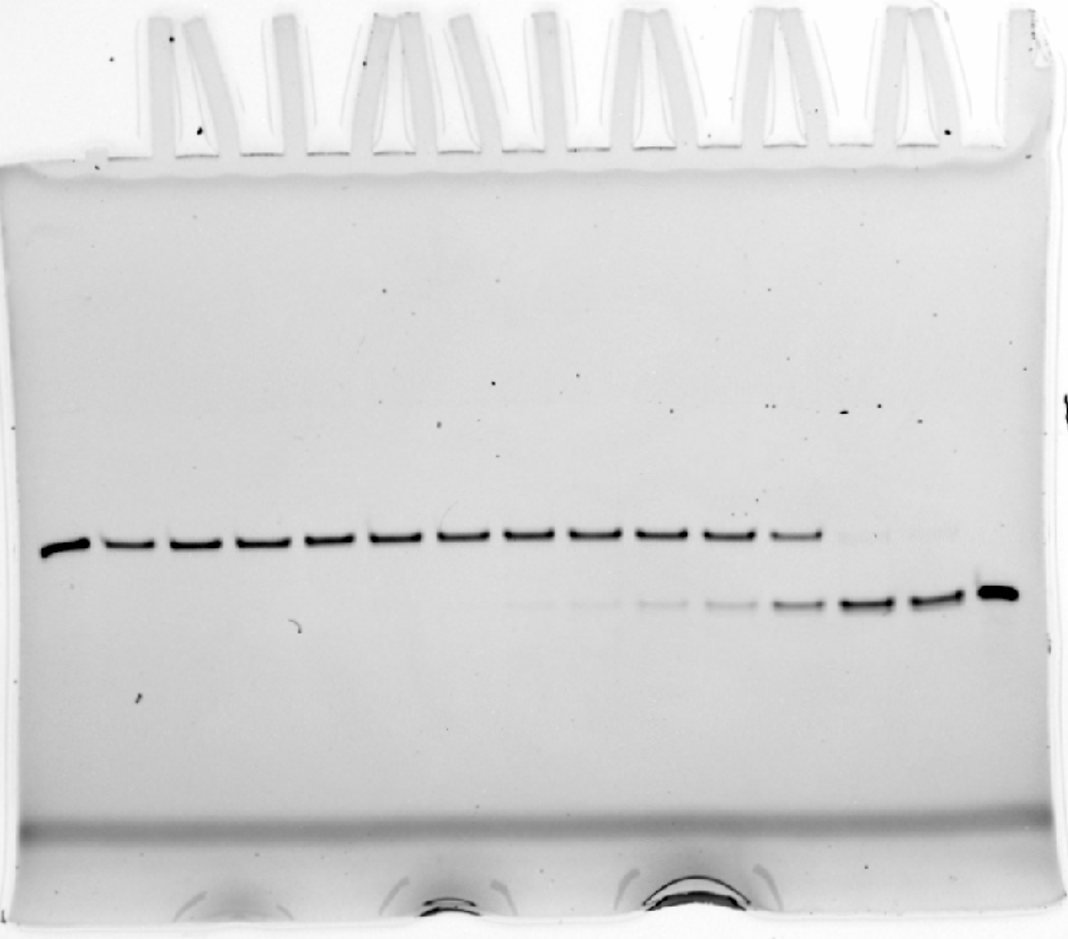

Supplement: Supplementary file 1 [file biomolecules-16-00715-s001.zip › Original-Images/FigS2B-1.tif]

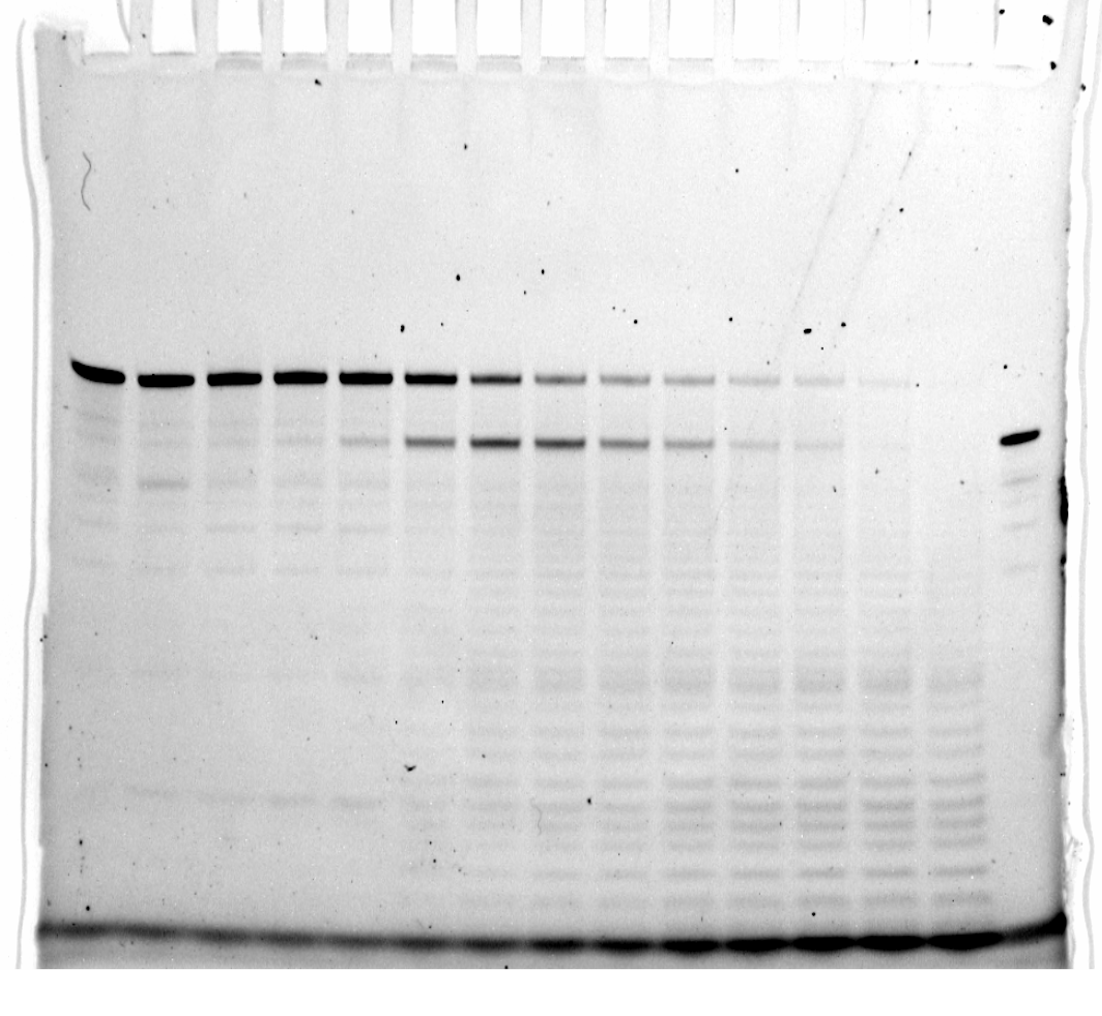

Supplement: Supplementary file 1 [file biomolecules-16-00715-s001.zip › Original-Images/FigS2B-2.tif]

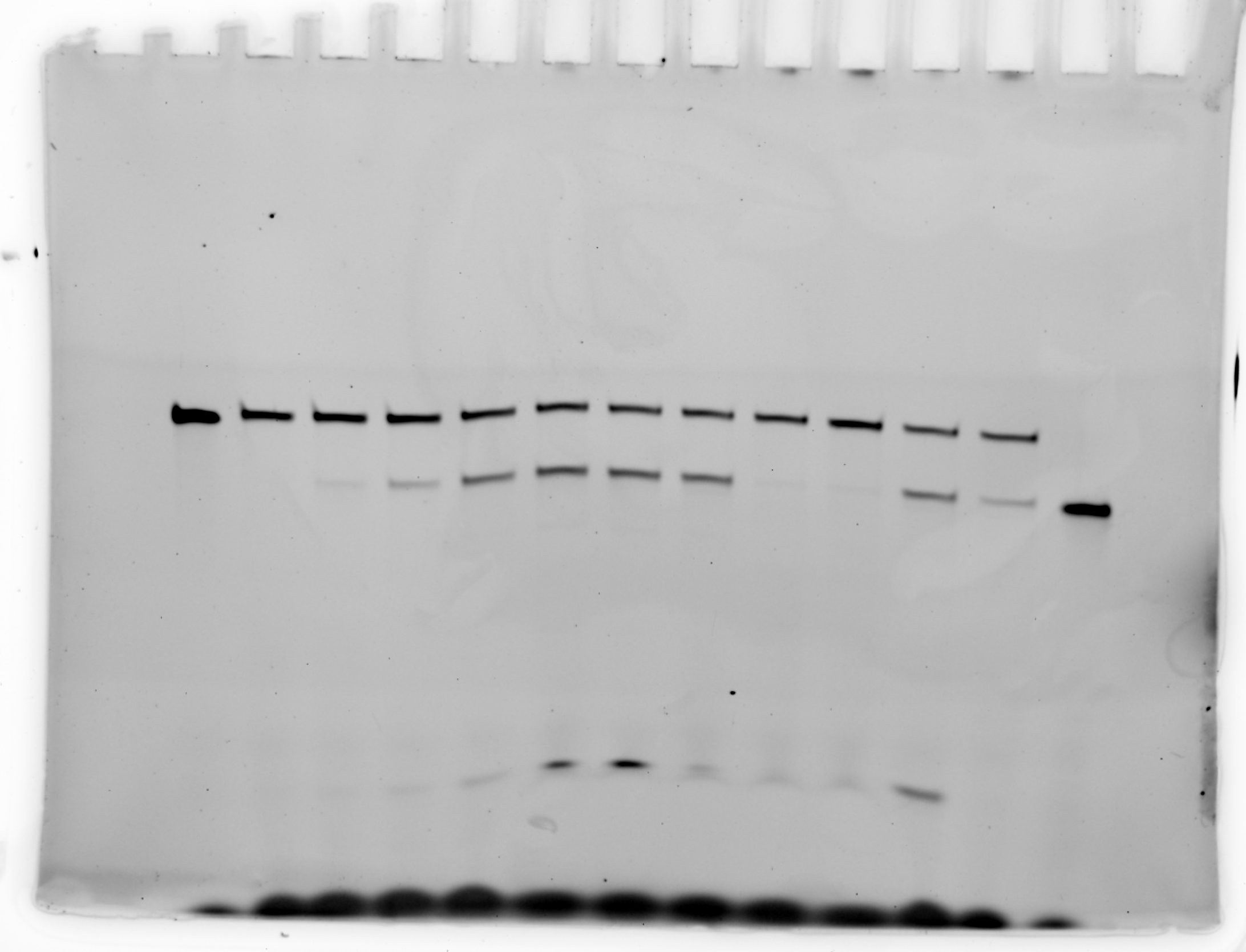

Supplement: Supplementary file 1 [file biomolecules-16-00715-s001.zip › Original-Images/FigS3A-1.tif]

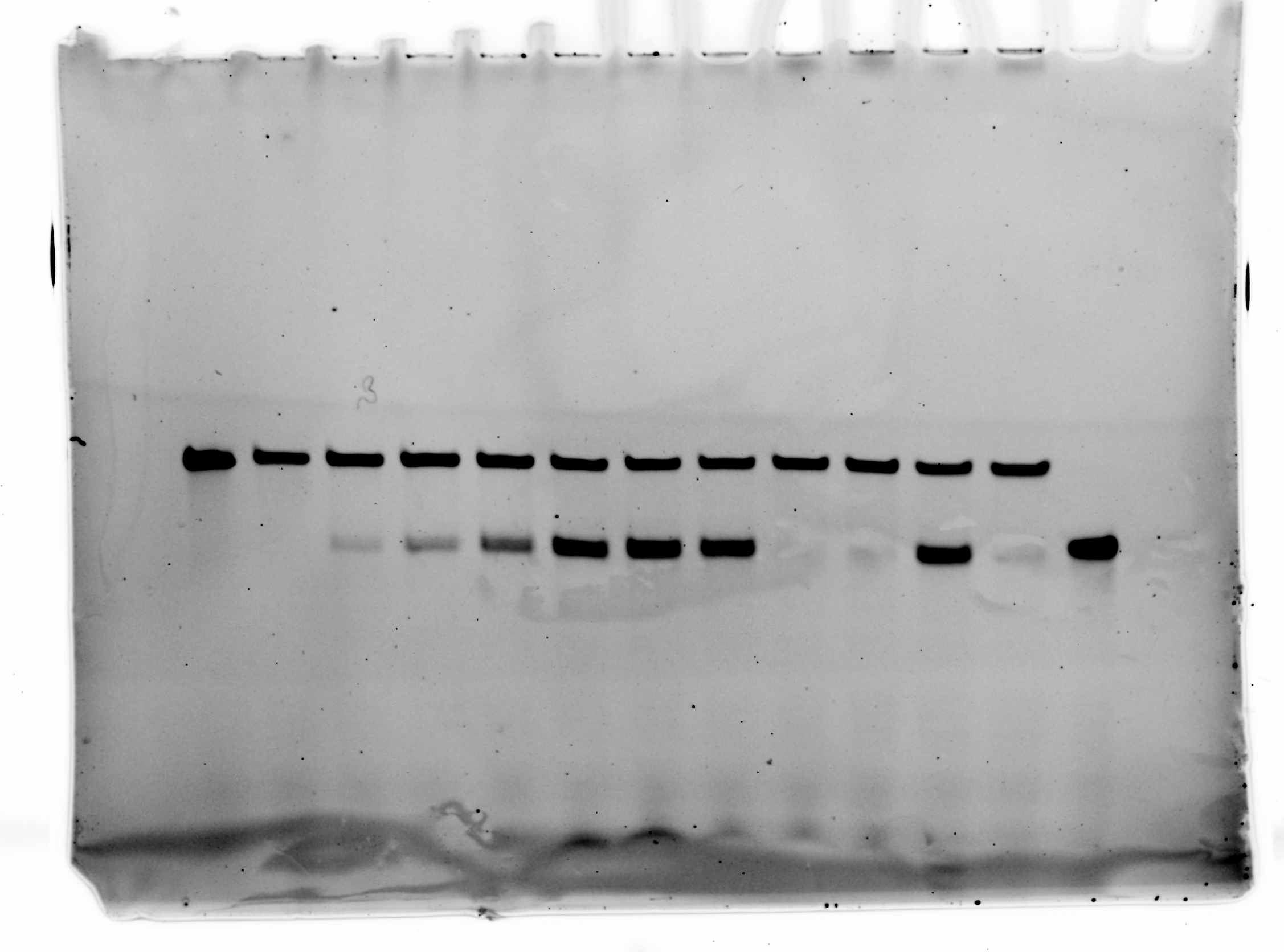

Supplement: Supplementary file 1 [file biomolecules-16-00715-s001.zip › Original-Images/FigS3A-2.tif]

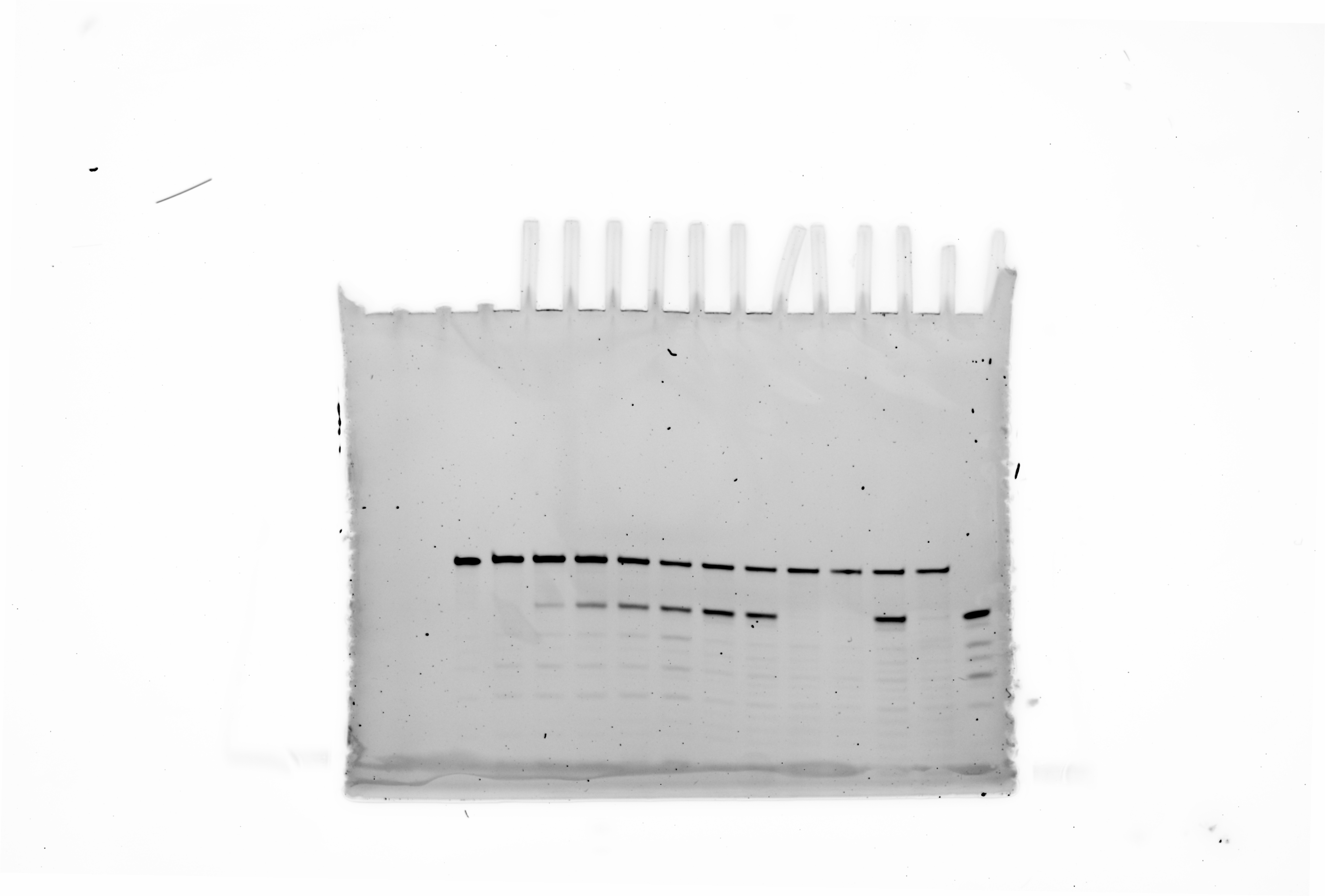

Supplement: Supplementary file 1 [file biomolecules-16-00715-s001.zip › Original-Images/FigS3A-3.tif]

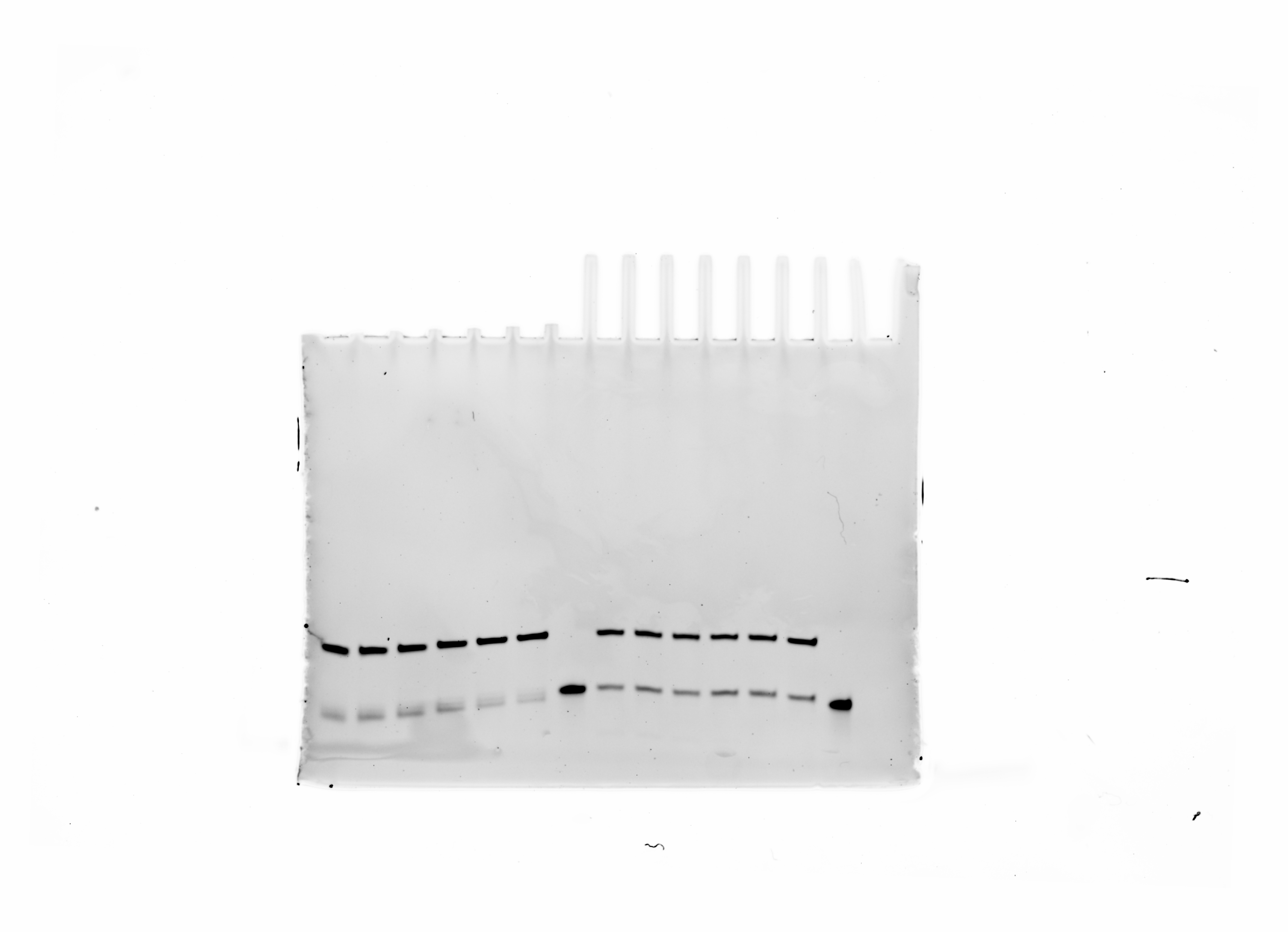

Supplement: Supplementary file 1 [file biomolecules-16-00715-s001.zip › Original-Images/FigS3B-1.tif]

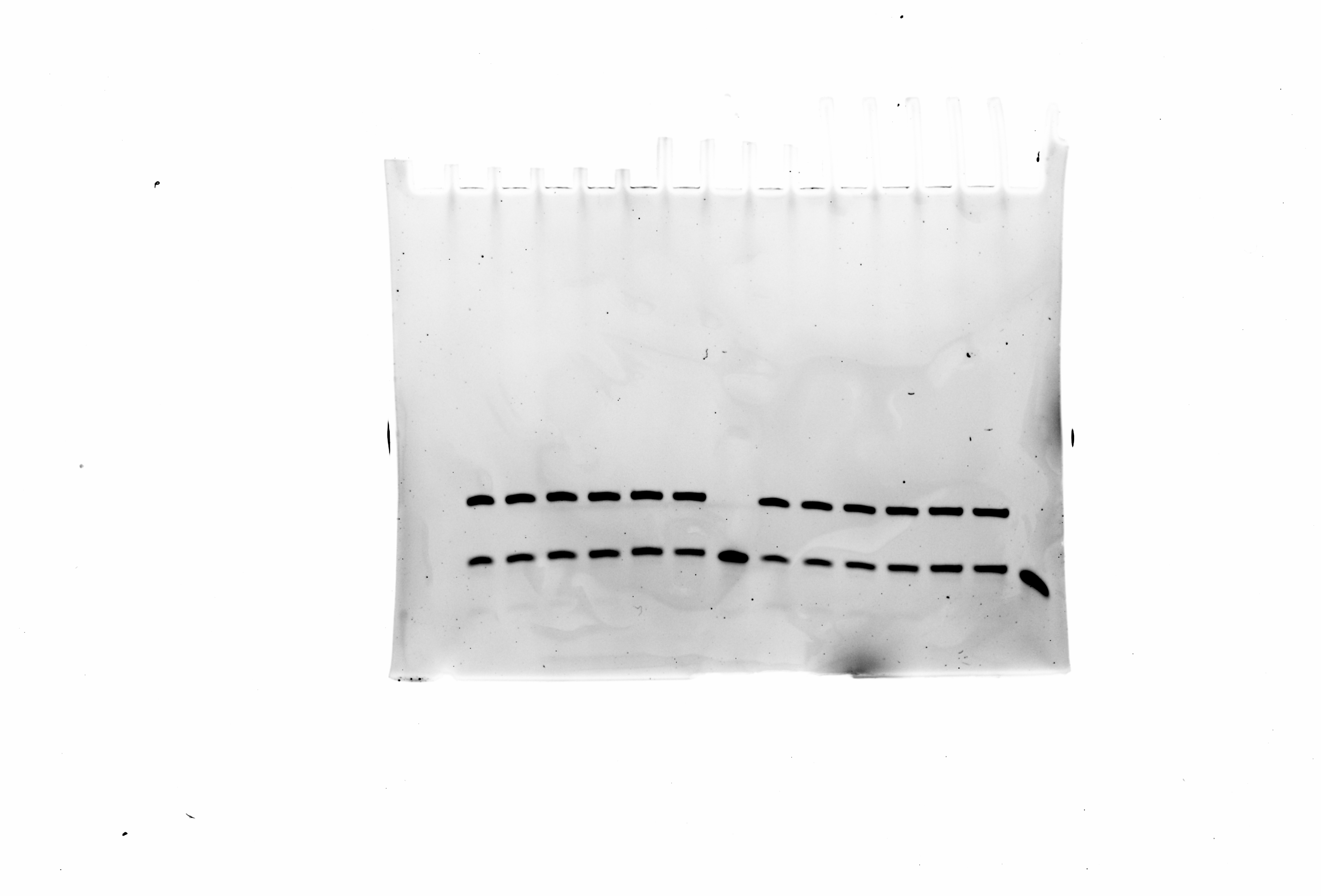

Supplement: Supplementary file 1 [file biomolecules-16-00715-s001.zip › Original-Images/FigS3B-2.tif]

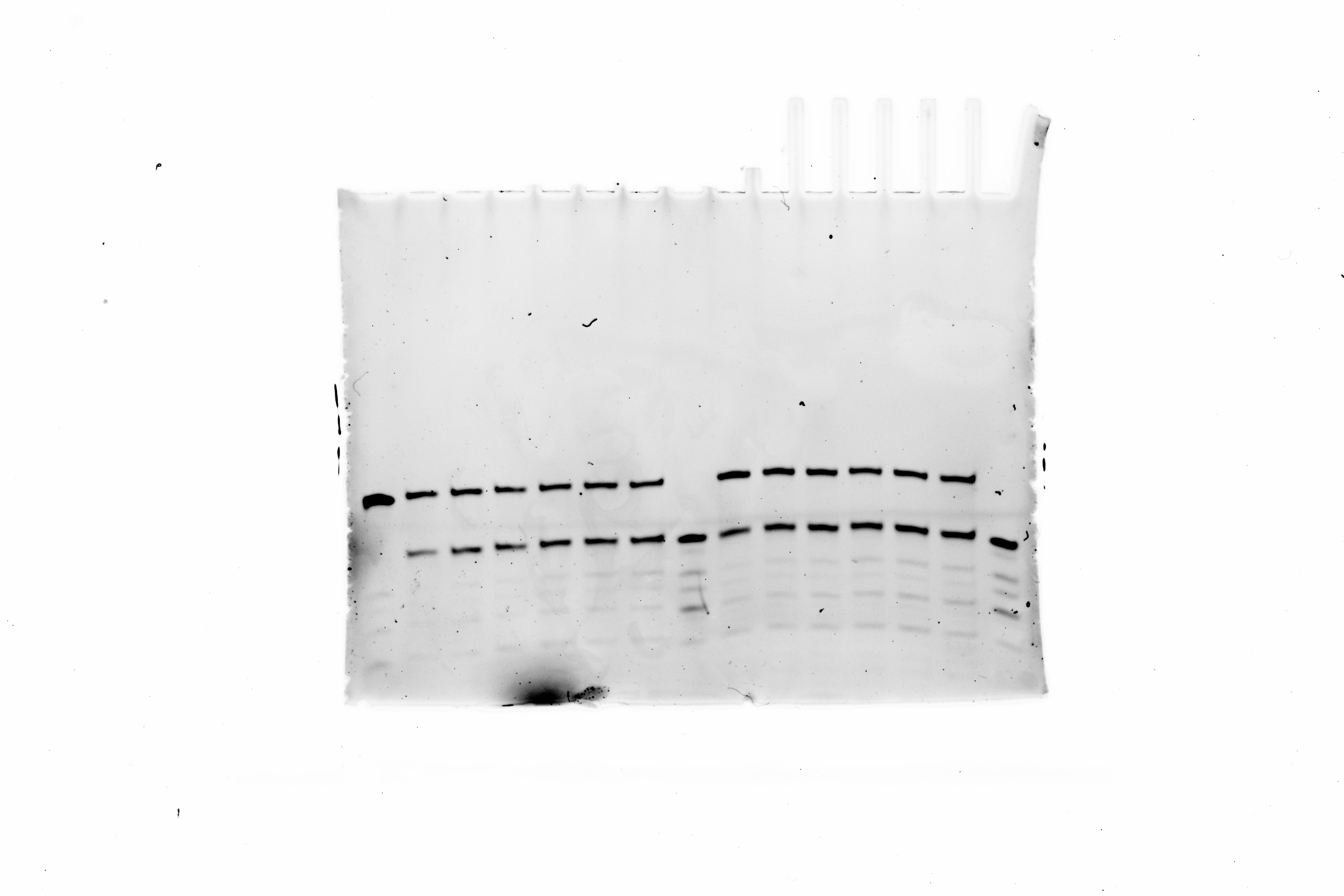

Supplement: Supplementary file 1 [file biomolecules-16-00715-s001.zip › Original-Images/FigS3B-3.tif]

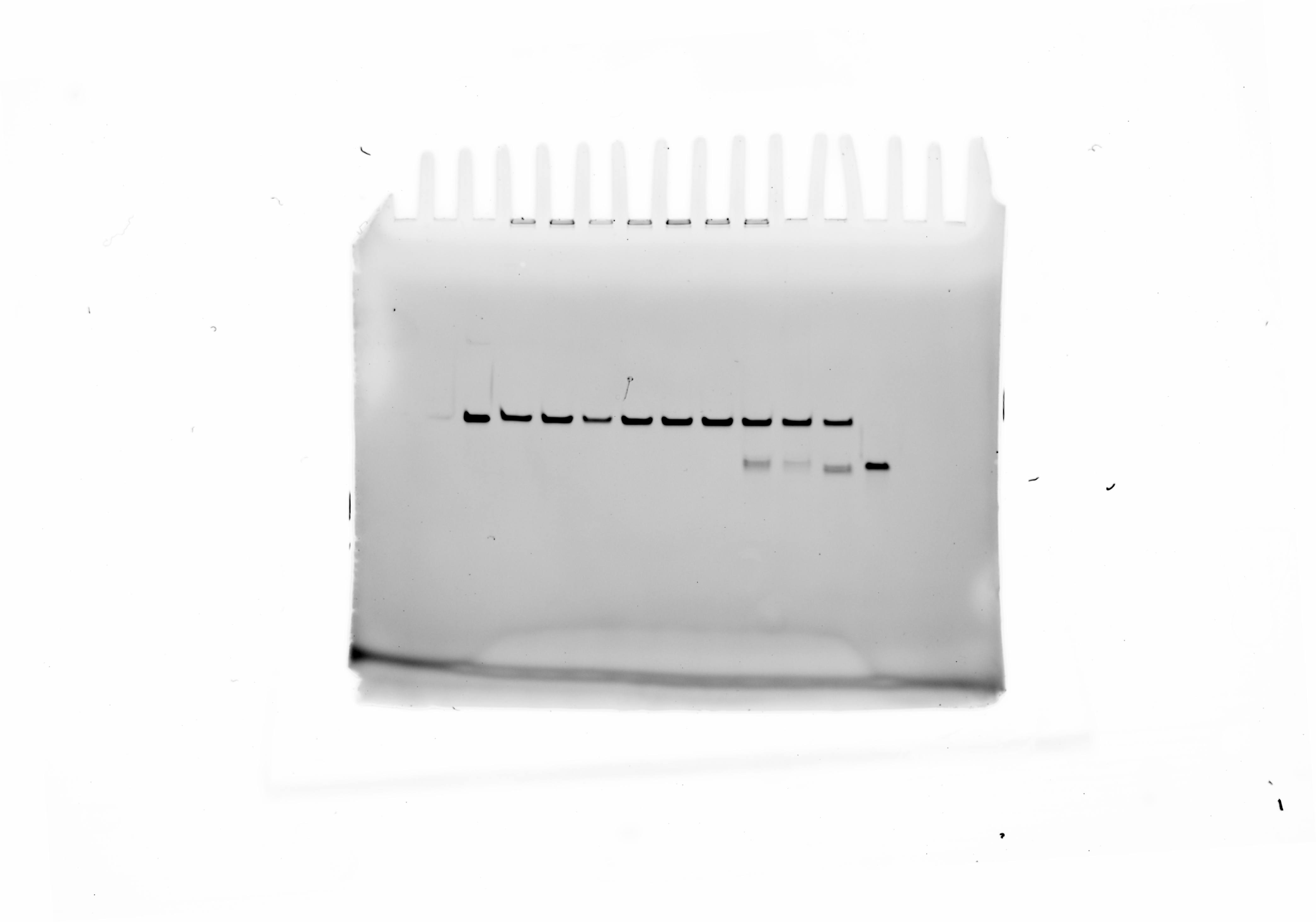

Supplement: Supplementary file 1 [file biomolecules-16-00715-s001.zip › Original-Images/FigS3C-1.tif]

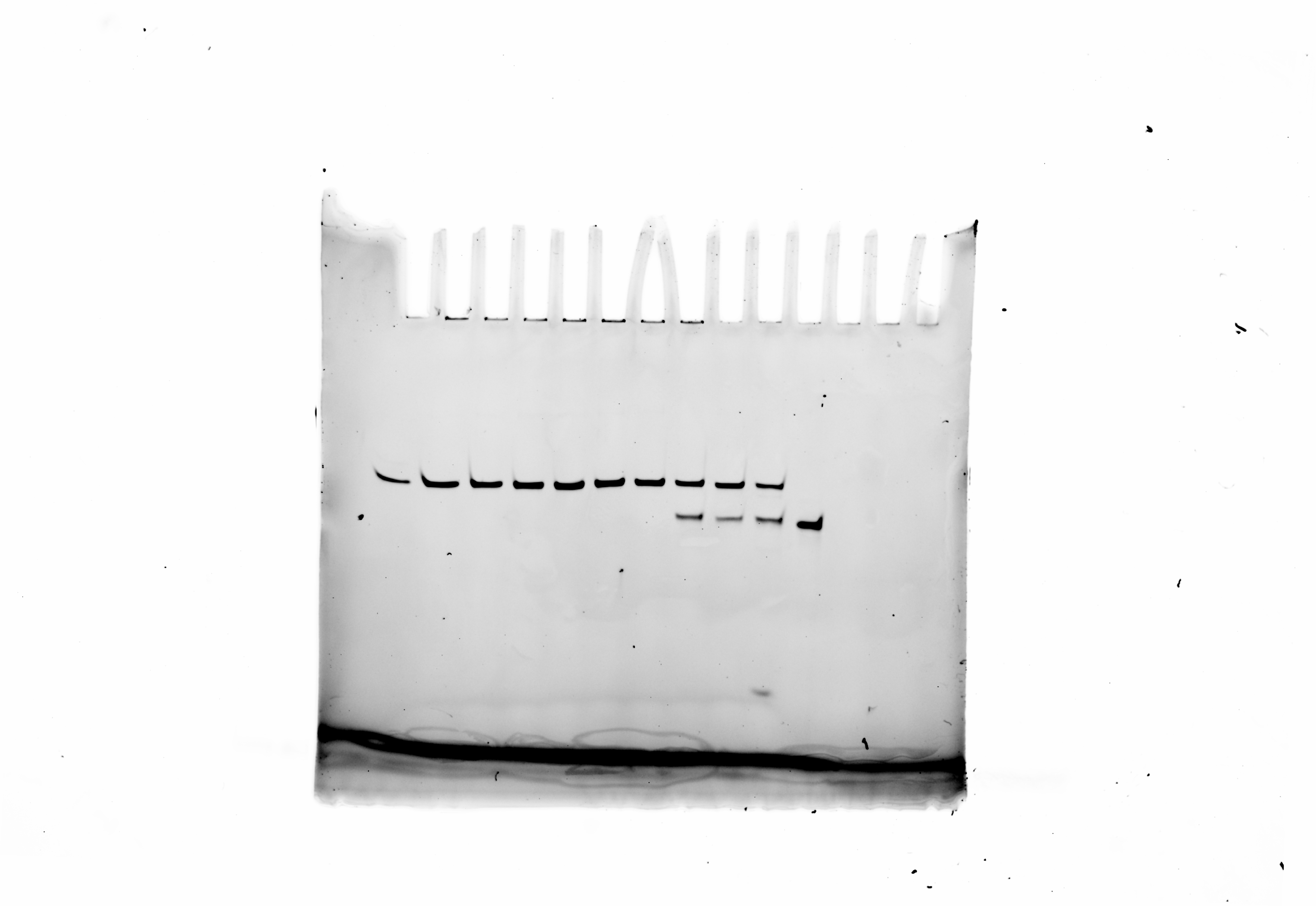

Supplement: Supplementary file 1 [file biomolecules-16-00715-s001.zip › Original-Images/FigS3C-2.tif]

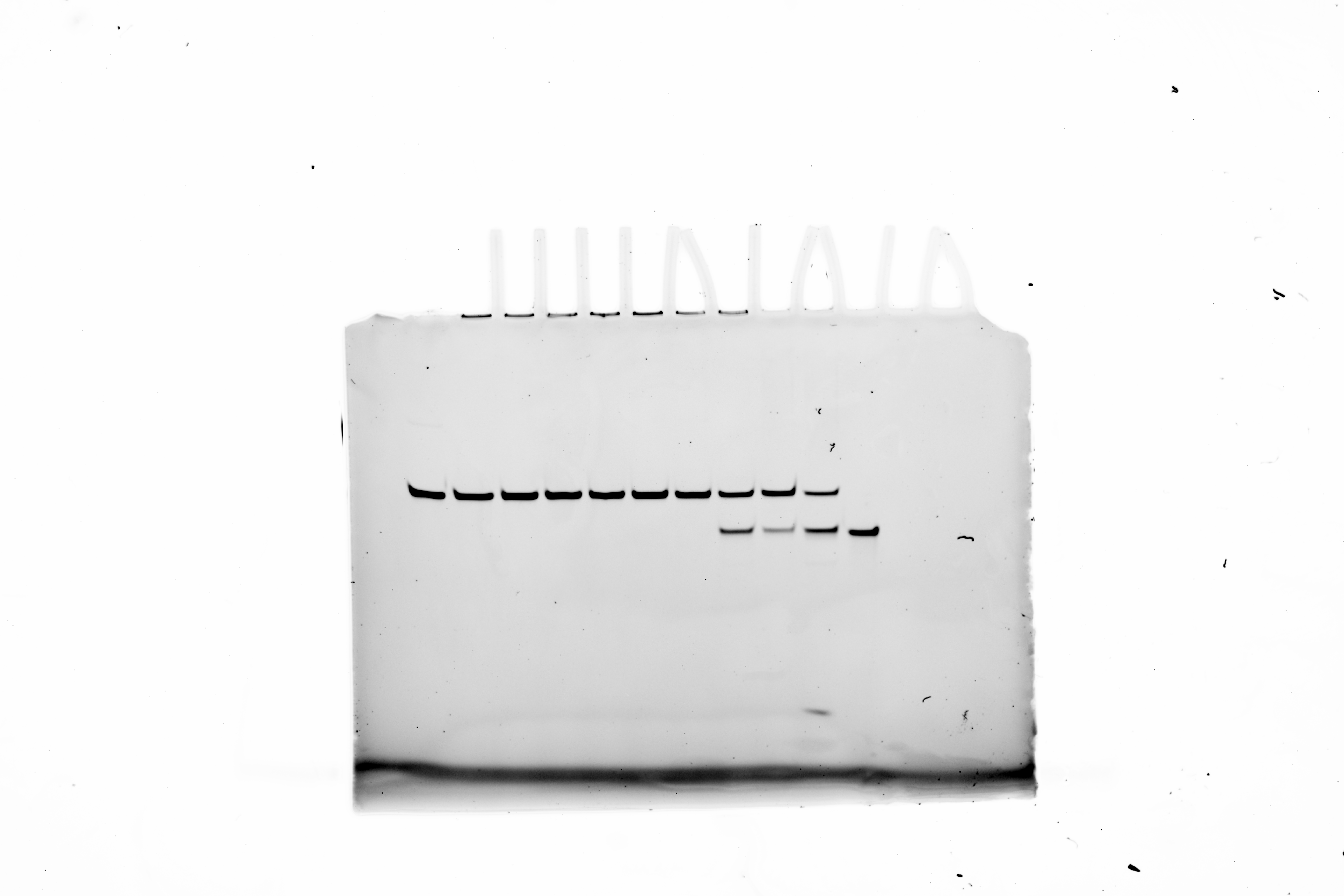

Supplement: Supplementary file 1 [file biomolecules-16-00715-s001.zip › Original-Images/FigS3C-3.tif]

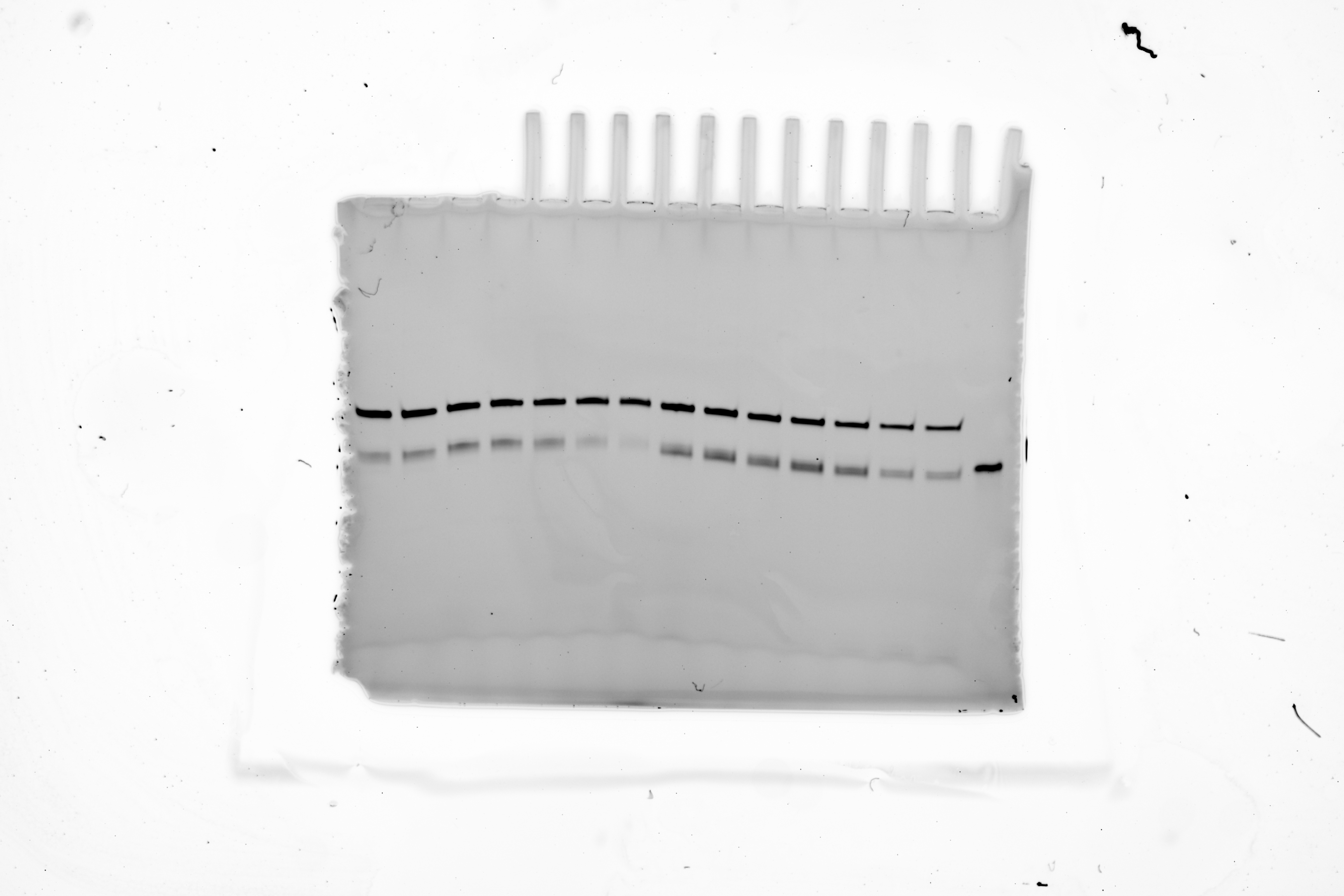

Supplement: Supplementary file 1 [file biomolecules-16-00715-s001.zip › Original-Images/FigS3D-1.tif]

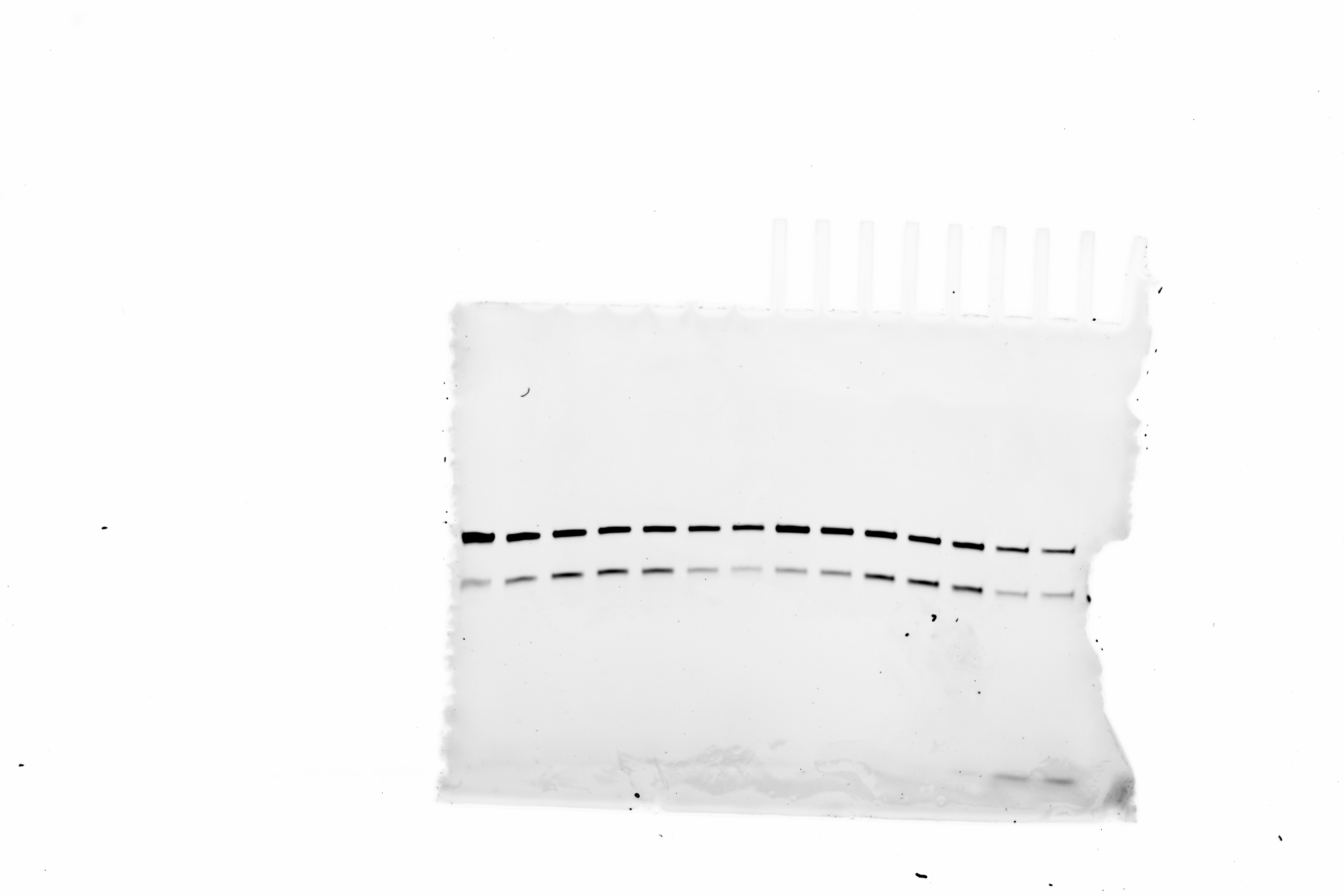

Supplement: Supplementary file 1 [file biomolecules-16-00715-s001.zip › Original-Images/FigS3D-2.tif]

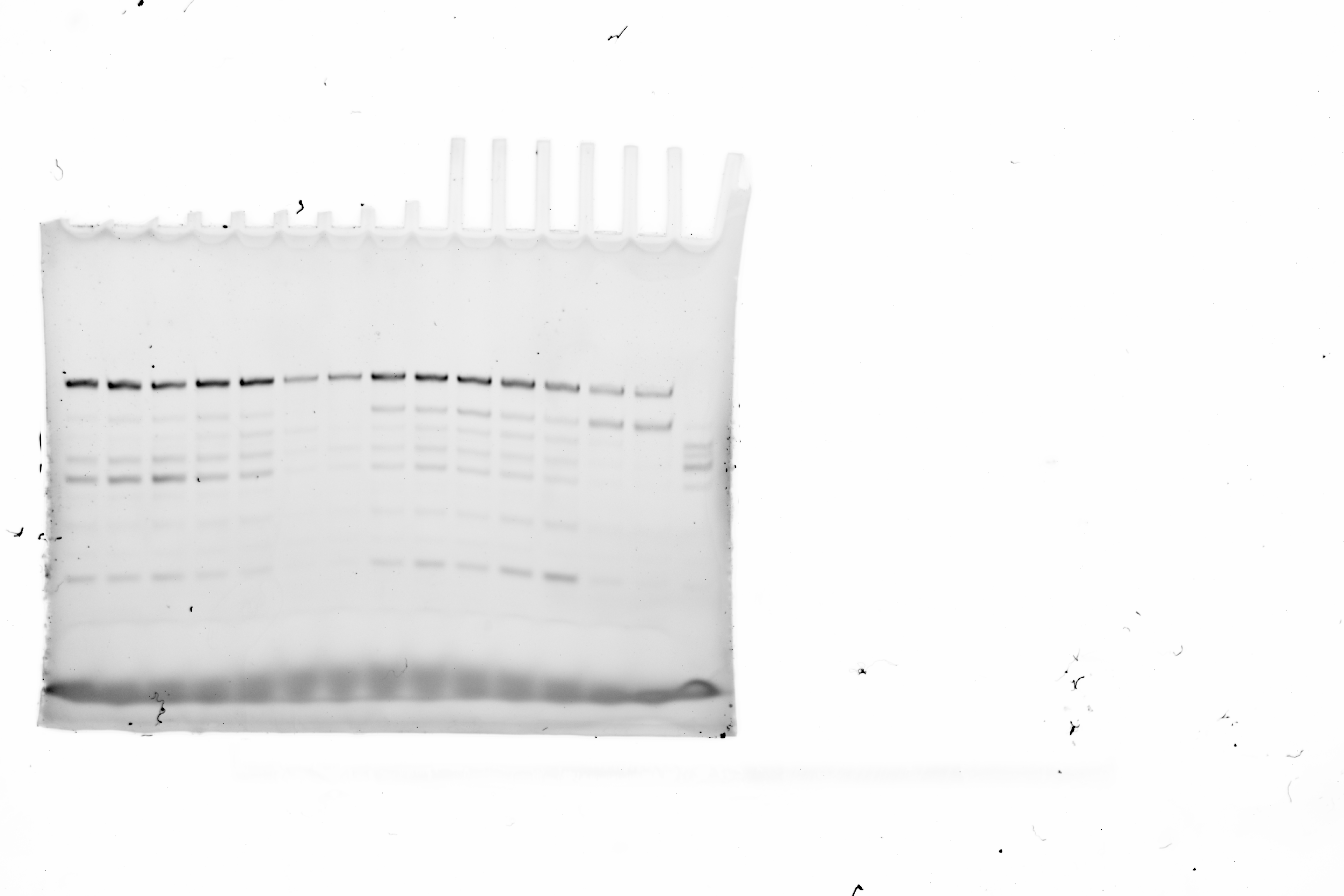

Supplement: Supplementary file 1 [file biomolecules-16-00715-s001.zip › Original-Images/FigS3D-3-1.tif]

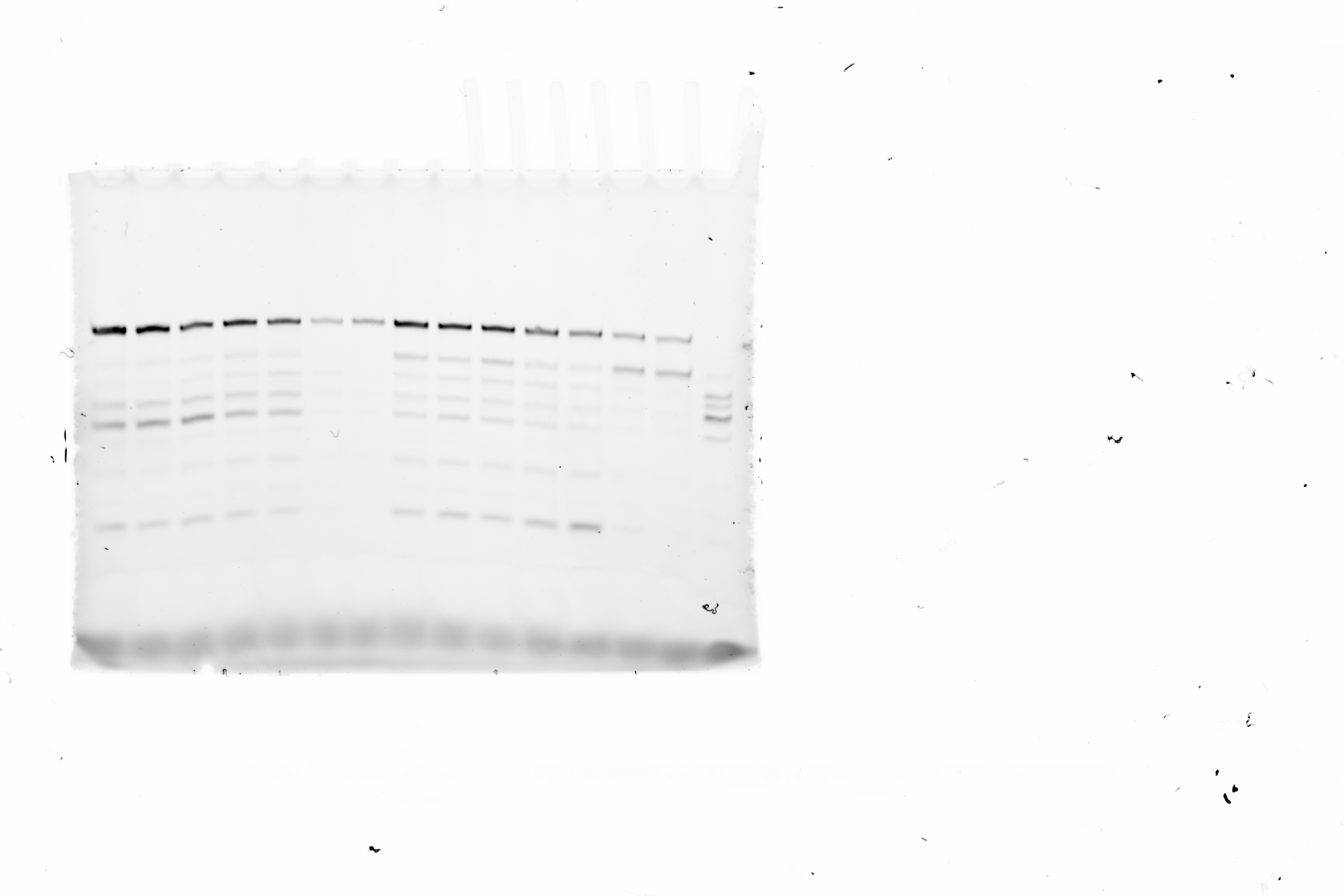

Supplement: Supplementary file 1 [file biomolecules-16-00715-s001.zip › Original-Images/FigS3D-3-2.tif]

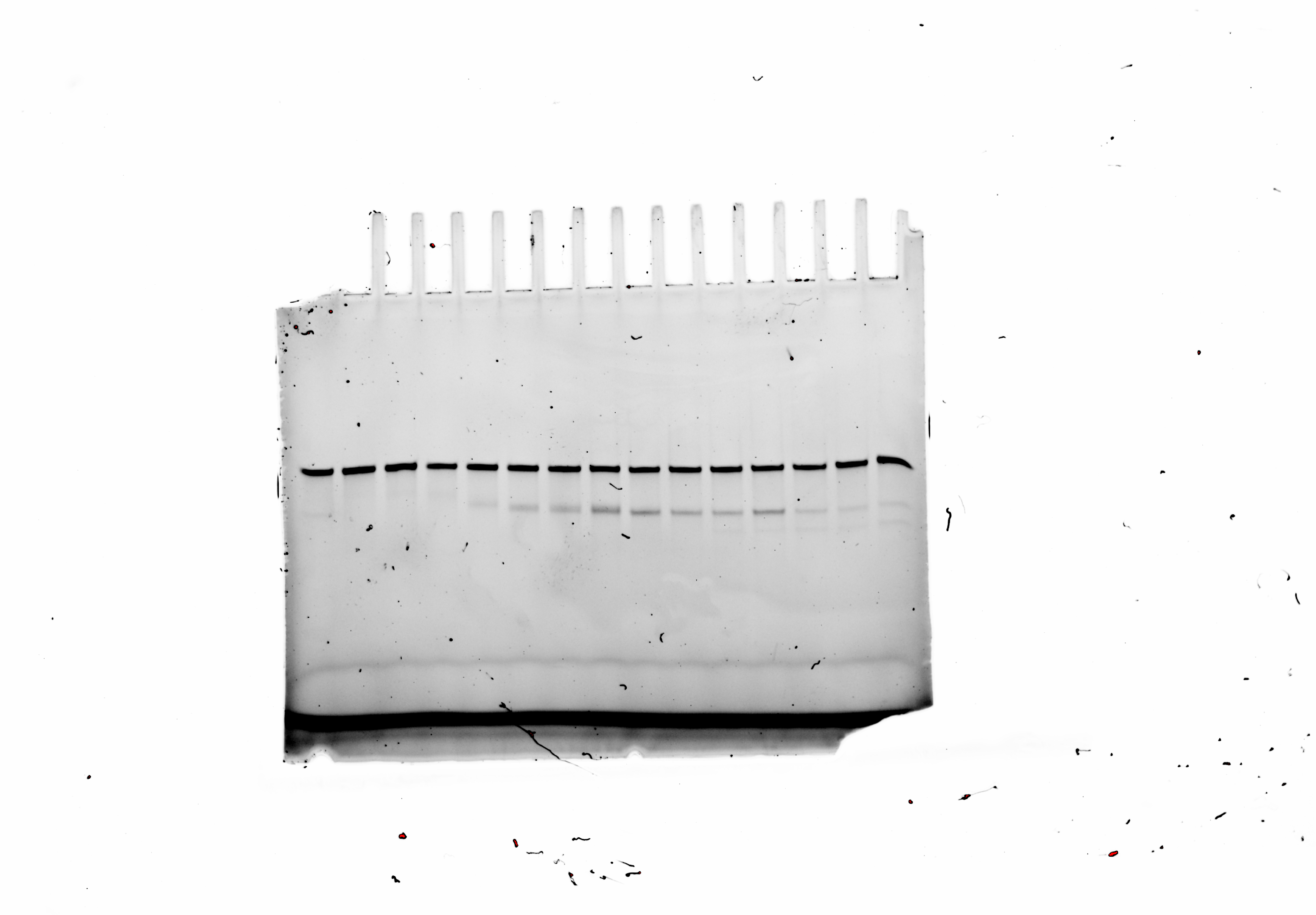

Supplement: Supplementary file 1 [file biomolecules-16-00715-s001.zip › Original-Images/FigS4A-1.tif]

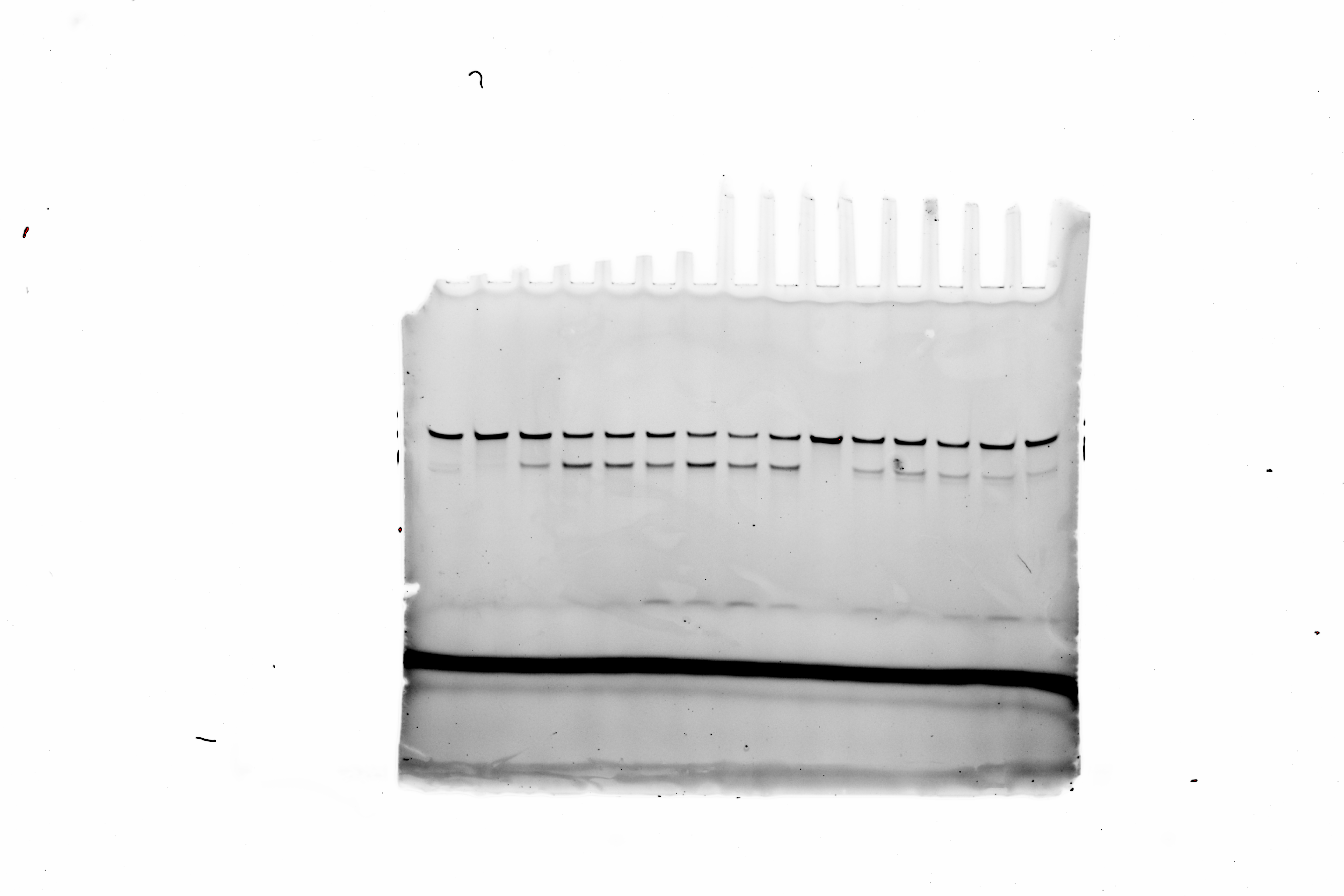

Supplement: Supplementary file 1 [file biomolecules-16-00715-s001.zip › Original-Images/FigS4A-2-1.tif]

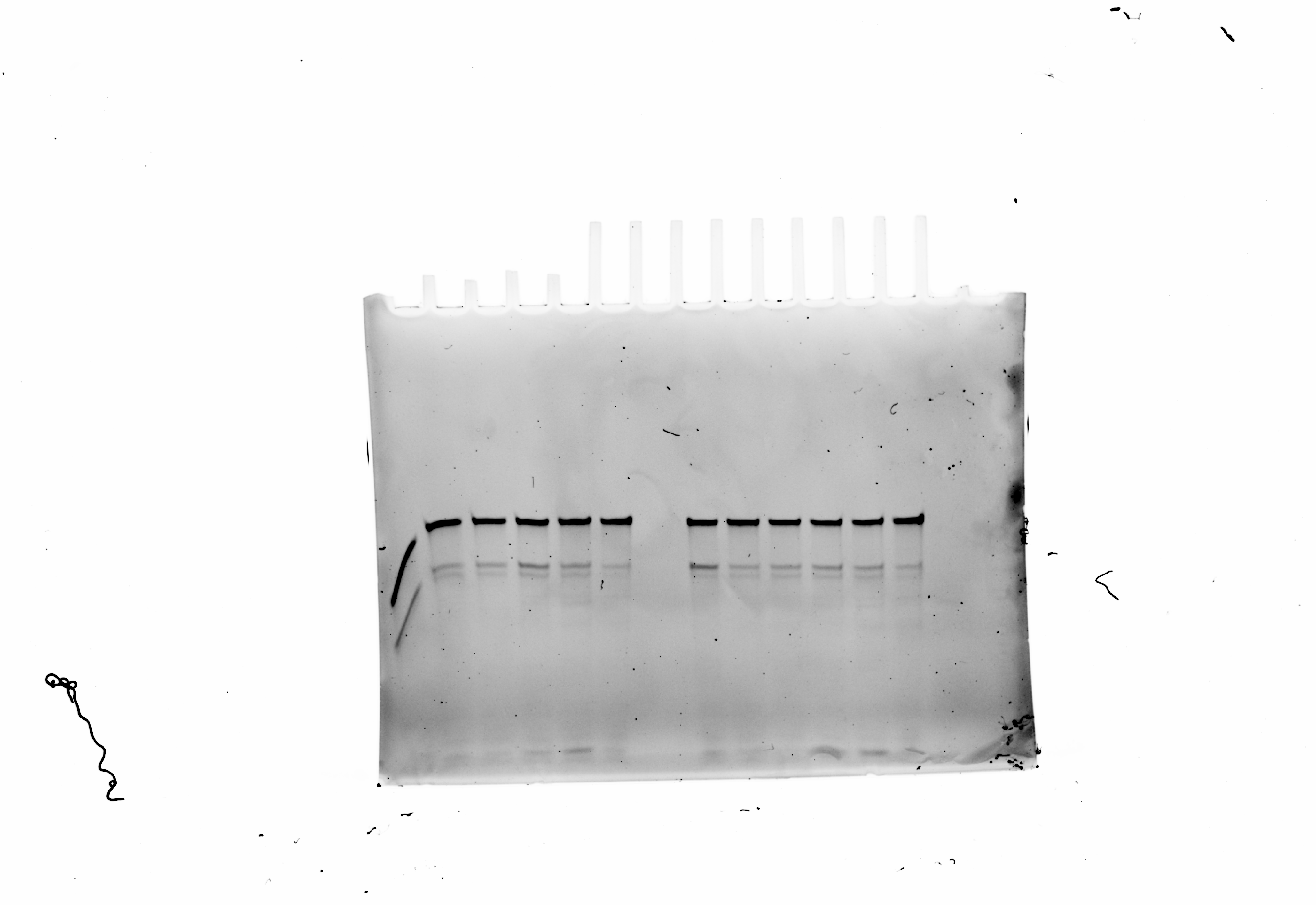

Supplement: Supplementary file 1 [file biomolecules-16-00715-s001.zip › Original-Images/FigS4A-2-2.tif]

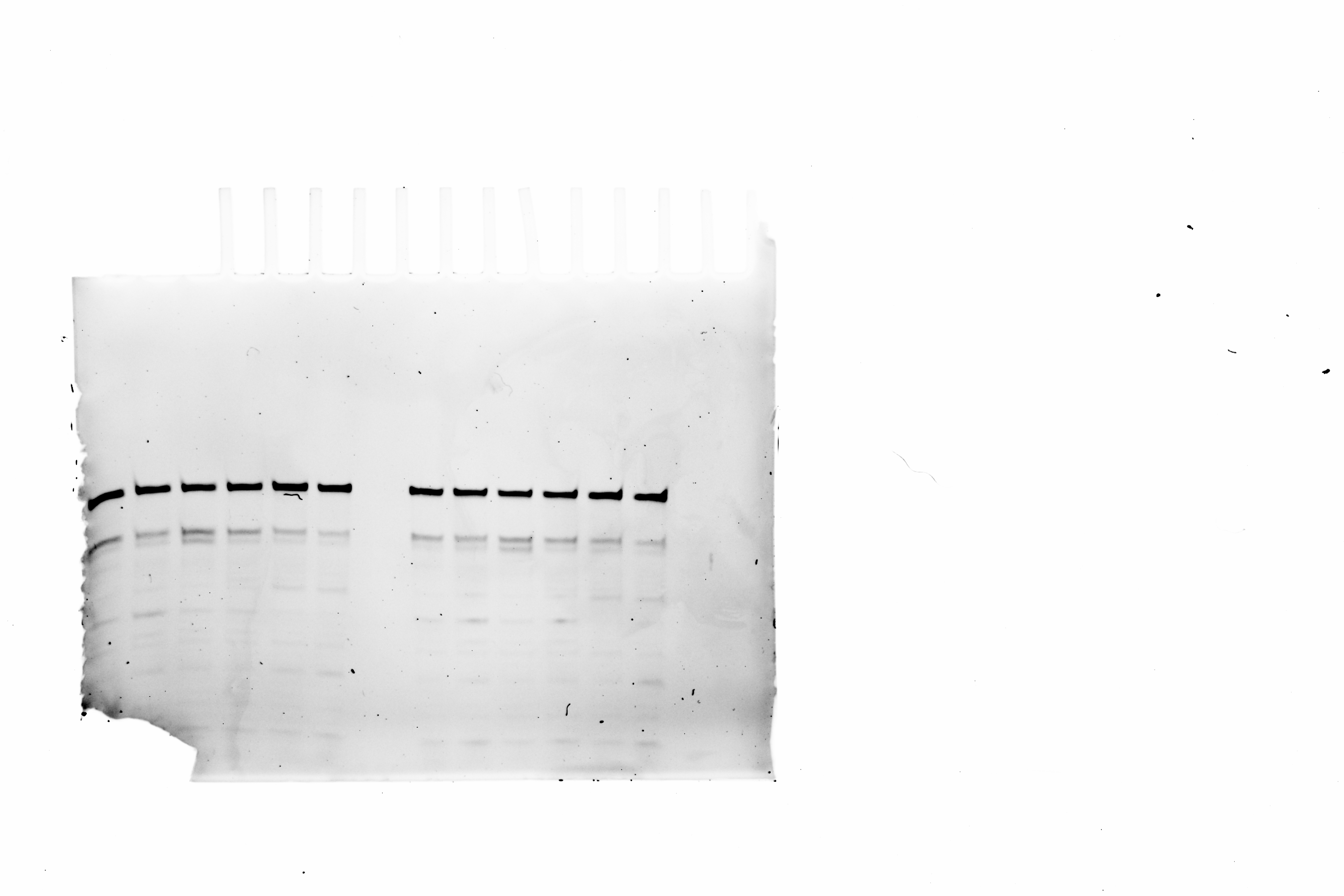

Supplement: Supplementary file 1 [file biomolecules-16-00715-s001.zip › Original-Images/FigS4A-3-2.tif]

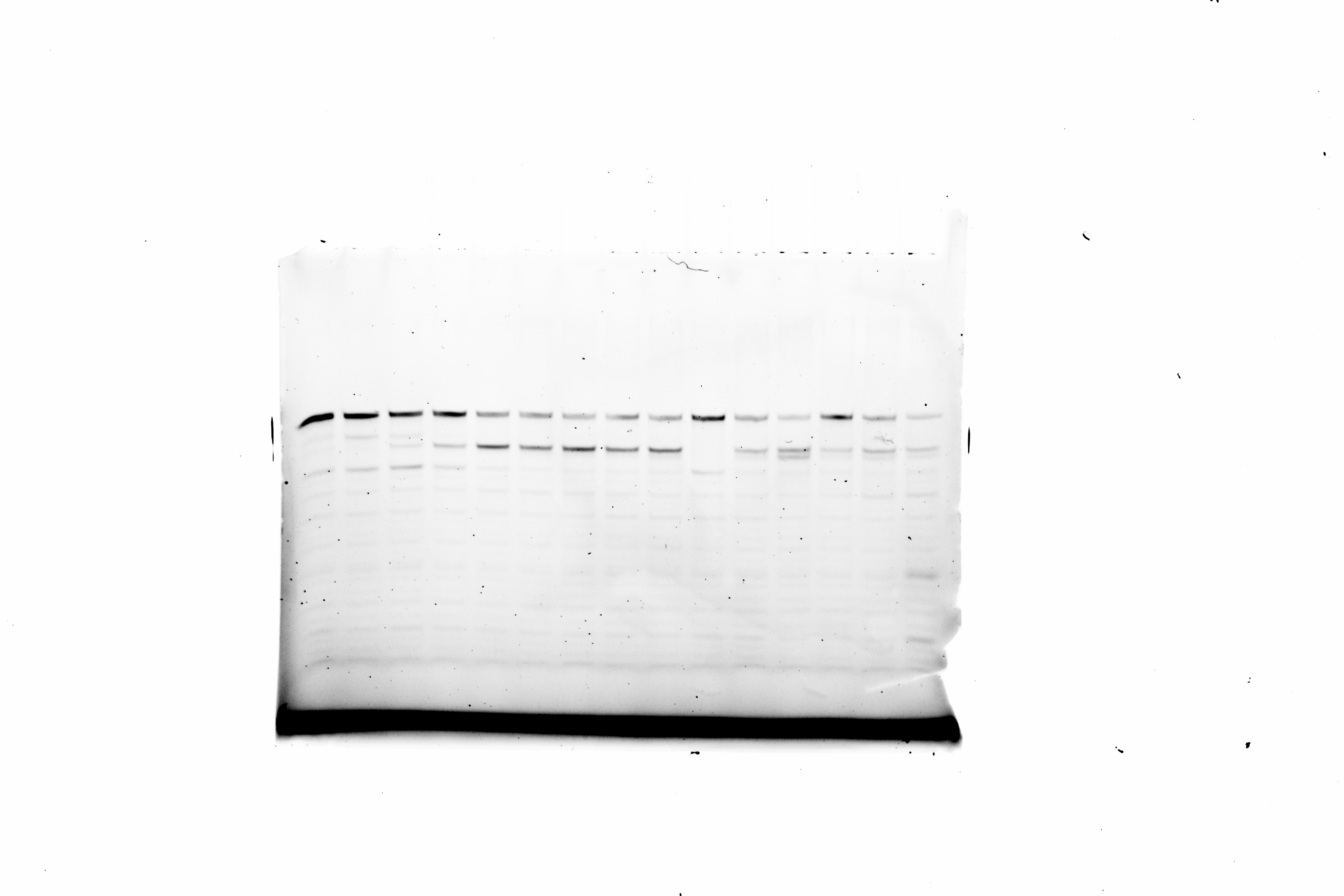

Supplement: Supplementary file 1 [file biomolecules-16-00715-s001.zip › Original-Images/FigS4A-3.tif]

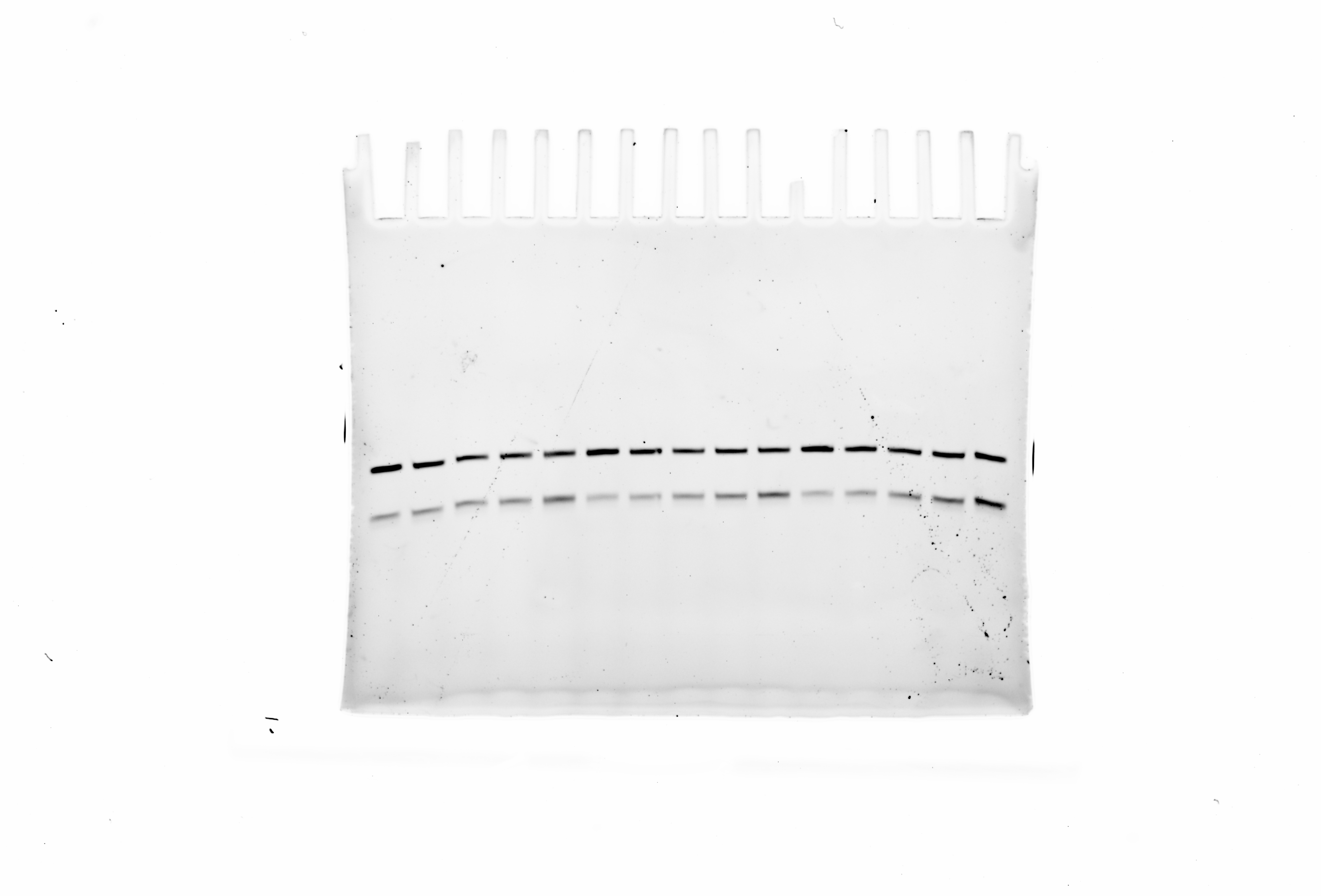

Supplement: Supplementary file 1 [file biomolecules-16-00715-s001.zip › Original-Images/FigS4B-1-A.tif]

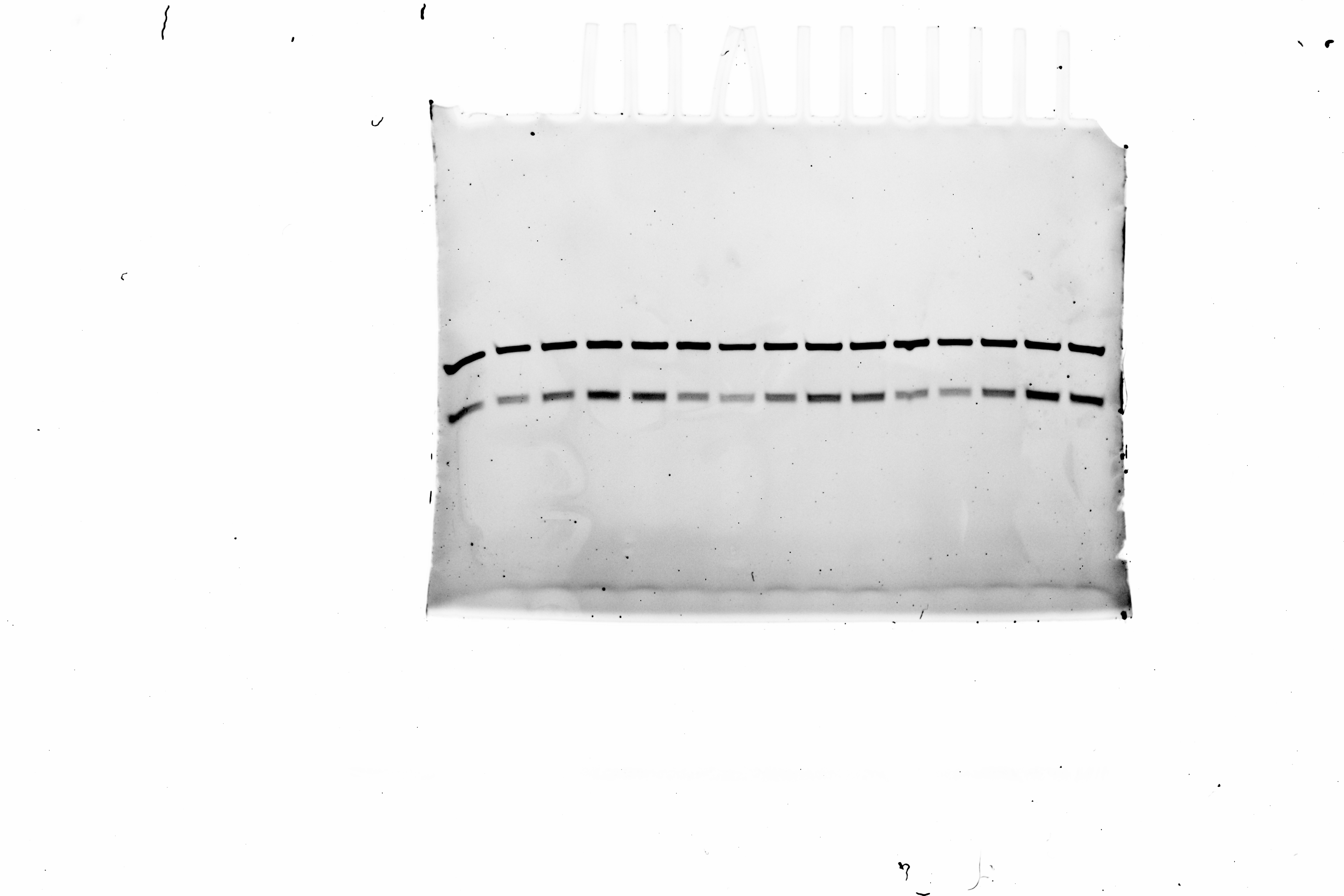

Supplement: Supplementary file 1 [file biomolecules-16-00715-s001.zip › Original-Images/FigS4B-1-C.tif]

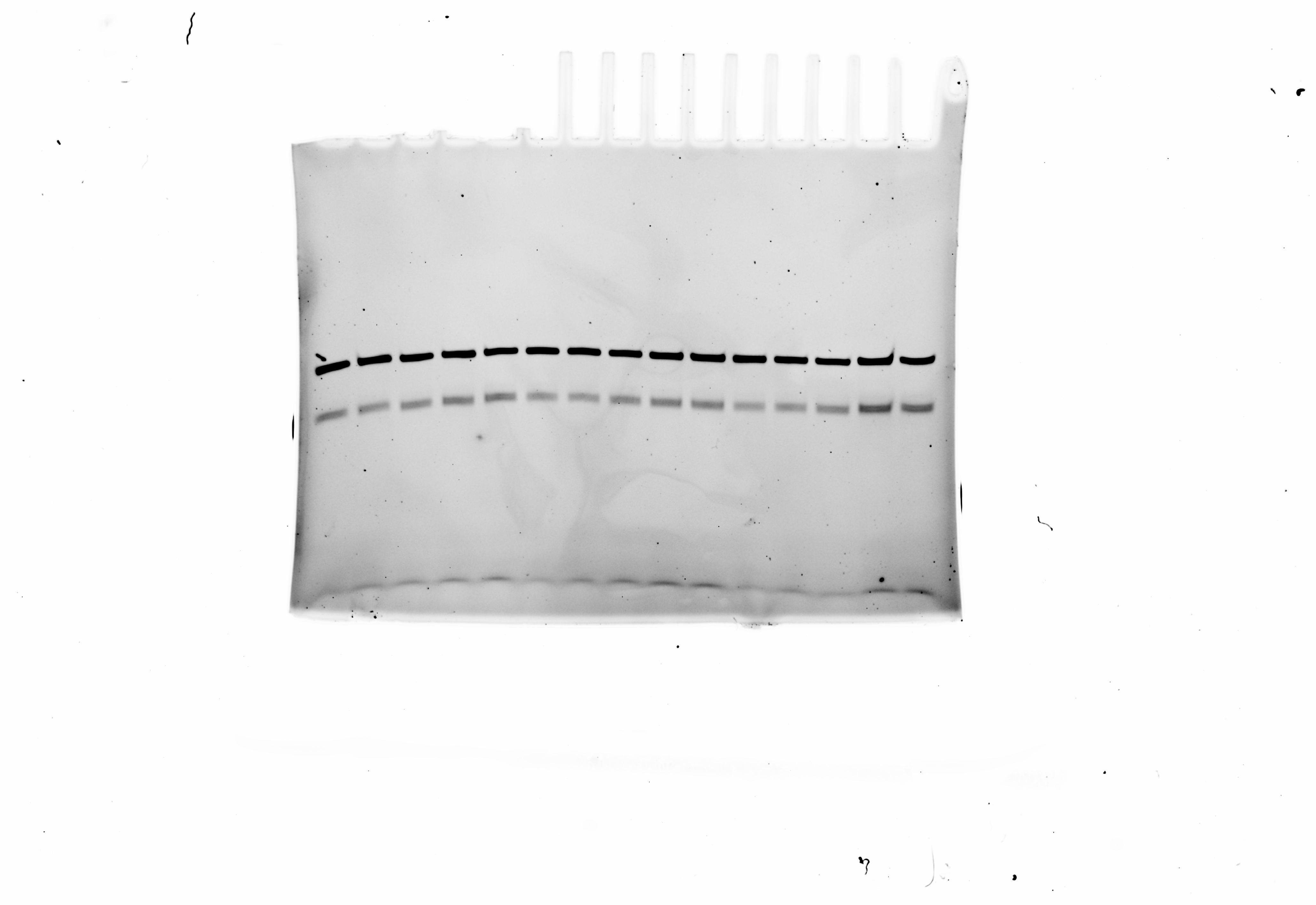

Supplement: Supplementary file 1 [file biomolecules-16-00715-s001.zip › Original-Images/FigS4B-1-G.tif]

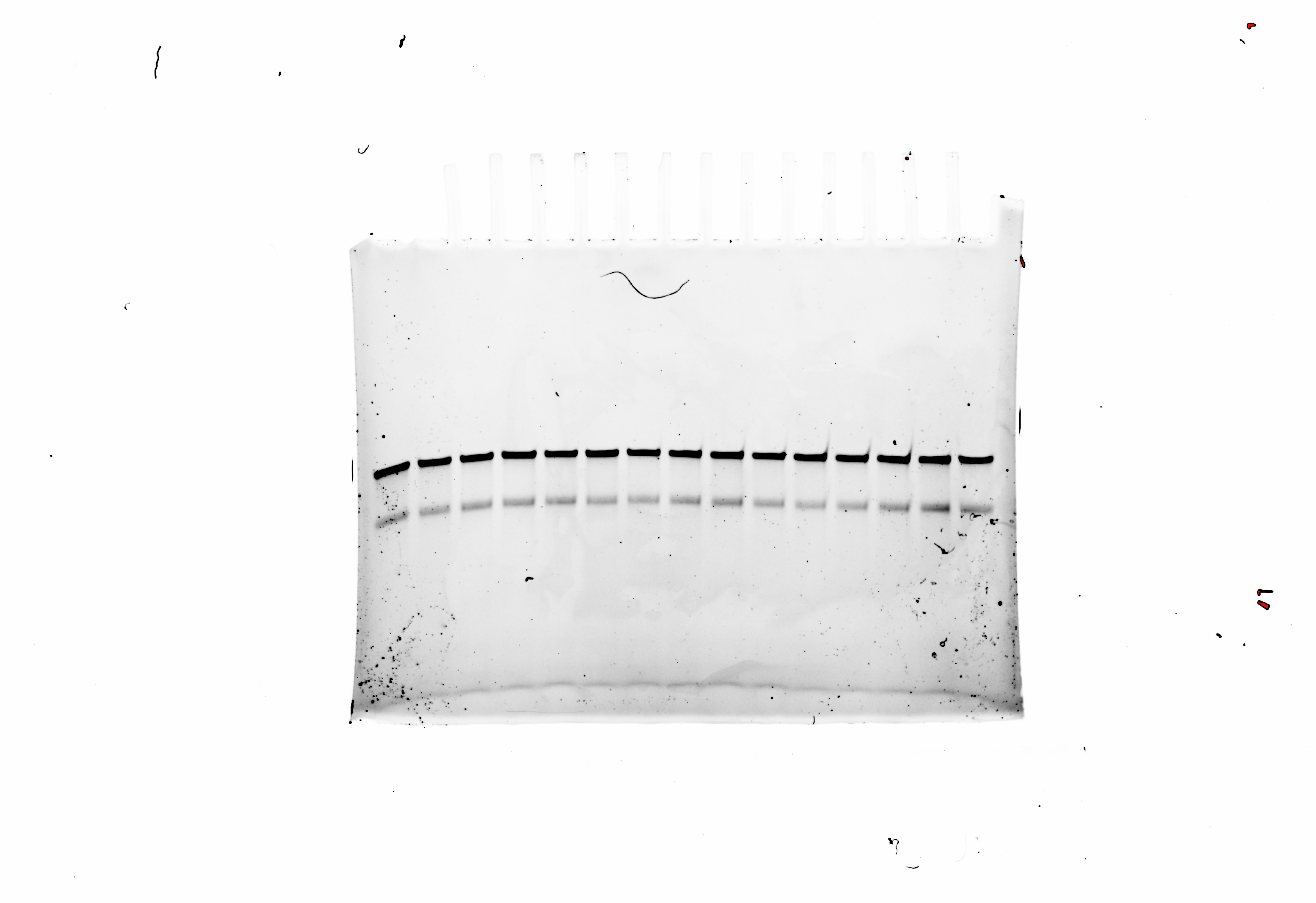

Supplement: Supplementary file 1 [file biomolecules-16-00715-s001.zip › Original-Images/FigS4B-1-T.tif]

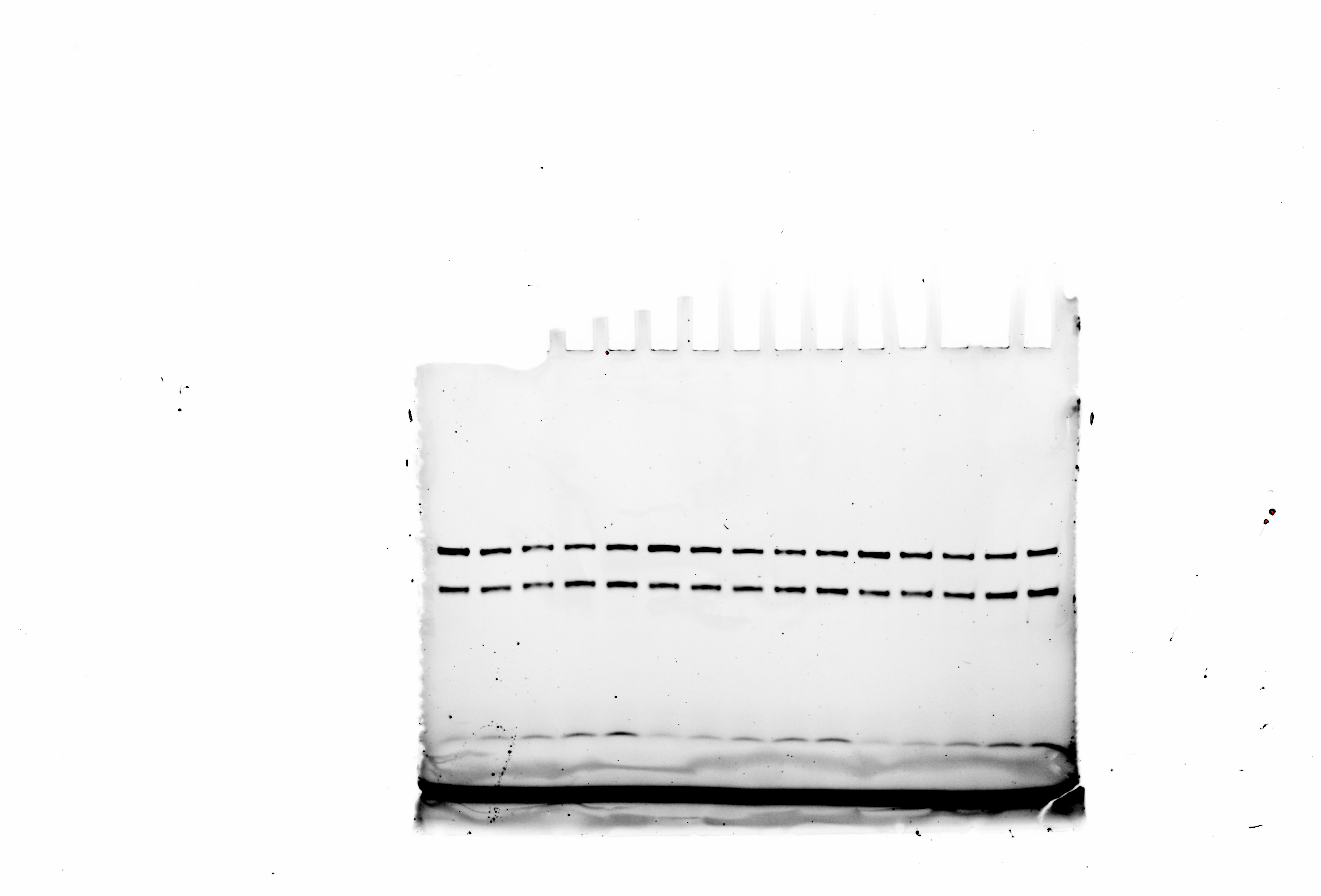

Supplement: Supplementary file 1 [file biomolecules-16-00715-s001.zip › Original-Images/FigS4B-2-A.tif]

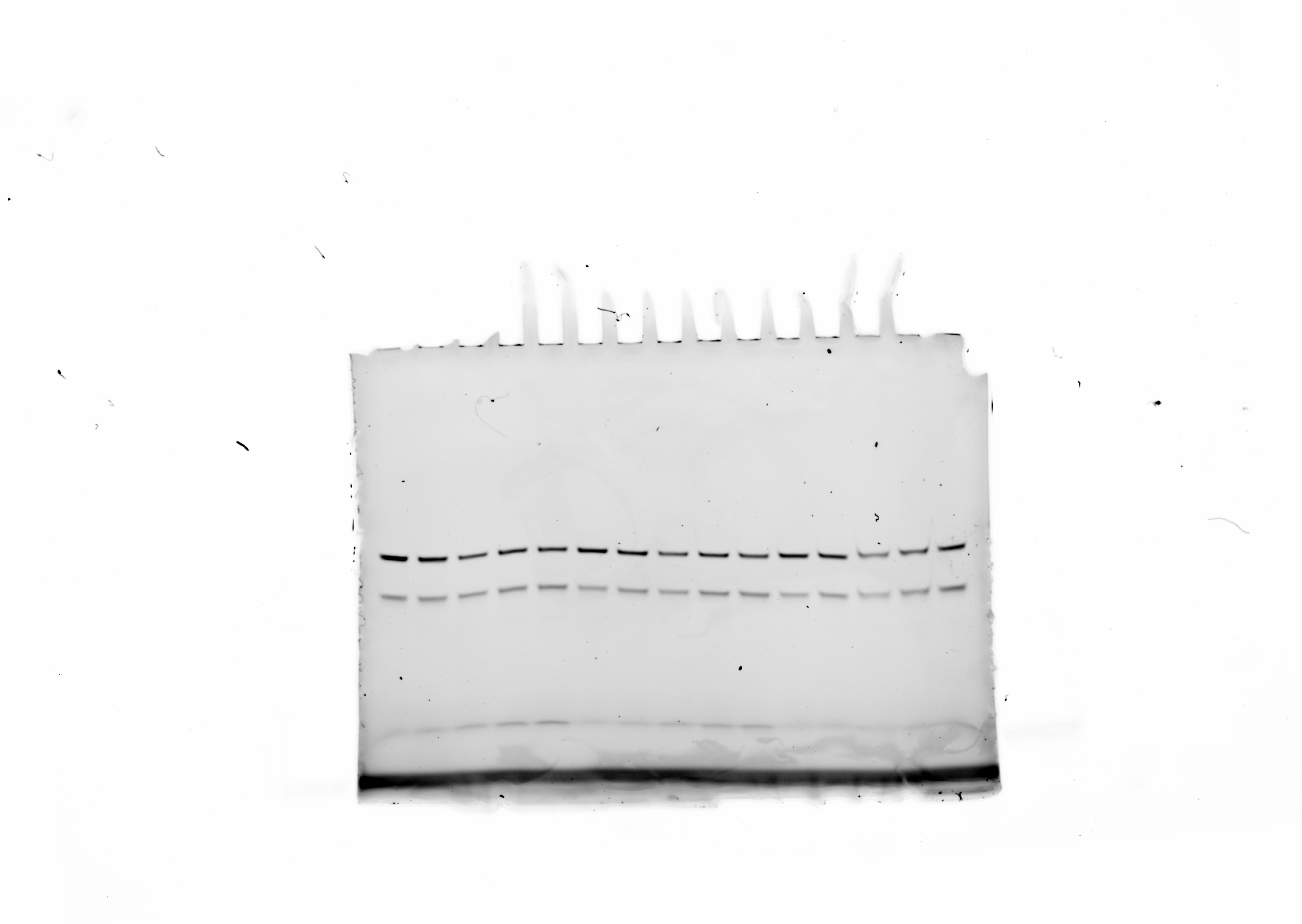

Supplement: Supplementary file 1 [file biomolecules-16-00715-s001.zip › Original-Images/FigS4B-2-C.tif]

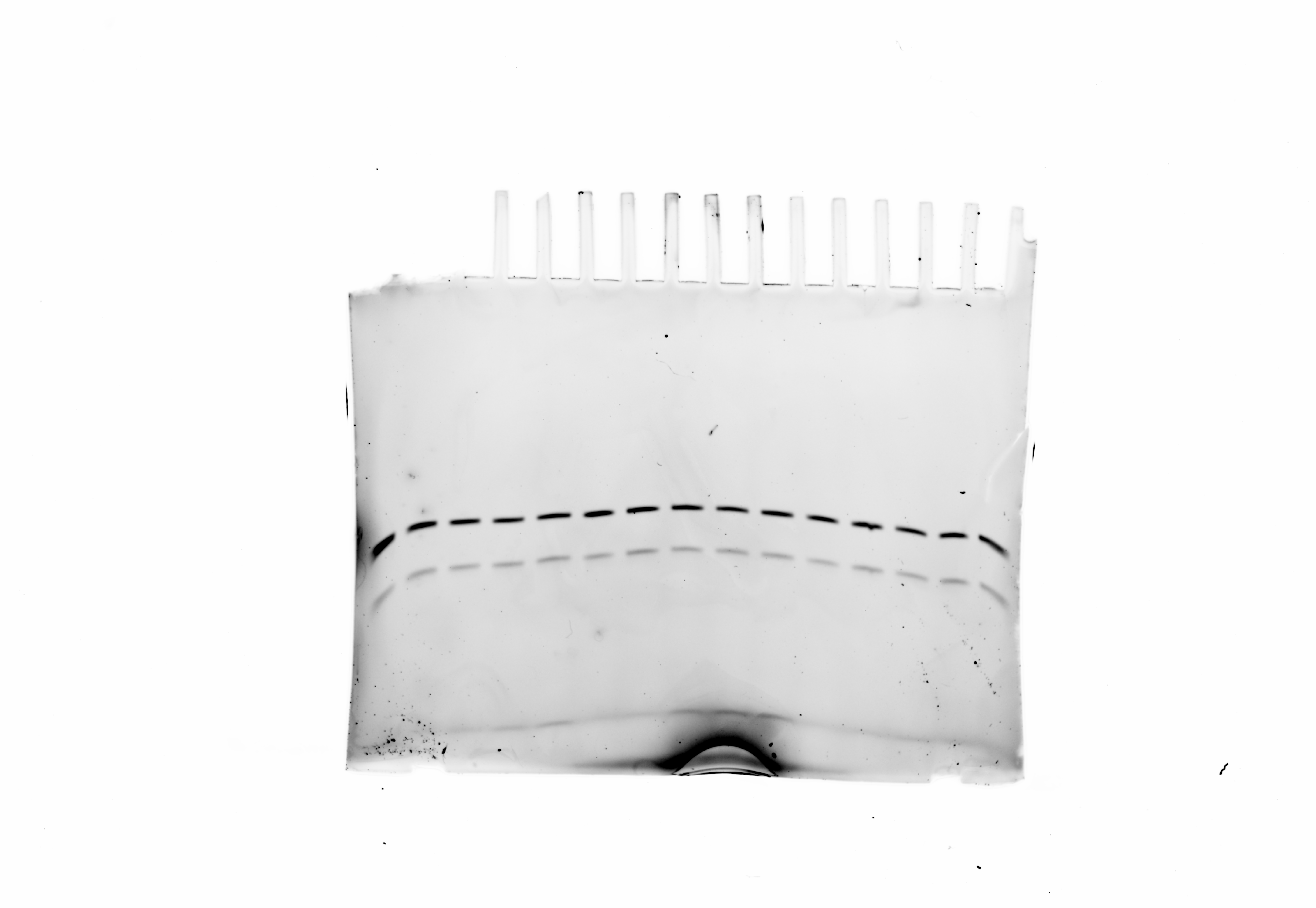

Supplement: Supplementary file 1 [file biomolecules-16-00715-s001.zip › Original-Images/FigS4B-2-G.tif]

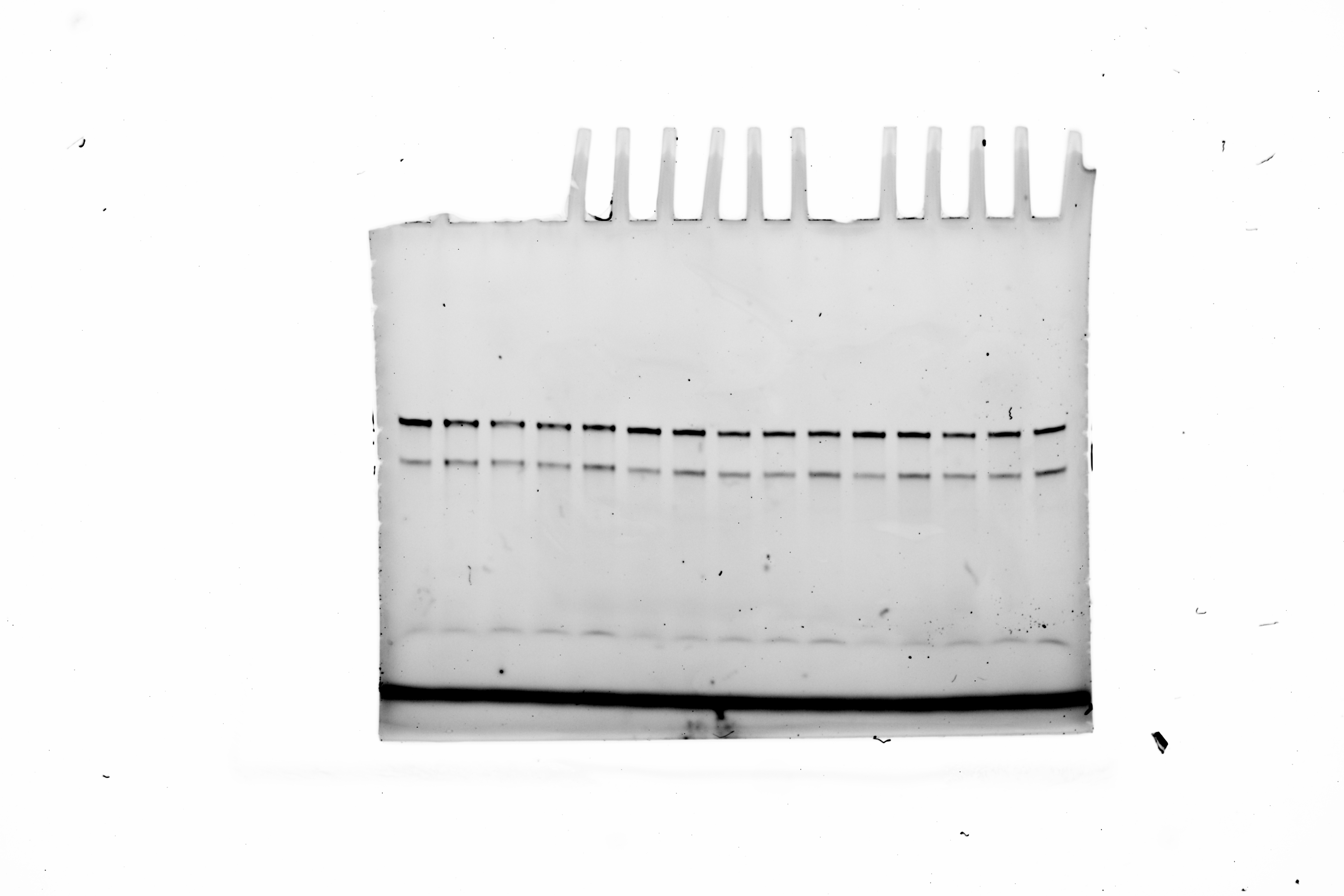

Supplement: Supplementary file 1 [file biomolecules-16-00715-s001.zip › Original-Images/FigS4B-2-T.tif]

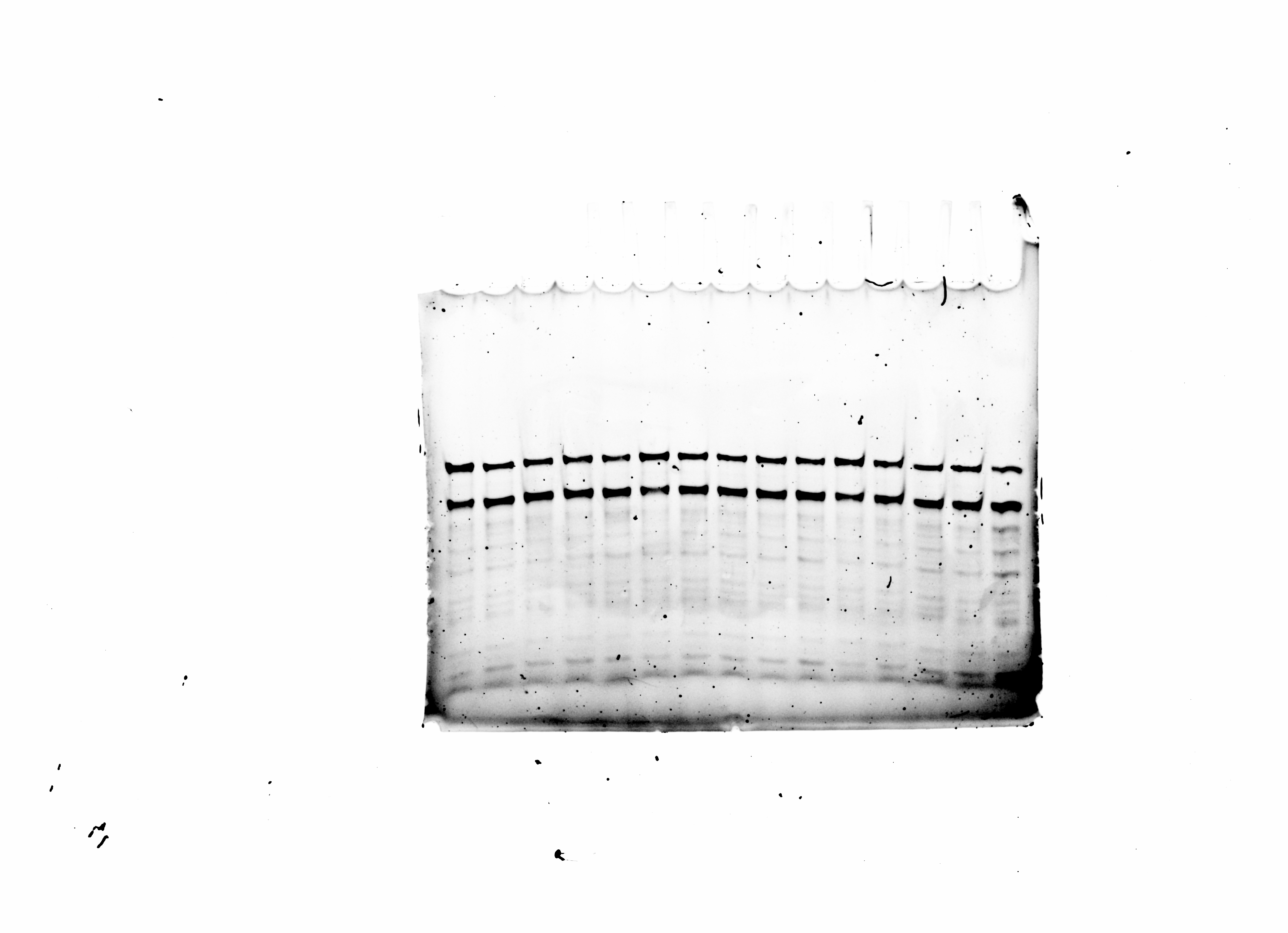

Supplement: Supplementary file 1 [file biomolecules-16-00715-s001.zip › Original-Images/FigS4B-3-A.tif]

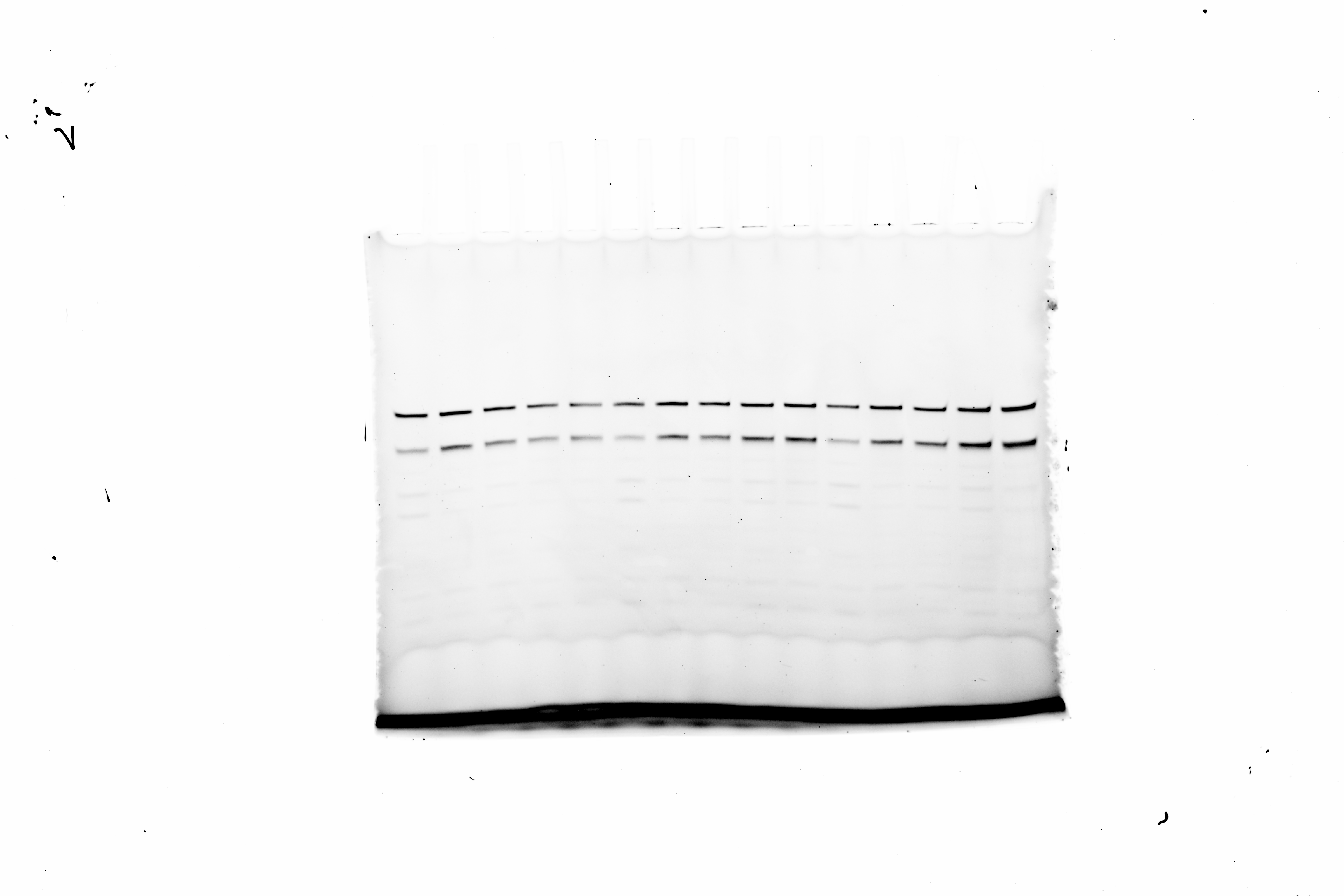

Supplement: Supplementary file 1 [file biomolecules-16-00715-s001.zip › Original-Images/FigS4B-3-C.tif]

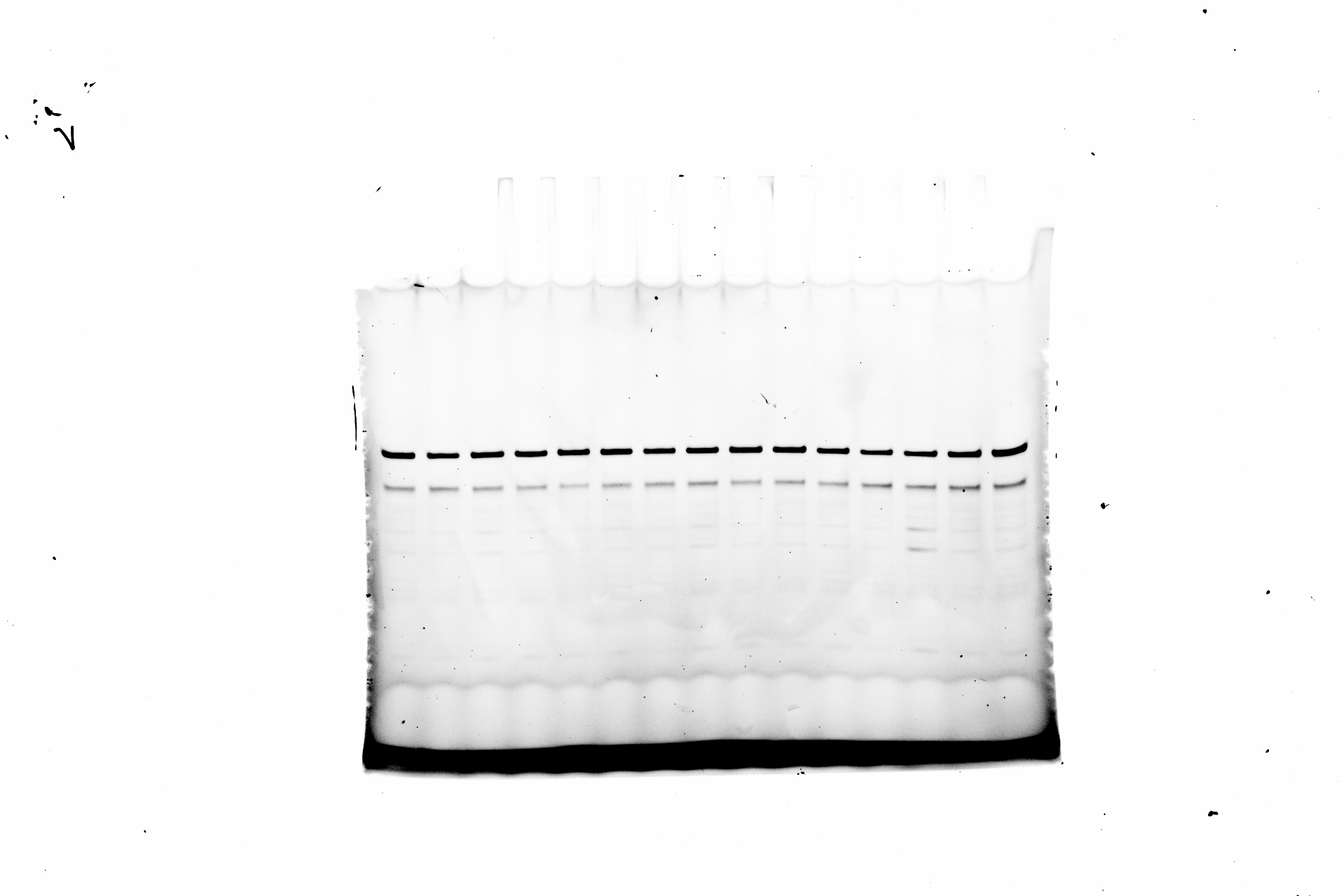

Supplement: Supplementary file 1 [file biomolecules-16-00715-s001.zip › Original-Images/FigS4B-3-G.tif]

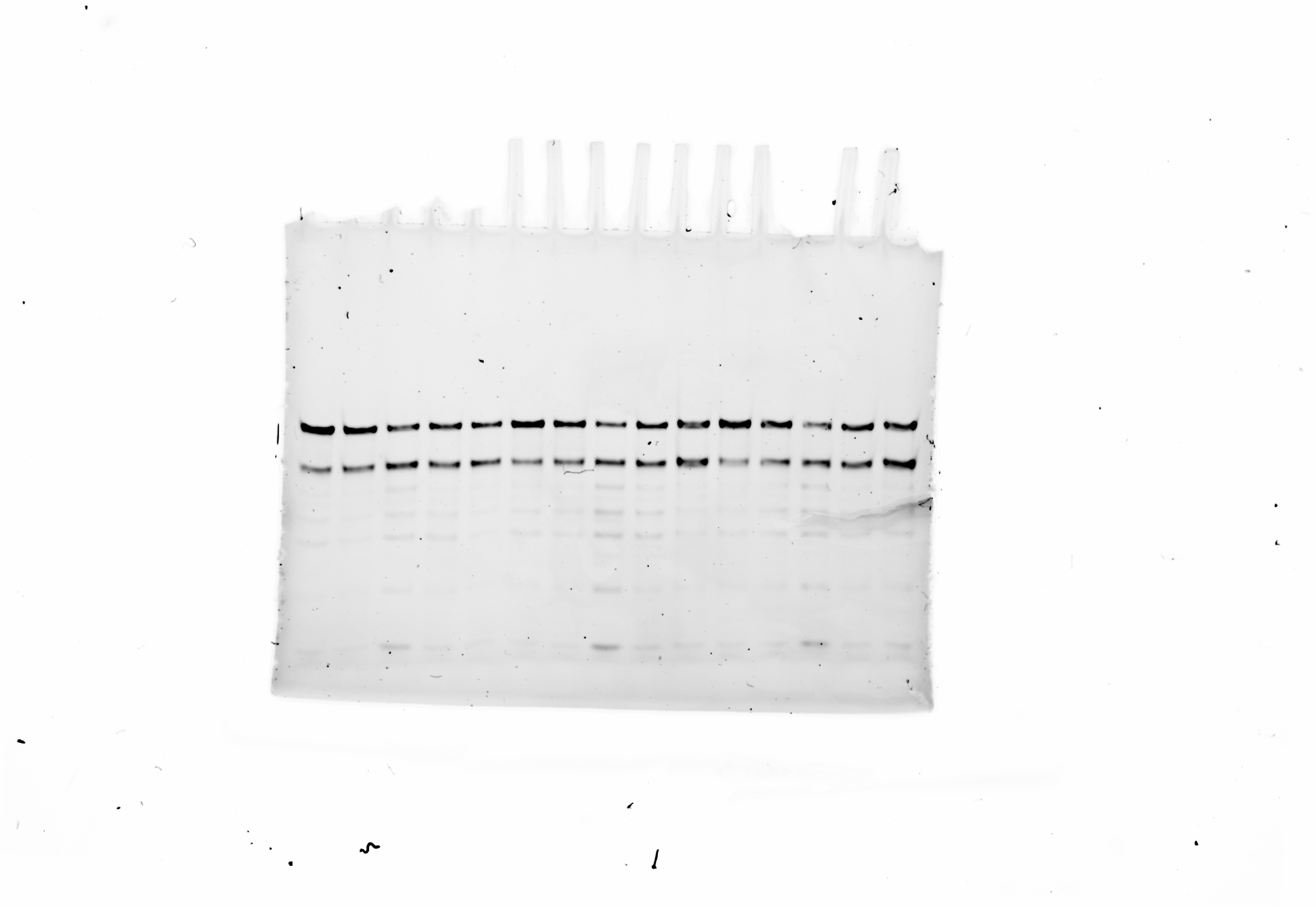

Supplement: Supplementary file 1 [file biomolecules-16-00715-s001.zip › Original-Images/FigS4B-3-U.tif]

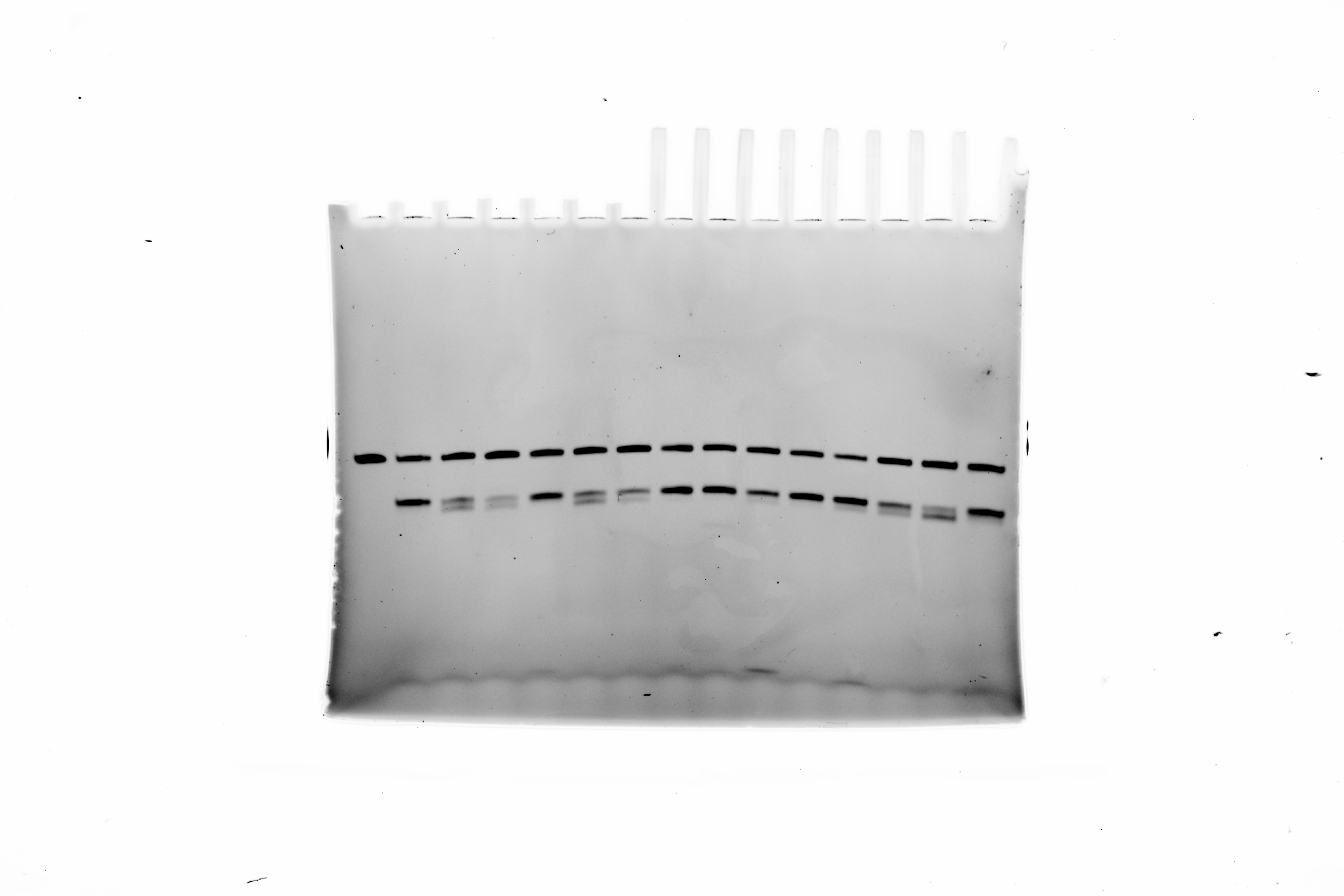

Supplement: Supplementary file 1 [file biomolecules-16-00715-s001.zip › Original-Images/FigS5-1-1.tif]

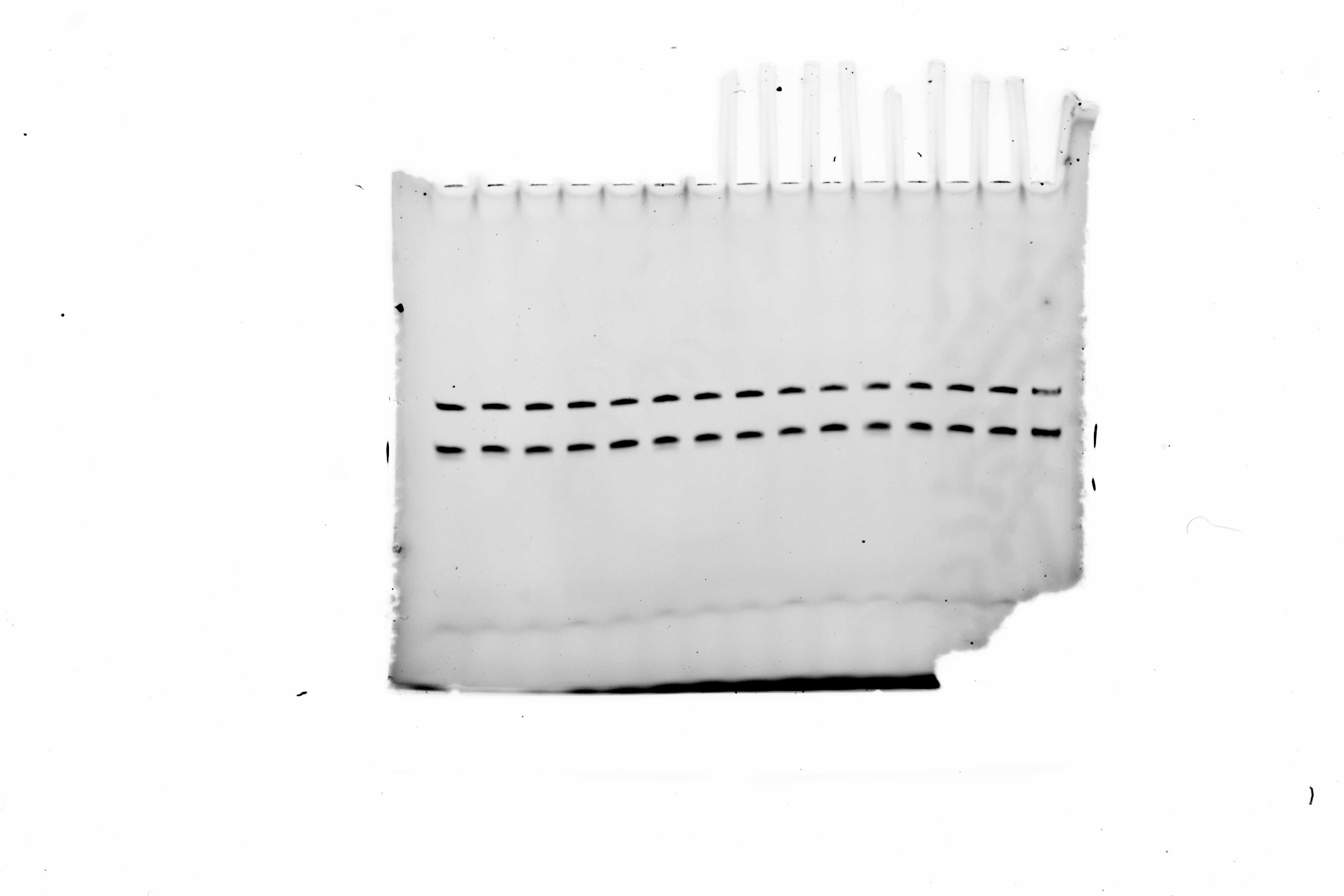

Supplement: Supplementary file 1 [file biomolecules-16-00715-s001.zip › Original-Images/FigS5-1-2.tif]

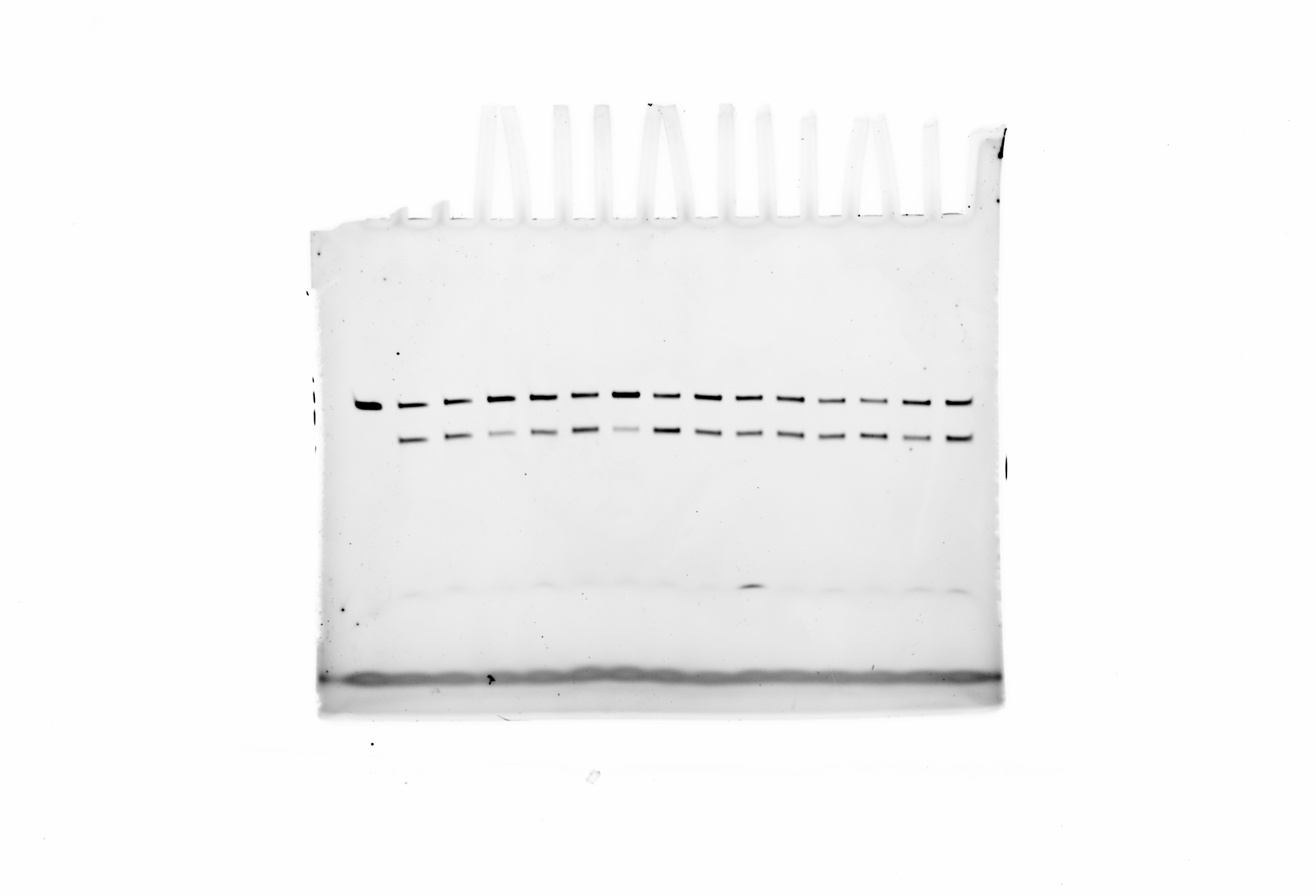

Supplement: Supplementary file 1 [file biomolecules-16-00715-s001.zip › Original-Images/FigS5-2-1.tif]

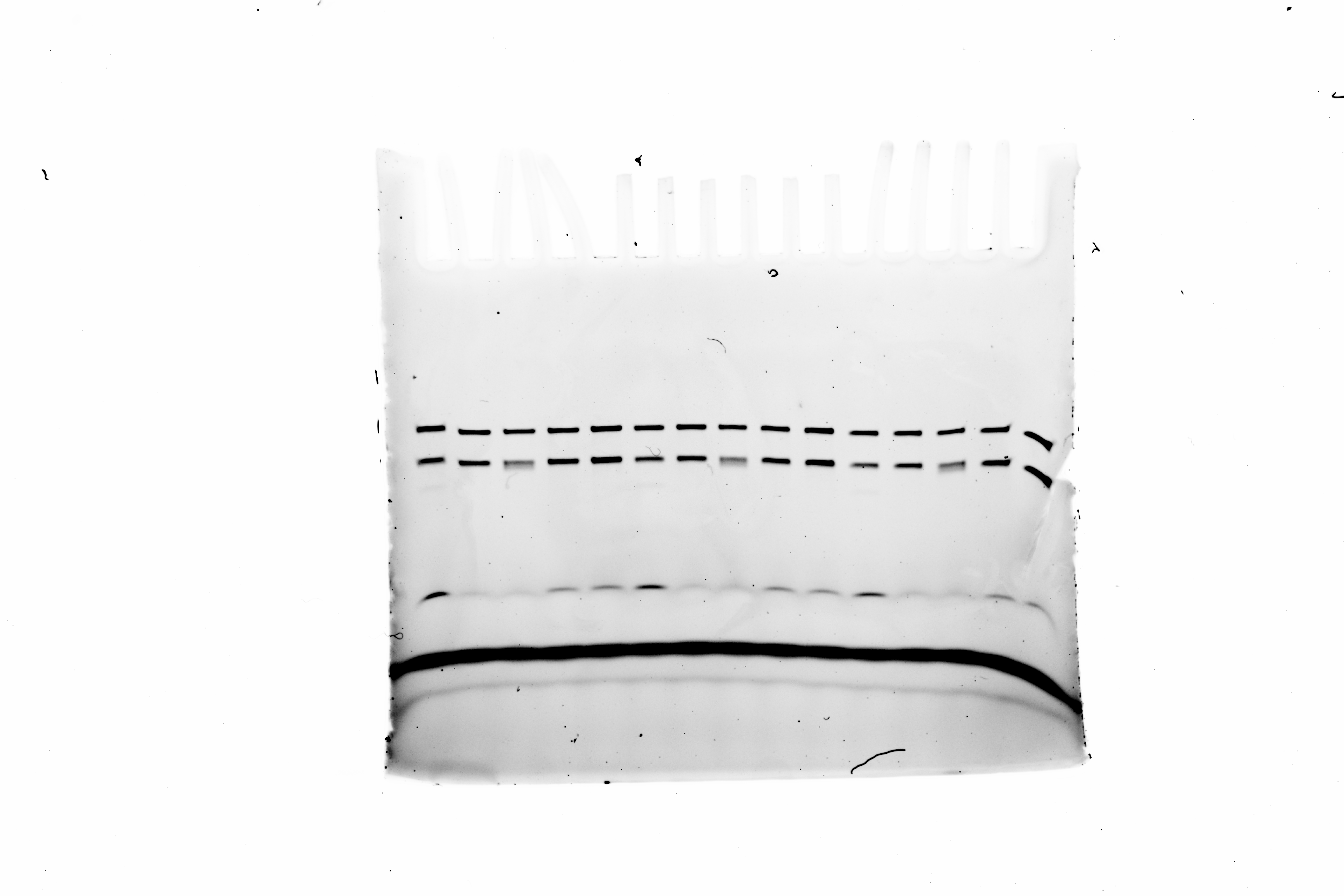

Supplement: Supplementary file 1 [file biomolecules-16-00715-s001.zip › Original-Images/FigS5-2-2.tif]

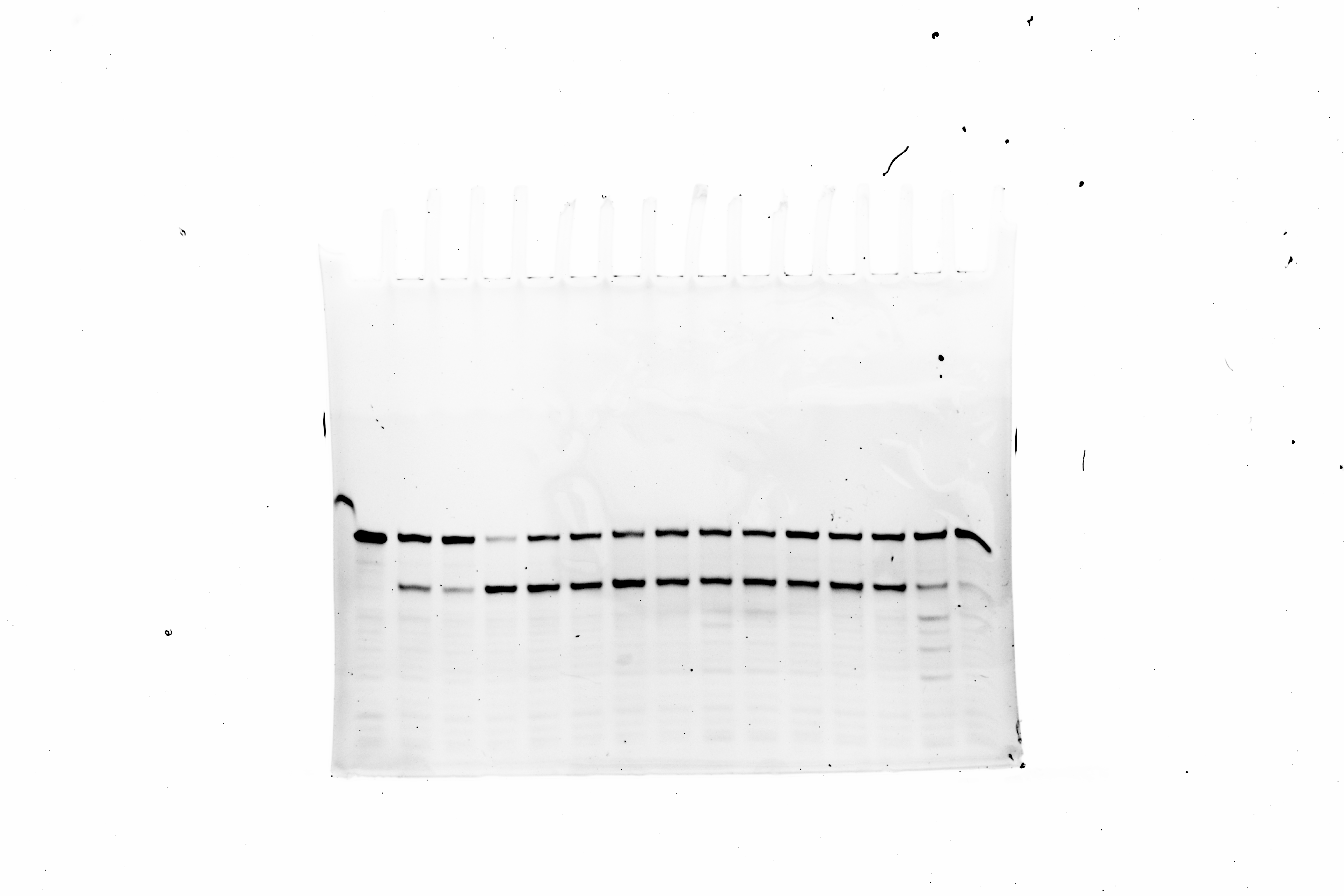

Supplement: Supplementary file 1 [file biomolecules-16-00715-s001.zip › Original-Images/FigS5-3-1.tif]

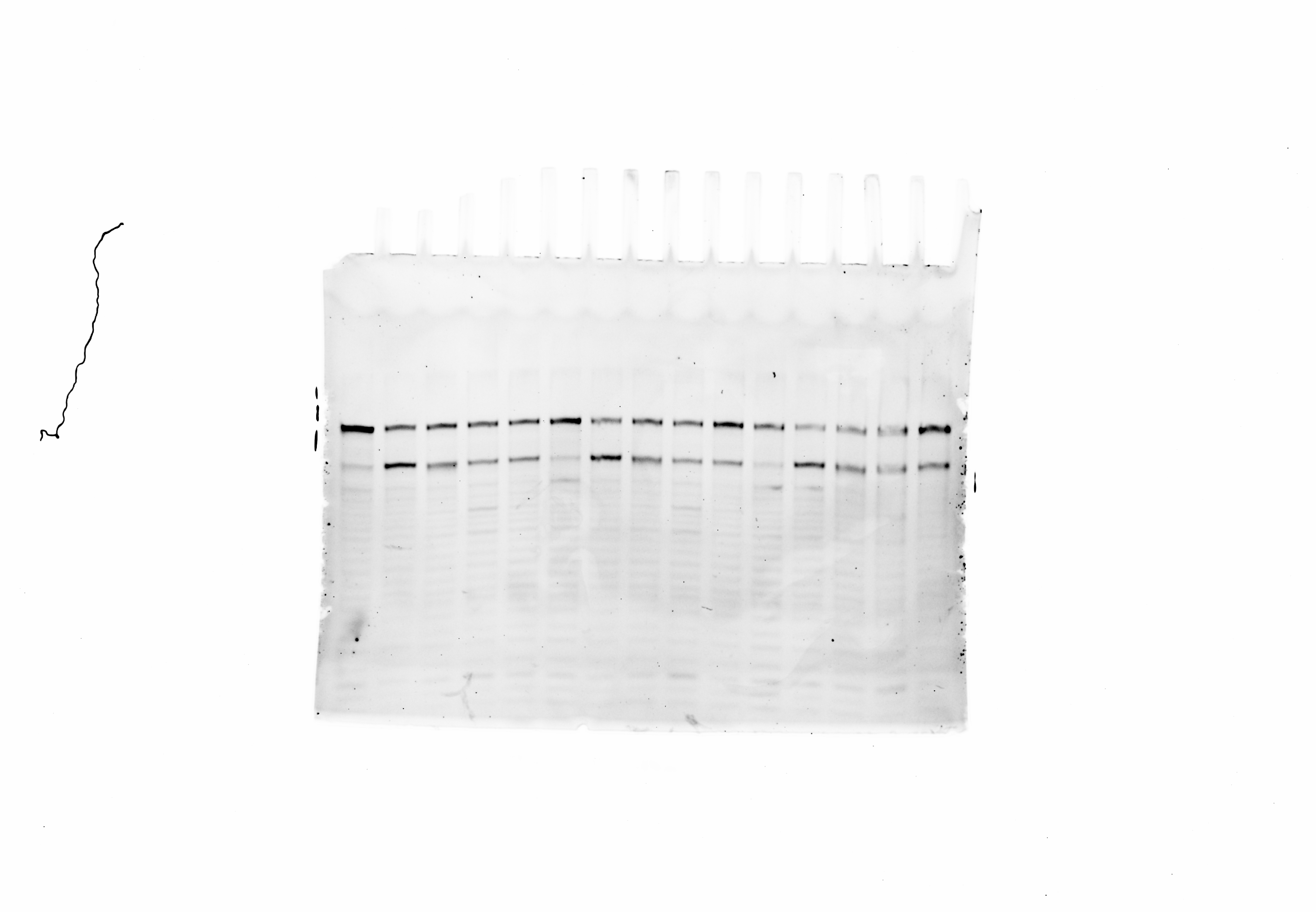

Supplement: Supplementary file 1 [file biomolecules-16-00715-s001.zip › Original-Images/FigS5-3-2.tif]

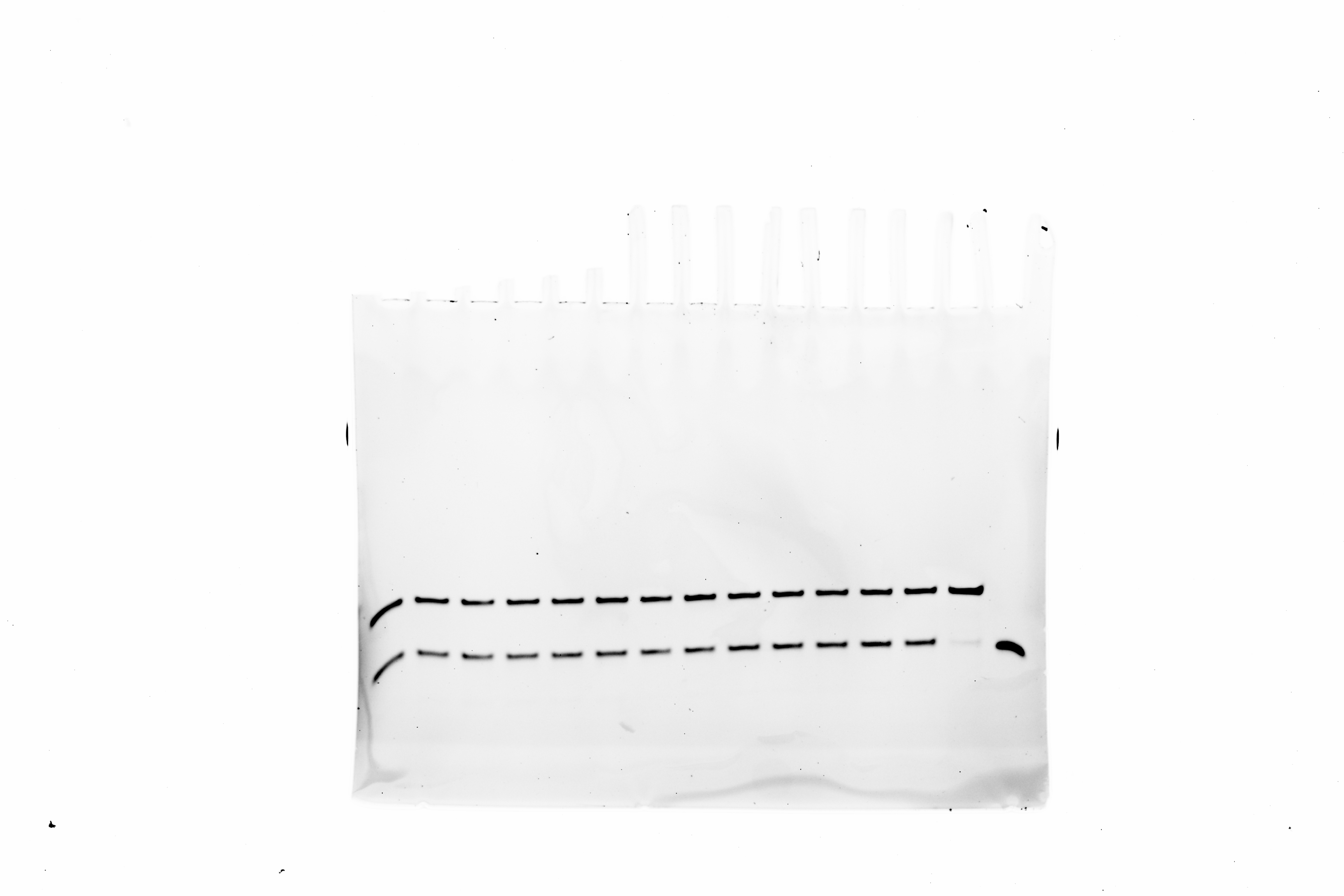

Supplement: Supplementary file 1 [file biomolecules-16-00715-s001.zip › Original-Images/FigS6A-1.tif]

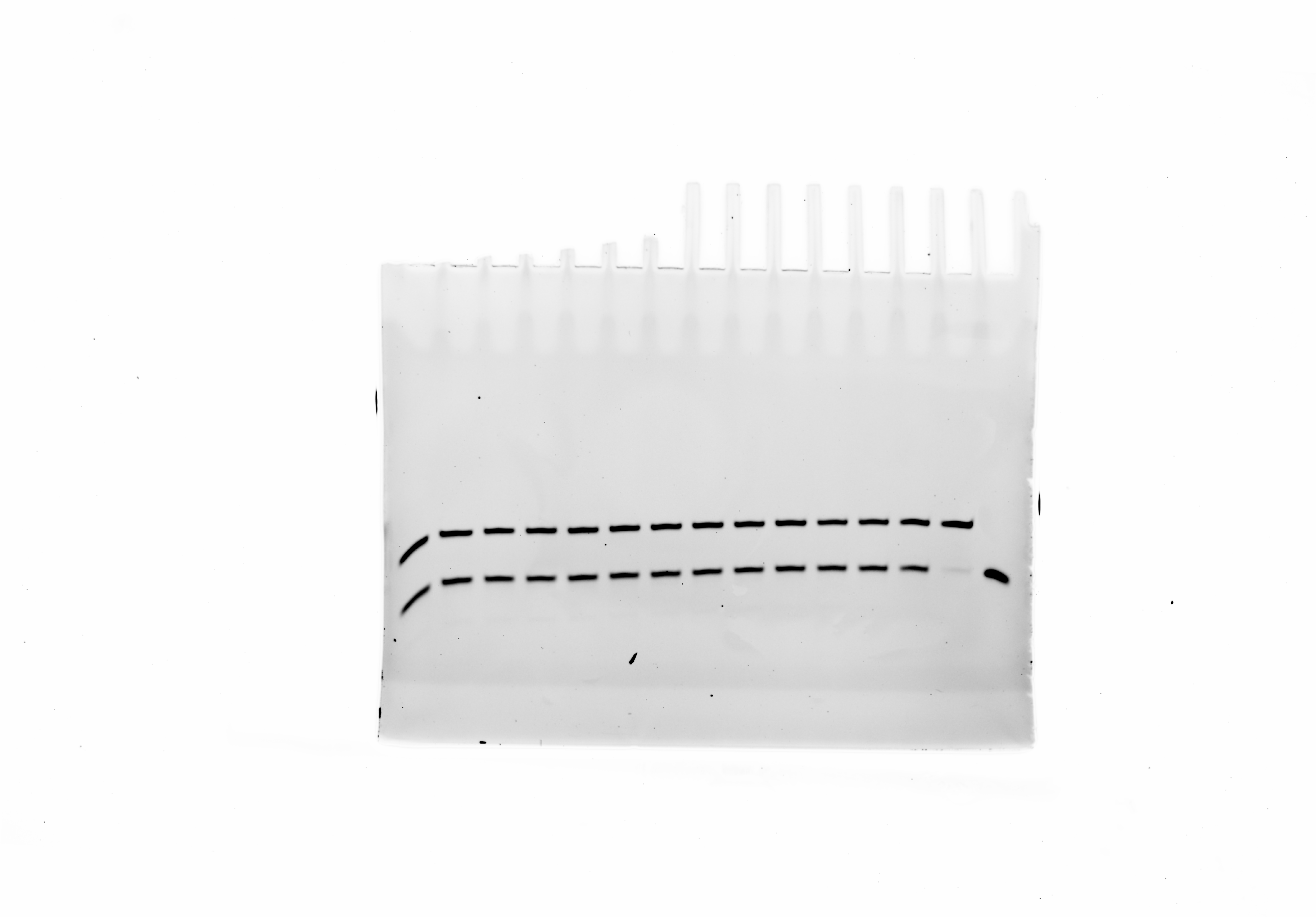

Supplement: Supplementary file 1 [file biomolecules-16-00715-s001.zip › Original-Images/FigS6A-2.tif]

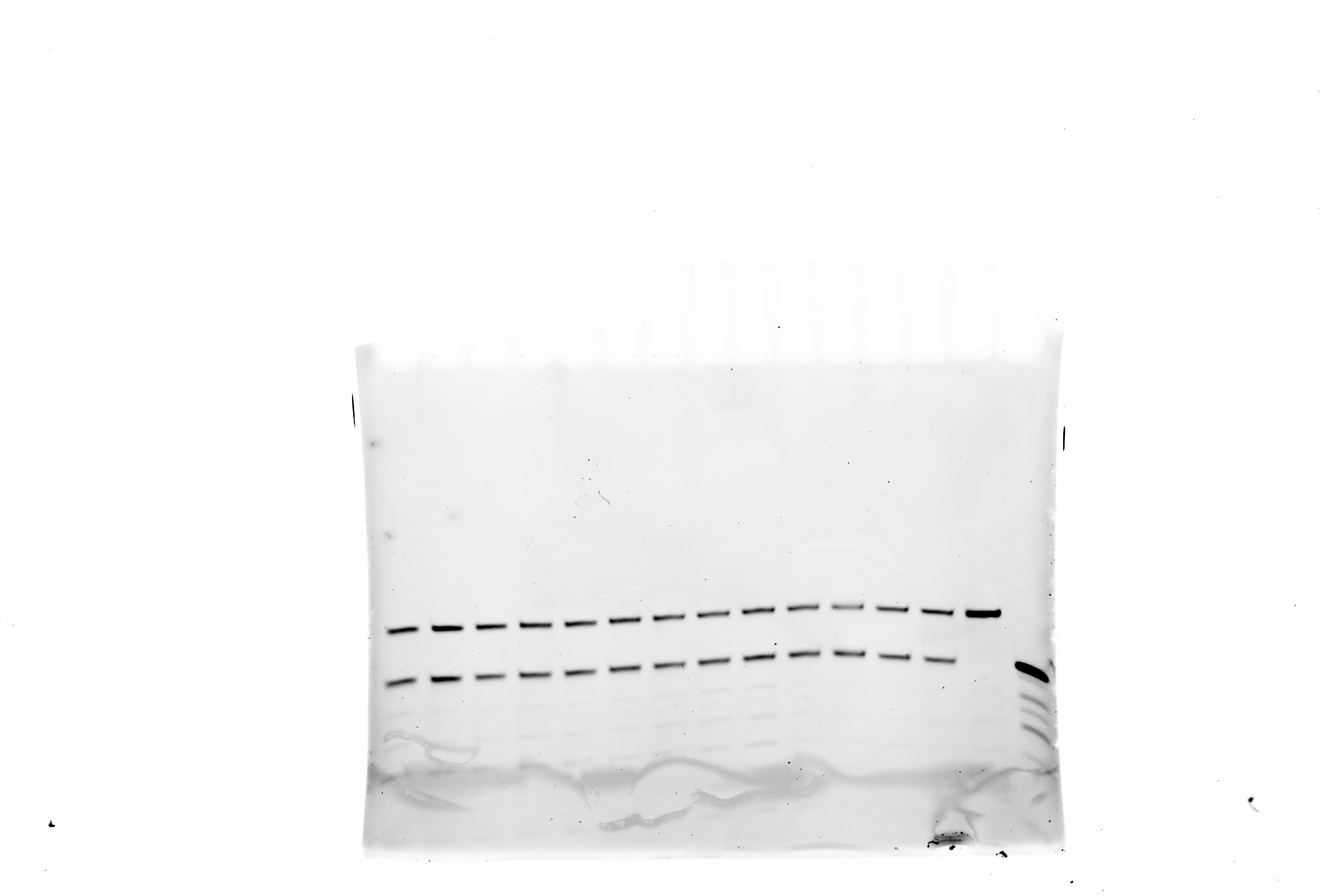

Supplement: Supplementary file 1 [file biomolecules-16-00715-s001.zip › Original-Images/FigS6A-3.tif]

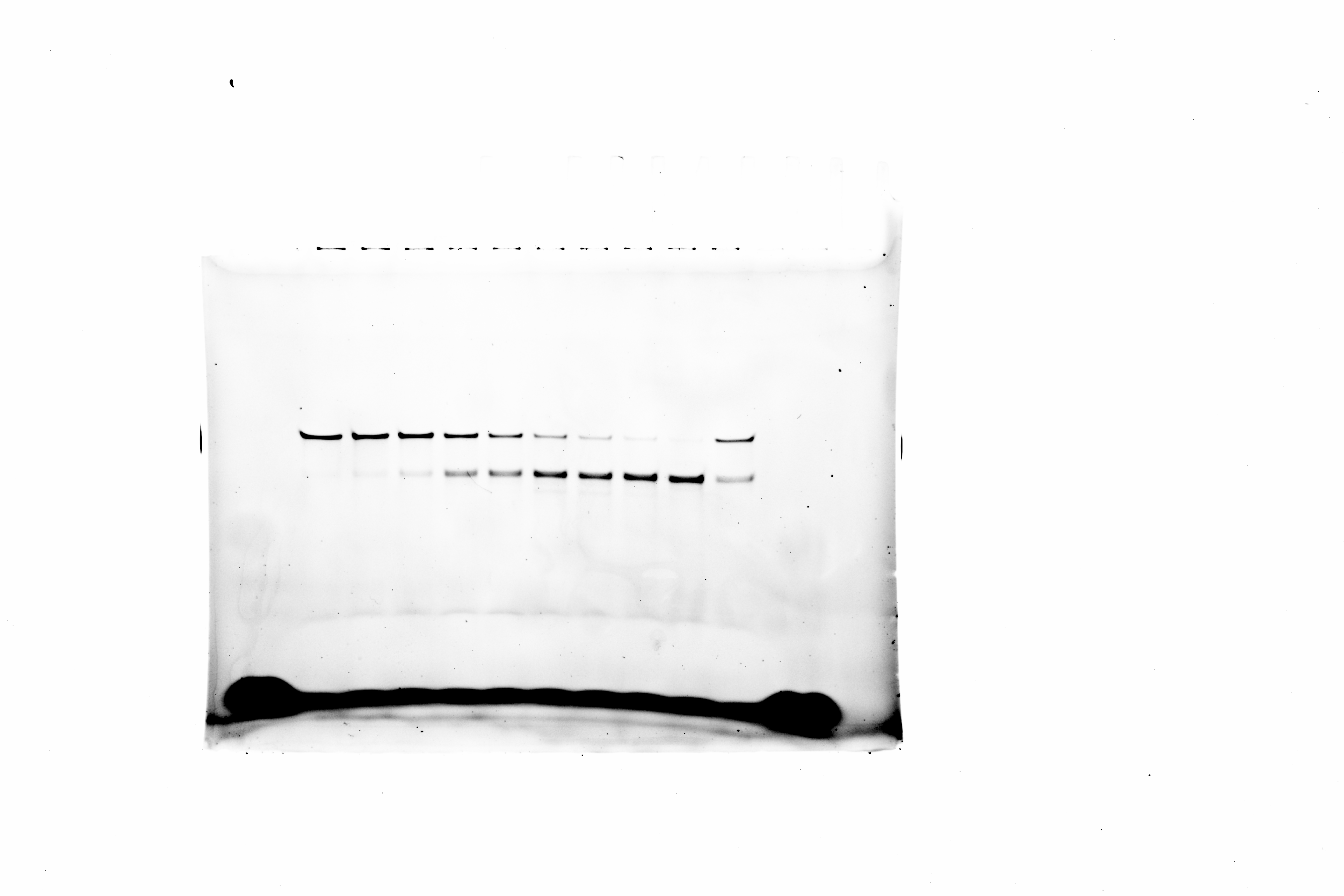

Supplement: Supplementary file 1 [file biomolecules-16-00715-s001.zip › Original-Images/FigS6B-1.tif]

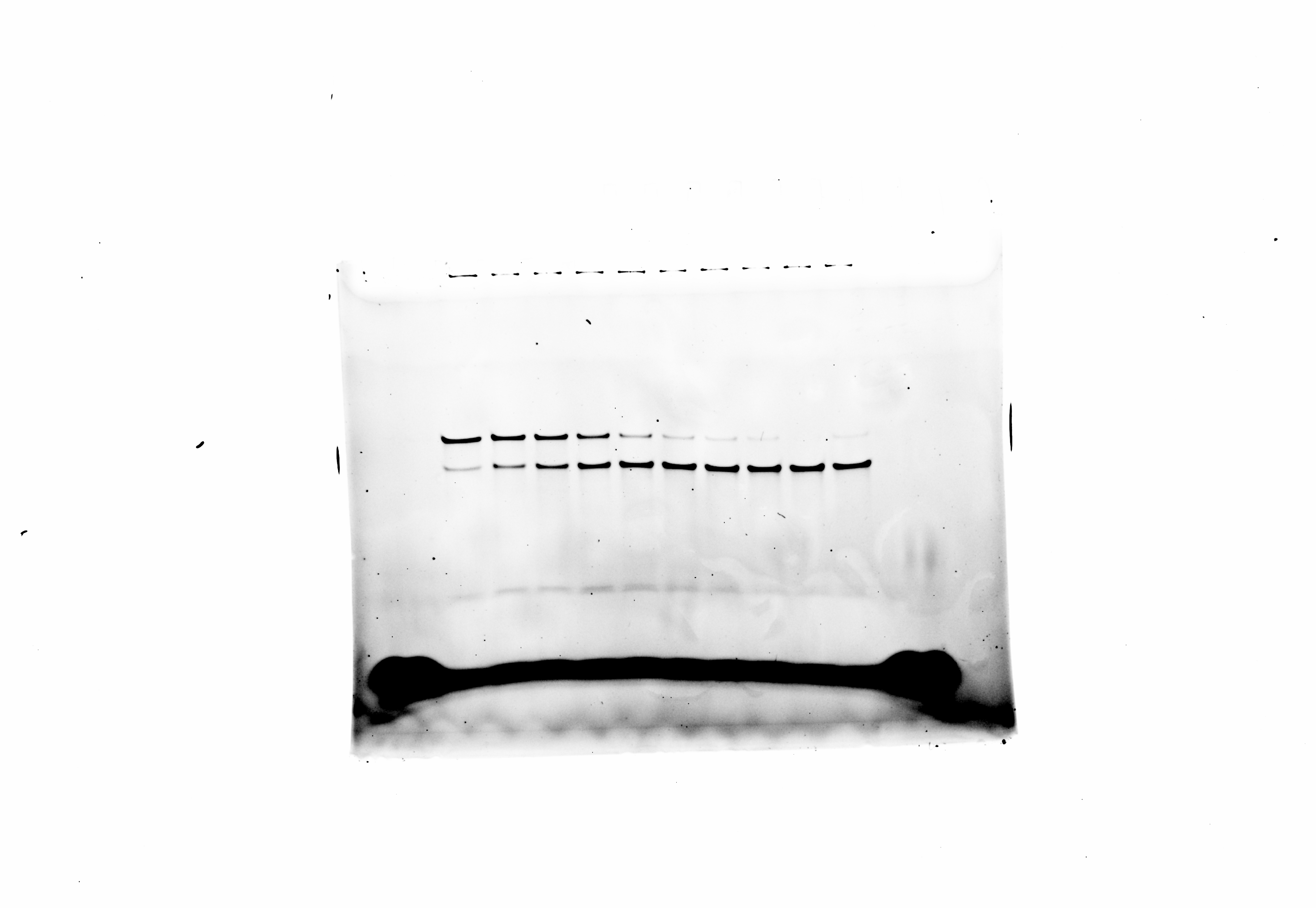

Supplement: Supplementary file 1 [file biomolecules-16-00715-s001.zip › Original-Images/FigS6B-2.tif]

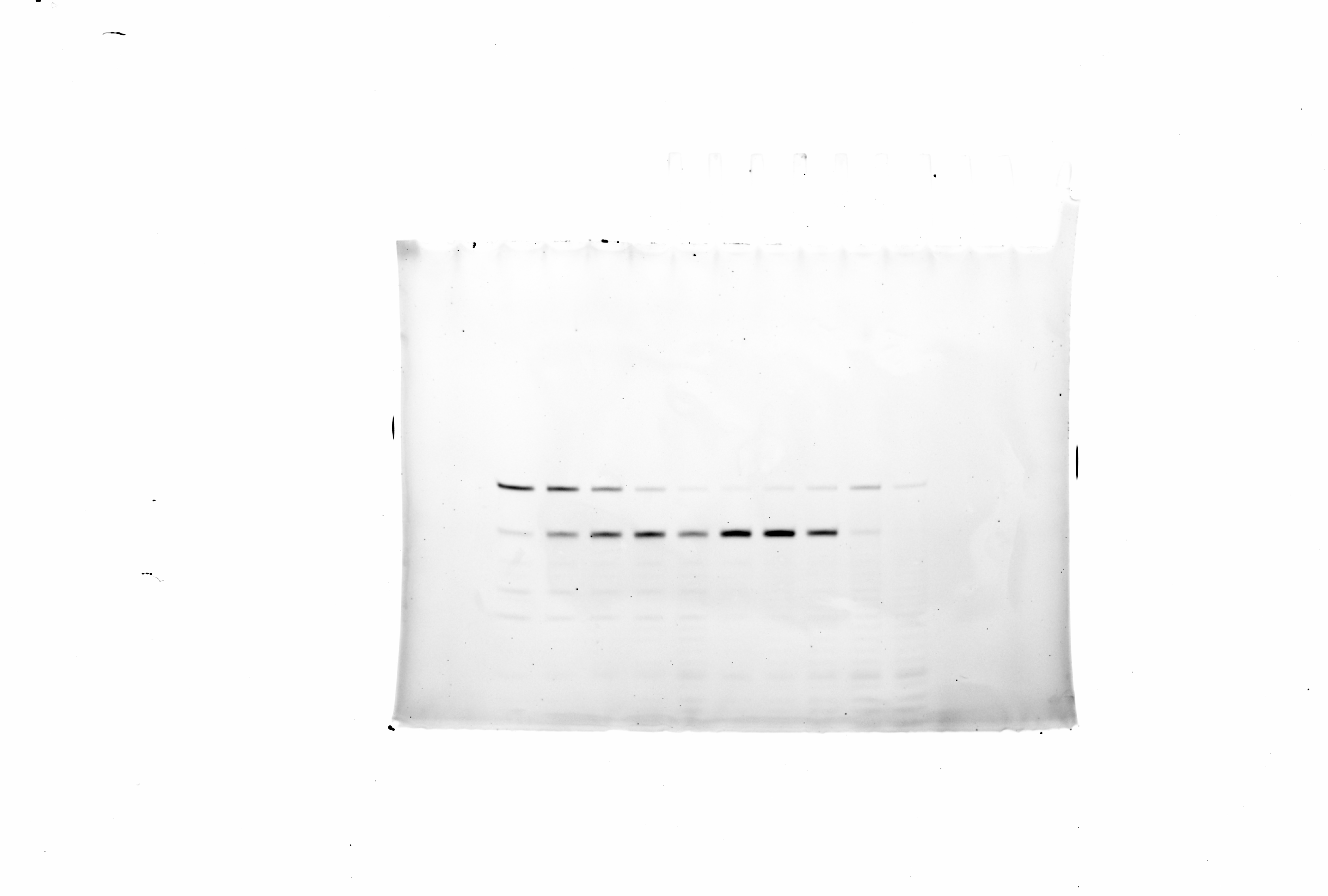

Supplement: Supplementary file 1 [file biomolecules-16-00715-s001.zip › Original-Images/FigS6B-3.tif]
